# Autophagy‐linked plasma and lysosomal membrane protein PLAC8 is a key host factor for SARS‐CoV‐2 entry into human cells

**DOI:** 10.15252/embj.2022110727

**Published:** 2022-10-04

**Authors:** Alejandro P Ugalde, Gabriel Bretones, David Rodríguez, Víctor Quesada, Francisco Llorente, Raúl Fernández‐Delgado, Miguel Ángel Jiménez‐Clavero, Jesús Vázquez, Enrique Calvo, Isaac Tamargo‐Gómez, Guillermo Mariño, David Roiz‐Valle, Daniel Maeso, Miguel Araujo‐Voces, Yaiza Español, Carles Barceló, José MP Freije, Alejandro López‐Soto, Carlos López‐Otín

**Affiliations:** ^1^ Departamento de Bioquímica y Biología Molecular, Instituto Universitario de Oncología (IUOPA) Universidad de Oviedo Oviedo Spain; ^2^ Centro de Investigación Biomédica en Red de Cáncer (CIBERONC) Madrid Spain; ^3^ Instituto de Investigación Sanitaria del Principado de Asturias (ISPA) Oviedo Spain; ^4^ Centro de Investigación en Sanidad Animal (CISA‐INIA), CSIC Valdeolmos Spain; ^5^ Centro de Investigación Biomédica en Red de Epidemiologia y Salud Pública (CIBERESP) Madrid Spain; ^6^ Centro Nacional de Investigaciones Cardiovasculares (CNIC) Madrid Spain; ^7^ CIBER de Enfermedades Cardiovasculares (CIBERCV) Madrid Spain; ^8^ Departamento de Biología Funcional, Instituto Universitario de Oncología (IUOPA) Universidad de Oviedo Oviedo Spain; ^9^ Translational Pancreatic Cancer Oncogenesis Group Health Research Institute of the Balearic Islands (IdISBa) Palma de Mallorca Spain

**Keywords:** autophagy, covid19, genetic screen, plac8, spns1, Microbiology, Virology & Host Pathogen Interaction

## Abstract

Better understanding on interactions between SARS‐CoV‐2 and host cells should help to identify host factors that may be targetable to combat infection and COVID‐19 pathology. To this end, we have conducted a genome‐wide CRISPR/Cas9‐based loss‐of‐function screen in human lung cancer cells infected with SARS‐CoV‐2‐pseudotyped lentiviruses. Our results recapitulate many findings from previous screens that used full SARS‐CoV‐2 viruses, but also unveil two novel critical host factors: the lysosomal efflux transporter SPNS1 and the plasma and lysosomal membrane protein PLAC8. Functional experiments with full SARS‐CoV‐2 viruses confirm that loss‐of‐function of these genes impairs viral entry. We find that PLAC8 is a key limiting host factor, whose overexpression boosts viral infection in eight different human lung cancer cell lines. Using single‐cell RNA‐Seq data analyses, we demonstrate that PLAC8 is highly expressed in ciliated and secretory cells of the respiratory tract, as well as in gut enterocytes, cell types that are highly susceptible to SARS‐CoV‐2 infection. Proteomics and cell biology studies suggest that PLAC8 and SPNS1 regulate the autophagolysosomal compartment and affect the intracellular fate of endocytosed virions.

## Graphical abstract

Genome‐wide CRISPR screens identify lysosomal efflux transporter SPNS1 and transmembrane protein PLAC8 as regulators of the intracellular fate of endocytosed SARS‐CoV‐2 virions.

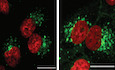

## Introduction

In March 2020, only 3 months after the detection of an outbreak of unidentified pneumonia disease in a local seafood market in the city of Wuhan (China), the World Health Organization (WHO) declared the first pandemic caused by a coronavirus (CoV). CoVs are positive‐sense single‐stranded RNA viruses that infect a variety of mammals and birds (Masters, 2006). Human endemic CoVs (HCoVs) include four strains causing the seasonal “common cold,” characterized by self‐contained mild upper respiratory tract illness. The ongoing COVID‐19 pandemic is caused by the severe acute respiratory syndrome CoV‐2 (SARS‐CoV‐2) virus, a novel CoV that likely originated in bats (Zhou *et al*, 2020). CoVs are enveloped viruses characterized by the presence of a protruding Spike protein that is essential for viral pathogenesis, since it mediates virus entry into the cell through its interaction with the ACE2 (angiotensin‐converting enzyme 2) cellular receptor. The Spike protein is a heavily glycosylated transmembrane protein that forms homotrimers in the membrane of mature virions (Jackson *et al*, 2022). This protein is composed of two subdomains called S1 and S2. In the prefusion state, the S1 subdomains, which contain the receptor binding domain (RBD), wrap around a central core formed by the S2 subdomains. Sequential cleavage of the S1/S2 boundary and the S2′ site of the S2 subunit by host proteases triggers a series of conformational changes that result in the disengagement of the S1 subunit and exposure of the S2 subunits, which mediate the fusion of the virion membrane and the release of the viral genome. Current models suggest that the furin site present in the SARS‐CoV‐2 S1/S2 boundary enables precleavage of the Spike protein during virion egression in the producer cell (Peacock *et al*, 2021). Upon binding of the preactivated Spike to ACE2, a second proteolytic event at the S2′ site completes the activation of the Spike protein and triggers membrane fusion. Depending on the protease repertoire of the target cells, two different entry routes have been proposed (Shang *et al*, 2020; Murgolo *et al*, 2021; Cesar‐Silva *et al*, 2022; Jackson *et al*, 2022; Rebendenne *et al*, 2022). In cells with high levels of type II transmembrane proteases, such as TMPRSS2, cleavage at the S2′ occurs at the plasma membrane of the target cell and the viral genome is directly released to the cytoplasm (Hoffmann *et al*, 2020; Koch *et al*, 2021; Winstone *et al*, 2021). In the other route, virions are internalized through clathrin‐mediated endocytosis and activation of the Spike protein occurs in the late endosome/lysosome pathway by members of the cathepsin family of acid proteases (Bayati *et al*, 2021; Gorshkov *et al*, 2021; Li *et al*, 2021; Ou *et al*, 2021; Yang *et al*, 2022).

This is the third and largest outbreak of a zoonotic CoV, after SARS‐ and MERS‐CoVs, and has caused dramatic health, social and economic crisis worldwide. Although vaccines have helped to contain virus spread and deaths, the emergence of resistant strains and the lack of effective specific treatments to block viral infection pose a continuous risk. Therefore, fully understanding host‐virus interactions and identifying potentially targetable host factors are key approaches to complement and improve the use of vaccines in tackling this disease. In this work, we report a genome‐wide CRISPR screen for SARS‐CoV‐2 entry host factors using a simplified model consisting of Spike‐pseudotyped lentiviruses, a system that can be used in class‐2 biosafety laboratories. Notably, our system has recapitulated and extended many discoveries reported in recent screens using full SARS‐CoV‐2 virus and has helped to define their specific role in viral entry. Moreover, we have identified and validated two novel host factors that are required for this process: PLAC8 and SPNS1. Finally, we have found that PLAC8 is a key and limiting host factor that is highly expressed in lung ciliated cells and whose overexpression boosts SARS‐CoV‐2 infection in multiple lung cell lines.

## Results

### Genome‐wide CRISPR knockout screen identifies novel host factors for SARS‐CoV‐2 entry

**Figure 1:**
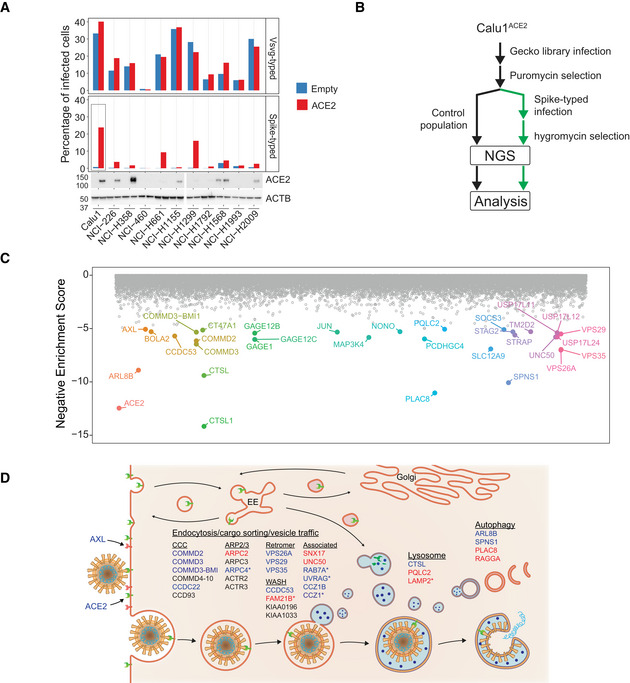
A genome‐wide CRISPR screen for SARS‐CoV‐2 entry host factors Infection susceptibility (top) to VSVG and Spike‐typed lentiviruses of a panel of lung cancer cell lines with or without (Empty vector) overexpression of *ACE2*. ACE2 protein levels were determined by Western blot (bottom).Screen strategy: Calu1^ACE2^ cells were transduced with the GeckoV2 CRISPR library to create a mutant library that was subsequently infected with Spike‐typed lentiviruses carrying a hygromycin‐resistant plasmid. After hygromycin selection, the dropout of sgRNAs in the infected population compared with an uninfected population was assessed by NGS.Gene‐level scores obtained by robust ranking algorithm analysis of the sgRNA relative abundances between cells infected with Spike‐typed lentiviruses and uninfected cells. Genes were ordered alphabetically on the x‐axis and the top hits (log10(enrichment‐score) < −5) were colored and labeled. Note that *CTSL* and *CTSL1* are different gene isoforms.Screen results integration in pathways previously linked to SARS‐CoV‐2 entry. Black: nonsignificant genes reported by others; Blue: significant genes previously reported; Red: novel genes not reported in previous screens. *Nonsignificant based on FDR (FDR > 0.05) but present in the top 200 hit list. Source data are available online for this figure.

To screen for host factors involved in SARS‐CoV‐2 entry, we first set up a cellular model using lentiviruses pseudotyped with the Spike protein (hereafter S‐typed). To do that, we overexpressed the gene encoding the viral receptor *ACE2* in several human lung cancer cell lines and tested their susceptibility to infection by means of a fluorescence reporter (Fig 1A). To enhance experimental performance, we used the Spike‐Δ19 mutant, which has been reported to increase viral titer in lentiviral models (Johnson *et al*, 2020). As a control, we infected the same cell lines with Vesicular stomatitis virus G (VSVG)‐pseudotyped (hereafter VSVG‐typed) lentiviruses. In line with other works that reported restricted *ACE2* expression in lung tissues (Lukassen *et al*, 2020), most of the tested cell lines displayed low basal levels of infection that were boosted when *ACE2* was overexpressed, while their susceptibility to infection with VSVG‐typed lentivirus was generally high and independent of ACE2 levels. Among them, the non‐small‐cell lung cancer cell line Calu1 showed the highest infection susceptibility to S‐typed lentiviruses upon overexpression of *ACE2* (hereafter Calu1^ACE2^) and was therefore selected for a genome‐wide CRISPR screening.

To perform the knockout screen, Calu1^ACE2^ cells were first transduced with the GeCKO v2.0 CRISPR‐CAS9 library at a 0.3 multiplicity of infection (MOI) and 1,000× library coverage. Calu1^ACE2^‐GeCKO cells were subsequently infected with Spike‐Δ19 pseudotyped lentiviral particles carrying a plasmid that confers resistance to hygromycin. After 10 days of hygromycin selection, the sgRNA abundance was assessed by next‐generation sequencing (NGS) of their genomic DNA and compared with a population of uninfected Calu1^ACE2^‐GeCKO cells that were cultured in parallel to filter out sgRNAs that affect cellular fitness (Fig 1B). Robust rank aggregation analysis of the sgRNA relative abundances identified 104 targets (corresponding to 67 genes) whose loss‐of‐function was significantly depleted in the Spike‐infected cells compared with the control population (Fig 1C; Dataset EV1). The viral receptor gene *ACE2* ranked as the second least depleted gene in Spike‐infected cells, just preceded by *CTSL*, which encodes the lysosomal protease implicated in Spike cleavage and viral membrane fusion. Likewise, we detected multiple genes linked to endocytosis and vesicle trafficking that have been reported during the elaboration of this manuscript in other CRISPR screens for SARS‐CoV‐2 host factors. Our findings include several genes encoding the endosomal Retromer (*VPS26A*, *VPS29*, and *VPS35*) and the associated complexes CCC (*CCDC22*, *COMMD2*, *COMMD3*, and *COMMD3‐BMI1*), WASH (*CCDC53*), and ARP2/3 (*ARPC2*), candidate viral co‐receptors (*AXL*), cell–cell adhesion molecules (*PCDHGB1* and *PCDHGC3*), and autophagy regulators (*ARL8B* and *SPNS1*) (Daniloski *et al*, 2021; Hoffmann *et al*, 2021; Schneider *et al*, 2021; Wei *et al*, 2021; Zhu *et al*, 2021; Wang *et al*, 2021a, 2021b; Fig 1C and D; Dataset EV1). We also found different components of the JAK–STAT pathway (*STAT2*, *TYK2*, and *JAK1*) among the host factors whose loss‐of‐function favors SARS‐CoV‐2 infection. This signaling pathway mediates cellular responses to multiple cytokines and growth factors and has been reported to be important in the context of SARS‐CoV‐2 infection. Taken together, these findings demonstrated the technical quality of our screen and the adequacy of our pseudotyped lentiviral model to identify novel candidate host factors important for SARS‐CoV‐2 entry. In fact, upon detailed analysis of the genome‐wide CRISPR knockout screening results, we detected a series of novel candidates including genes that participate in signaling pathways or structural complexes associated with SARS‐CoV‐2 entry. Among them, there are members of the ARP2/3 complex (*ARPC2*), WASH complex (*FAM21B*), vesicle transport (*SNX17* and *UNC50*), lysosomal proteins (*PQLC2* and *LAMP2*), autophagy‐associated factors (*PLAC8* and *RAGGA*), cell–cell junction (multiple protocadherin genes), and genes not directly linked to viral entry (e.g., *NONO* and *SLC1A12*).

Interestingly, two genes that have not been yet characterized in the context of SARS‐CoV‐2 entry, *PLAC8*, and *SPNS1*, rank immediately after *ACE2* and *CTSL* in our screen. Remarkably, *SPNS1* (spinster homolog 1) was recently reported as one of the positive genes during a screen for SARS‐CoV‐2 host factors but was not further analyzed or functionally validated (Zhu *et al*, 2021), while *PLAC8* (placenta associated 8) has not been identified as a candidate host factor in any of the various genetic screens already published (Baggen *et al*, 2021; Daniloski *et al*, 2021; Hoffmann *et al*, 2021; Schneider *et al*, 2021; Wei *et al*, 2021; Zhu *et al*, 2021; Wang *et al*, 2021a, 2021b). *SPNS1* encodes a lysosomal efflux transporter that regulates mTOR signaling and whose loss‐of‐function is associated with lysosome storage disorders (Nakano *et al*, 2001; Dermaut *et al*, 2005; Rong *et al*, 2011; Sasaki *et al*, 2014; Yanagisawa *et al*, 2017). *PLAC8* encodes a small transmembrane protein that localizes both in the plasma membrane and in lysosomes, where it has been associated with autophagosome fusion (Kinsey *et al*, 2014; Kolluru *et al*, 2017; Segawa *et al*, 2018; Feng *et al*, 2021). Given the association of *PLAC8* and *SPNS1* with autophagy and lysosome function, their high‐ranking position in our screen, and the dependency on the endolysosomal pathway for the entry of other coronaviruses (Mingo *et al*, 2015), we decided to follow up on these two hits and validate them using an orthogonal approach.

### PLAC8 and SPNS1 specifically affect S‐typed lentiviral entry

**Figure 2:**
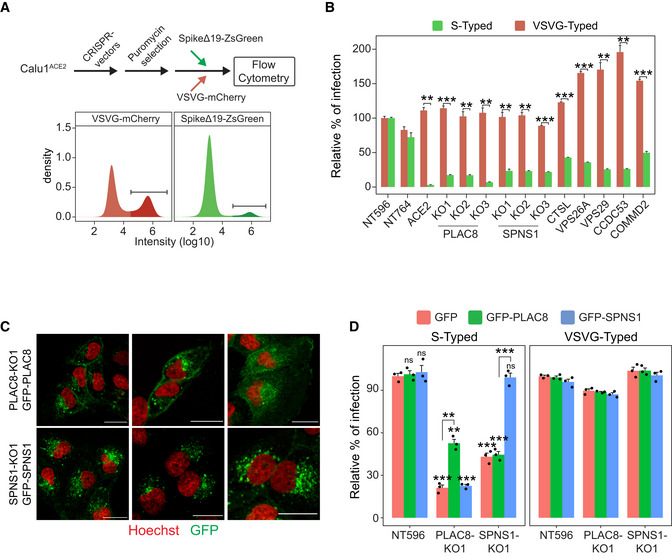
Validation of screen results with pseudotyped lentiviruses Scheme depicting the screen validation strategy (top) and representative density plots (bottom) of flow cytometry data from cells infected with VSVG‐ or Spike‐typed lentivirus. The infected population is labeled with a darker color.Susceptibility to infection with Spike‐typed and VSVG‐typed lentiviruses in Calu1^ACE2^ CRISPR KO cell lines with loss‐of‐function of selected screen candidates. NT596 and NT764 are two nontargeting CRISPR controls. ACE2 are Calu1^ACE2^ cells transduced with a CRISPR vector against *ACE2*. Bars represent the average and standard error of the mean (SEM) of the percentage of infected cells in each condition (three biological replicates), normalized to the nontargeting control cell line NT596. The *t*‐test *P*‐value between the relative percentage of infection with Spike‐typed and VSVG‐typed for each cell line is indicated.Confocal microscopy images of *SPNS1*‐KO1 and *PLAC8*‐KO1 Calu1^ACE2^ cells overexpressing CRISPR‐resistant *GFP‐SPNS1* or *GFP‐PLAC8*, respectively. Scale bar: 20 μm.Rescue experiments in *PLAC8* and *SPNS1* Calu1^ACE2^ KO cell lines: bar plot showing the average (three biological replicates) and SEM percentage of infection (normalized to NT596 with *GFP* overexpression) of Spike‐typed and VSVG‐typed lentiviruses in *PLAC8*‐KO1 and *SPNS1*‐KO1 cell lines that overexpress CRISPR‐resistant *GFP‐PLAC8* or *GFP‐SPNS1* constructs. The significance above each bar represents the *t*‐test *P*‐value between each condition and the control cell line (NT596 with *GFP* overexpression). Data information: ns, *P*‐value ≥ 0.05, *P‐value < 0.05, **P‐value < 0.01, ***P‐value < 0.001. Source data are available online for this figure.

As a first step to validate our selected candidates, we generated individual *PLAC8* and *SPNS1* knockout (KO) Calu1^ACE2^ cell lines using three different sgRNAs for each of these genes and infected them with S‐typed lentiviruses carrying a ZsGreen‐expressing plasmid to allow assessment of infection efficiency by flow cytometry (Fig 2A). Knockout levels of PLAC8 and SPNS1 were assessed by Western blot (Appendix Fig S1A). To rule out that these two genes are involved in normal lentiviral biology, we infected the same cell lines with VSVG‐typed lentiviruses carrying an mCherry‐expressing plasmid. As a positive control, we included Calu1^ACE2^ knockout cell lines for *ACE2* and *CTSL*, as well as for four genes that participate in known SARS‐CoV‐2 entry pathways. In agreement with our screen results, loss‐of‐function cell lines of all genes tested displayed a strong decrease in infection efficiency using S‐typed lentiviruses compared with control cells (Calu1^ACE2^ cells transduced with two different control sgRNAs) (Fig 2B). As expected, *ACE2*‐KO showed the strongest effect, rendering the cells virtually resistant to viral infection, while similar levels to those of nontargeting control cells were observed when using viruses that enter through a different receptor (VSVG‐typed lentiviruses). Notably, all *PLAC8* and *SPNS1* KO cell lines showed a dramatic reduction in infection efficiency using S‐typed lentiviruses, reaching higher fold‐change levels than any positive control (except for *ACE2*‐KO). However, cell susceptibility to infection with VSVG‐typed lentiviruses was not affected in any case, ruling out the involvement of these two genes in normal lentiviral biology.

To reinforce these results, we performed rescue experiments by ectopically expressing CRISPR‐resistant GFP‐fused versions of *PLAC8* and *SPNS1* on the CRISPR KO cell lines. Western blot analyses confirmed the knockdown levels of endogenous PLAC8 and SPNS1 and the overexpression of the GFP‐fused versions (Appendix Fig S1B). Confocal microscopy analysis demonstrated specific distribution patterns of GFP‐PLAC8 and GFP‐SPNS1 (Fig 2C). Thus, GFP‐PLAC8 localized both in punctuated structures and in the plasma membrane, and GFP‐SPNS1 displayed a predominantly perinuclear punctuated pattern. Importantly, *in vitro* infection assays with S‐typed lentiviruses confirmed that the overexpression of *GFP‐PLAC8* and *GFP‐SPNS1* partially rescues or completely restores the defects in infection efficiency of the corresponding CRISPR KO cell lines (Fig 2D). Notably, we did not observe crossed complementation between the two genes (*GFP‐SPNS1* overexpression did not rescue infection on *PLAC8*‐KO cell line, or vice versa), nor did we detect higher levels of infection in cell lines transduced with control sgRNAs that overexpress the candidate genes. Next, to extend our results to another cellular model, we selected the second most susceptible cell line to S‐typed lentiviruses (Fig 1A), NCI‐H1299^ACE2^ (hereafter H1299^ACE2^), and analyzed the effect of loss‐of‐function of *PLAC8* and *SPNS1* as in Fig 2B. In agreement with our previous observations, *PLAC8‐* and *SPNS1*‐KO cell lines displayed substantially reduced infection efficiencies compared with cell lines carrying a control sgRNA, reaching levels similar to those of *ACE2*‐KO cells (Appendix Fig S1C), although the overall infection differences with respect to control cells were milder in this cellular model despite the efficient knockdown of *PLAC8* and *SPNS1* (Appendix Fig S1D).

Altogether, these experiments confirm and extend our screen results and demonstrate that loss‐of‐function of *PLAC8* and *SPNS1* specifically impairs viral entry of S‐typed lentiviruses in human lung cancer cells.

### PLAC8 and SPNS1 are key host factors for SARS‐CoV‐2 entry

**Figure 3:**
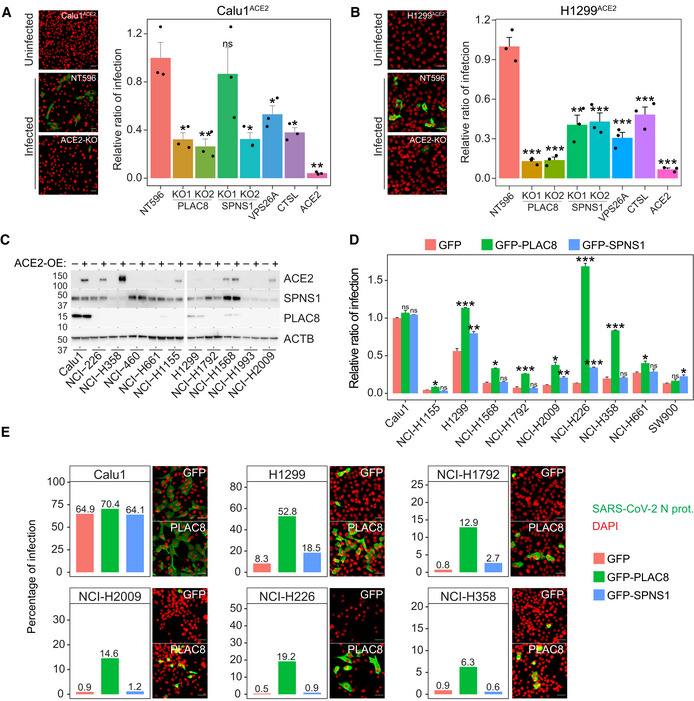
Validation of screen results using full SARS‐CoV‐2 infectious viruses A, BSusceptibility to infection with SARS‐CoV‐2 viruses in Calu1^ACE2^ (A) and H1299^ACE2^ (B) cell lines with loss‐of‐function of the indicated genes. Left: representative immunofluorescence images of SARS‐CoV‐2 nucleocapsid protein (green) and cell nuclei (red) in the indicated cell lines. Scale bar: 20 μm. Right: immunofluorescence quantification of the number of infected cells at 24 h postinfection in the different loss‐of‐function cell lines infected with SARS‐CoV‐2. Bars represent the average and SEM of the percentage of infected cells relative to CRISPR nontargeting transduced (NT596) cells in three biological replicates (three different CRISPR KO cell lines infected independently). The significance above each bar represents the one‐tail *t*‐test *P*‐value between each condition and CRISPR nontargeting transduced (NT596) cells.CWestern blot analyses of SPNS1 and PLAC8 protein levels in the different lung cancer cell lines tested in Fig 1 with or without *ACE2* overexpression. Note that PLAC8 and SPNS1 immunodetection was performed on the same blots as in Fig 1A and that ACE2 and Actin panels from Fig 1A were included here to allow comparison.DSusceptibility to Spike‐typed lentiviruses in the indicated *ACE2* overexpressing cancer cell lines that were transduced with *GFP*, *GFP‐PLAC8*, or *GFP‐SPNS1*. Bars represent the average and SEM of the percentage of infected cells in three technical replicates (independent infections of the same cell line) relative to Calu1^ACE2^‐GFP cells. The significance above each bar represents the *t*‐test between *GFP‐PLAC8* or *GFP‐SPNS1* and *GFP* cells for each cell line.ESusceptibility to infection with SARS‐CoV‐2 viruses in cell lines that overexpress *ACE2* and *GFP‐SPNS1* or *GFP‐PLAC8*. Bars represent the immunofluorescence quantification of the number of SARS‐CoV‐2 infected cells at 24 h postinfection. Representative images of SARS‐CoV‐2 nucleocapsid immunofluorescence (green) and cell nuclei (red) in *GFP* and *GFP‐PLAC8* cells are shown right on each plot. Scale bar: 20 μm. Data information: ns, *P*‐value ≥ 0.05, *P‐value < 0.05, **P‐value < 0.01, ***P‐value < 0.001. Source data are available online for this figure.

To evaluate the effect of *PLAC8* and *SPNS1* loss‐of‐function using full SARS‐CoV‐2 infectious virus, Calu1^ACE2^ KO cell lines were challenged with a SARS‐CoV‐2 inoculum and viral entry was assessed by immunofluorescence of nucleocapsid (N) protein 24 h postinfection (p.i.). As expected, *ACE2*‐KO had the strongest inhibitory effect, dropping the number of positive cells to almost zero (Fig 3A). Consistently with our previous results, loss‐of‐function of *PLAC8* and *SPNS1* caused a three‐fold reduction in the number of infected cells 24 h p.i. in at least one of the two KO cell lines tested, compared with control cells. Notably, the decrease in the number of positive cells was similar or even stronger than that obtained in the positive control cells *CTSL*‐KO and *VPS26A*‐KO, suggesting that PLAC8 and SPNS1 play an important role in SARS‐CoV‐2 entry. These results were confirmed using the H1299^ACE2^ model (Fig 3B). Remarkably, the effect of *PLAC8* loss‐of‐function was even greater in this cell line model, almost reaching the fold‐change levels of *ACE2*‐KO cells. *SPNS1* loss‐of‐function consistently resulted in a decrease of approximately 50% in the number of positive cells.

Preliminary experiments using *GFP‐PLAC8* and *GFP‐SPNS1* on Calu1^ACE2^ cells did not result in higher levels of infections (Fig 2D). We hypothesized that endogenous levels of these two proteins could be already too high to cause a significant change upon overexpression. To evaluate this possibility, we measured the levels of PLAC8 and SPNS1 on the lung cancer cell lines tested in Fig 1A. These two proteins showed very different patterns of expression across the panel of cell lines analyzed (Fig 3C). Thus, *SPNS1* was found ubiquitously expressed, with just a few lines showing lower‐than‐average levels. However, PLAC8 protein was almost undetectable in most cell lines, but very high levels were detected in Calu1 cells and to a lesser extent in H1299 and H1568 cells. Notably, Calu1^ACE2^ and H1299^ACE2^ cell lines showed the highest levels of infection with S‐typed lentiviruses (Fig 1A), while *ACE2* overexpression did not change protein levels of PLAC8 or SPNS1 in any cell line. Altogether, these results suggest that although both PLAC8 and SPNS1 are required for viral entry, PLAC8 might be a key limiting factor for SARS‐CoV‐2 infection. Accordingly, we decided to ectopically express *GFP‐PLAC8* and *GFP‐SPNS1* on 10 of the tested lung cancer cell lines that overexpress *ACE2* and evaluated their susceptibility to infection using our S‐typed lentiviral model. In this setting, *GFP‐PLAC8* overexpression boosted infection in almost all the cell lines tested, reaching up to a 20‐fold increase in the number of positive cells compared with the matching GFP expressing cells (Fig 3D). By contrast, Calu1^ACE2^ cells, which have already high endogenous levels of PLAC8, did not experience any increase in infection efficiency upon *PLAC8* overexpression. Notably, *SPNS1* overexpression did not have an overall impact on infection efficiency, showing only a modest increase in H226, H1299, H2009, and SW900 cells. This suggests that SPNS1 is a required but not sufficient host factor for SARS‐CoV‐2 entry. As expected, the percentage of infected cells using VSVG‐typed lentiviruses did not change in any cell line (Appendix Fig S2A). Overexpression levels were confirmed by recording FITC channel intensity during flow cytometer data acquisition (Appendix Fig S2B).

Prompted by these results, we proceeded to validate them using SARS‐CoV‐2 viruses. We selected the four cell lines that showed the strongest induction in infection efficiency upon *PLAC8* overexpression, as well as Calu1^ACE2^ and H1299 ^ACE2^ cells, and challenged them with SARS‐CoV‐2 for 24 h, after which cells were fixed and stained for protein N using immunofluorescence. Thus, Calu1^ACE2^ showed the highest infection efficiency compared with the other cell lines, whereas *GFP‐PLAC8* or *GFP‐SPNS1* overexpression did not increase it further (Fig 3E). By contrast, all the other cell lines displayed modest basal levels of infection, ranging from 2 to 20% positive cells, that were boosted dramatically upon *PLAC8* overexpression (up to 38‐fold change compared with *GFP* overexpression for H226^ACE2^ cells). Likewise, the results for *GFP‐SPNS1* overexpression recapitulated the previous observations with lentiviruses, producing only a moderate increase in the number of positive cells in few cell lines (H1792, H2009, and H226). Finally, given the reported importance of *TMPRSS2* in SARS‐CoV‐2 entry (Koch *et al*, 2021: 2), we measured the levels of this protein in several cell lines and confirmed that all our cellular models have detectable levels of TMPRSS2 that do not correlate with their infection efficiency (Appendix Fig S2C).

### PLAC8 is highly expressed in SARS‐CoV‐2 target cells

**Figure 4:**
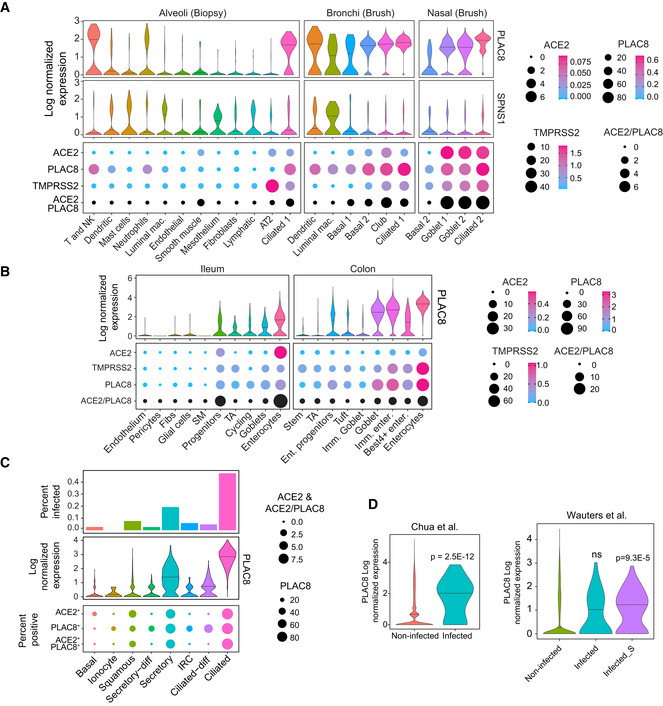
*PLAC8* and *SPNS1* gene expression studies *PLAC8* and *SPNS1* gene expression levels in scRNA‐Seq data of lung tissues from different locations (Vieira Braga *et al*, 2019). Top plot: violin plots of *PLAC8* and *SPNS1* expression levels in the different cell types. The horizontal bar represents the median. Bottom plot: dot plot showing the percentage of cells in each population with detectable gene expression for the indicated genes. The size of the dot indicates the percentage of positive cells, while the color indicates the mean expression levels in each population.A similar analysis to (A) in scRNA‐Seq data from human gut tissues (Martin *et al*, 2019; Smillie *et al*, 2019).
*PLAC8* and *ACE2* expression analyses in scRNA‐Seq data from nasopharyngeal samples from healthy donors and COVID‐19 patients (Chua *et al*, 2020). Top plot: bar plot showing the percentage of cells infected with SARS‐CoV‐2 in each population in samples from COVID‐19 patients. Middle plot: violin plot of *PLAC8* expression levels in each cell population in samples from healthy donors. The horizontal bar represents the median. Bottom plot: dot plot showing the co‐expression levels of *PLAC8* and *ACE2* in samples from healthy donors. The size of the dots indicates the percentage of positive cells for each gene and population.Violin plots showing the *PLAC8* differential expression between infected and noninfected cells from lung samples of COVID‐19 patients from two different studies (Chua *et al*, 2020; Wauters *et al*, 2021). The horizontal bar represents the median. Note that in Wauters *et al* (2021), infected cells are defined by the presence of any SARS‐CoV‐2 read, while infected_S are bona fide infected cells that also present reads aligning to Spike gene. ns, nonsignificant. *P, two‐sample Wilcoxon test *P*‐value.

Accumulated evidence from different studies points at ciliated and secretory cells from the upper respiratory tract as the most susceptible cell types to SARS‐CoV‐2 infection (Chua *et al*, 2020; Lee *et al*, 2020; Lukassen *et al*, 2020; Sungnak *et al*, 2020). To further explore the relationship between *PLAC8* and *SPNS1* and SARS‐CoV‐2 infection, we analyzed the expression levels of these genes in scRNA‐Seq data from different lung locations provided by Vieira *et al* (Vieira Braga *et al*, 2019; Sungnak *et al*, 2020). Strikingly, we found that *PLAC8* is highly expressed in the epithelial cell lineages from different lung locations and shows the highest expression levels in ciliated and secretory cells (goblet and club) from the bronchi and nasal cavity (Fig 4A). By contrast, *SPNS1* shows overall low levels of expression in lung epithelial cell types and it is not particularly enriched in any cell population, consistently with our previous observations in different lung cancer cell lines and its putative role as a ubiquitous nonlimiting host factor. Given these results, we decided to explore the co‐expression of PLAC8 and the host factors ACE2 and TMPRSS2. As reported by Sungnak *et al* (2020) the percentage of ACE2‐positive (ACE2^+^) cells is generally very low in lung tissues, but secretory (goblet) and ciliated cell populations from the nasal cavity display the highest percentage of ACE2 and TMPRSS2 double‐positive cells (Sungnak *et al*, 2020). In line with this report, these cell populations also show the highest percentage of ACE2 and PLAC8 double‐positive cells, with virtually all ACE2^+^ cells showing PLAC8 expression (Fig 4A). To reinforce these results, we analyzed an independent scRNA‐Seq dataset from lung biopsies (Deprez *et al*, 2020) and obtained very similar results: *PLAC8* is highly expressed in ciliated and secretory cells and these populations show the highest percentage of double‐positive cells for *ACE2* and *PLAC8* transcripts (Appendix Fig S3).

Although respiratory symptoms dominate the clinical features of COVID‐19 patients, 15% of these patients manifest gastrointestinal symptoms and increasing evidence suggests that the gastrointestinal tract might also be affected by SARS‐CoV‐2 (Mao *et al*, 2020). Thus, enterocytes from the small intestine show the highest *ACE2* expression levels in the human body, and studies using organoids confirmed that they are readily infected by SARS‐CoV‐2 (Lamers *et al*, 2020; Sungnak *et al*, 2020). To assess whether *PLAC8* might be also an important host factor for gut SARS‐CoV‐2 infection, we analyzed its expression profile in scRNA‐Seq data from ileum and colon samples (Martin *et al*, 2019; Smillie *et al*, 2019; Sungnak *et al*, 2020). Notably, we found that enterocytes from both locations show very high levels of *PLAC8* expression, followed by goblet secretory cells (Fig 4B). Moreover, co‐expression analyses demonstrate that these cell types display a high percentage of *ACE2* and *PLAC8* double‐positive cells, and they also express *TMPRSS2*, therefore representing a potentially high susceptible cell type for SARS‐CoV‐2 infection.

Finally, to further reinforce the link between PLAC8 and SARS‐CoV‐2 infection, we explored its expression levels in nasopharyngeal samples from COVID‐19 patients (Chua *et al*, 2020). As reported by Chua *et al* (2020), ciliated and to a lesser extent, secretory cells contain the highest percentage of SARS‐CoV‐2 infected cells. Consistent with the putative role of *PLAC8* in viral infection, we observed that these two cell types also show the highest *PLAC8* expression levels and a high percentage of *ACE2* and *PLAC8* double‐positive cells (Fig 4C). Moreover, when assessed globally, *PLAC8* was highly overexpressed in SARS‐CoV‐2 infected cells compared with uninfected cells (Fig 4D). To further confirm these observations, we analyzed an independent scRNA‐Seq dataset of lung samples from COVID‐19 patients (Wauters *et al*, 2021) and consistently found that *PLAC8* is significantly overexpressed in *bona fide* SARS‐CoV‐2 infected cells (COVID19_Infected_S) compared with uninfected cells from COVID‐19 patients (Fig 4D).

### PLAC8 and SPNS1 colocalize with ACE2 and spike protein

We then set out to elucidate the molecular mechanisms underlying the role of PLAC8 and SPNS1 in SARS‐CoV‐2 infection. A possible explanation for our observations could be a hypothetical effect of the loss‐of‐function of these genes on ACE2 protein levels. However, immunoblot analyses in Calu1^ACE2^ cells did not reveal significant differences in total ACE2 protein levels between *PLAC8*‐KO or *SPNS1*‐KO cells compared with control cells (Appendix Fig S4A). Likewise, similar levels of ACE2 were observed in the different cell lines that overexpress *GFP‐PLAC8* and *GFP‐SPNS1* (Appendix Fig S4B), ruling out the possibility that the differences in the viral entry are caused by differences in ACE2 protein levels. Even though total ACE2 levels remain unchanged, its surface localization –the fraction available for viral recognition– might be affected by an imbalance between its transport to the cell membrane and its internalization and recycling. To test this hypothesis, we performed surface immunostaining of ACE2 coupled to flow cytometry to quantitatively measure the cell membrane fraction of ACE2 in our Calu1^ACE2^ model. This experiment showed that, while ACE2 surface levels were decreased in *ACE2*‐KO cells, *PLAC8*‐KO and *SPNS1*‐KO cells displayed similar levels of the viral receptor (Appendix Fig S4C).

**Figure 5:**
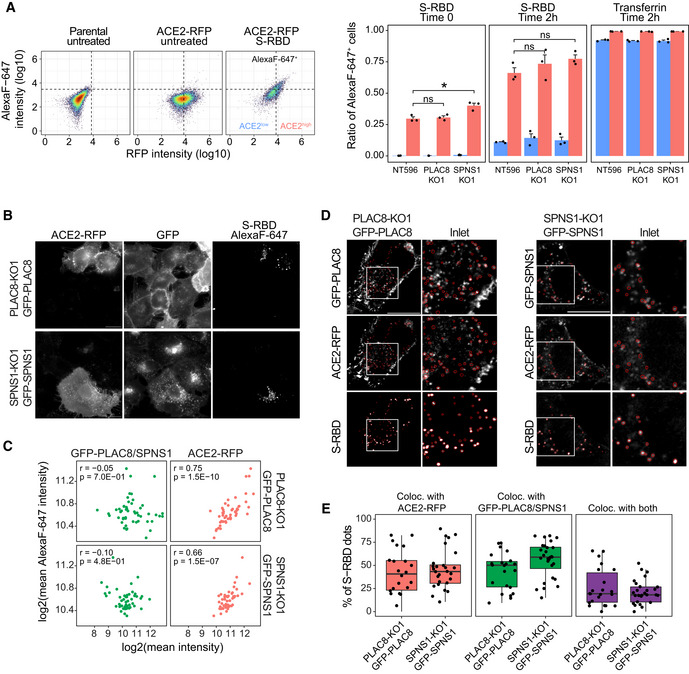
PLAC8 and SPNS1 do not affect binding and endocytosis ABinding and endocytosis studies with Alexa Fluor 647‐conjugated S‐RBD or transferrin in Calu1‐ACE2‐RFP cells deficient in *PLAC8* and *SPNS1*. Left: scatterplots of flow cytometry data showing the cutoffs used to define ACE2‐RFP‐ and Alexa Fluor 647‐positive cells. Right: bar plots showing the ratio of cells positive for Alexa Fluor 647 in either ACE2‐RFP‐positive (red) or ‐negative (blue) cells in binding and endocytosis experiments with S‐RBD or transferrin. Bars represent the average and SEM of three biological replicates (three different CRISPR KO cell lines). The significance above each bar represents the *t*‐test *P*‐value between each condition and CRISPR nontargeting transduced (NT596) cells. ns, *P*‐value ≥ 0.05, *P‐value < 0.05, **P‐value < 0.01, ***P‐value < 0.001.B–EEndocytosis experiments with S‐RBD in *PLAC8‐* or *SPNS1‐KO* Calu1‐ACE2‐RFP cells with overexpression of GFP‐PLAC8 or GFP‐SPNS1, respectively. Cells were incubated with S‐RBD for 1 h at 37°C and fixed with 4% p‐formaldehyde after an acid wash to remove noninternalized S‐RBD. Representative wide‐field fluorescence images (B) and quantification of mean fluorescence intensities of each fluorophore (C) in each cell show that the S‐RBD signal correlates with ACE2‐RFP expression but not with GFP‐PLAC8 or GFP‐SPNS1. The Pearson correlation coefficient (*r*) and correlation test *P*‐value (*P*) is indicated within each scatterplot. Each dot represents a cell measurement from different microphotographs of a single replicate. Scale bar: 20 μm. Representative fluorescence confocal microphotographs (D) and spot colocalization analyses using *ComDet* plugin (E) demonstrate colocalization of S‐RBD endosomes with ACE2‐RFP and GFP‐PLAC8 or GFP‐SPNS1. (D) Red ovals defining the detected S‐RBD dots are overlaid in each channel to assess the colocalization with ACE2‐RFP, GFP‐PLAC8, or GFP‐SPNS1. Inlets show a magnification of the region defined by the white squares. Scale bar: 20 μm. (E) Boxplots representing the percentage of internalized S‐RBD that colocalize with ACE2‐RFP, GFP‐PLAC8, GFP‐SPNS1 or their combination (ACE2‐RFP and GFP‐PLAC8 or ACE2‐RFP and GFP‐SPNS1). The central band represents the median, the box represents the interquartile range (IQR) between the 25^th^ and 75^th^ percentile, and the whiskers extend from the box to 1.5*IQR. Each dot represents a cell measurement from different microphotographs of a single replicate. Source data are available online for this figure.

Given these results, we next focused on the binding and endocytosis of SARS‐CoV‐2 Spike protein upon loss of function of *PLAC8* and *SPNS1*. To track ACE2 levels, we generated new PLAC8‐KO and SPNS1‐KO Calu1 cell lines with stable overexpression of ACE2 fused to the fluorescence protein tagRFP (hereafter ACE2‐RFP). Cells were incubated at 4°C for 1 h with the receptor binding domain of SARS‐CoV‐2 Spike protein (hereafter S‐RBD) conjugated with the fluorophore Alexa Fluor 647 to measure binding, and then, the temperature was raised to 37°C for 2 h to allow endocytosis. Flow cytometry analysis of these cell populations did not reveal a reduction in S‐RBD binding in Calu1‐ACE2‐RFP cells with loss‐of‐function of *PLAC8* or *SPNS1*, compared with nontargeting CRISPR control cells (Fig 5A). Notably, the same results were obtained in the endocytosis experiments. However, we observed in all samples the expected correlation between ACE2‐RFP levels and S‐RBD binding and endocytosis. Parallel experiments using Alexa Fluor 647‐conjugated transferrin did not show endocytosis differences between cells with low and high levels of ACE2‐RFP, as expected. Likewise, *PLAC8* and *SPNS1* loss‐of‐function did not affect transferrin endocytosis. Altogether, these results indicate that PLAC8 and SPNS1 do not affect the surface levels of ACE2 nor its ability to bind and internalize SARS‐CoV‐2 Spike protein.

To get more insights into the molecular role of PLAC8 and SPNS1 in SARS‐CoV‐2 entry, we overexpressed GFP fusions of these proteins in Calu1 ACE2‐RFP cells and used wide‐field live‐cell imaging to study their subcellular localization. This experiment revealed a remarkable colocalization between ACE2‐RFP and GFP‐PLAC8, being both proteins predominantly located at the cell surface but also in intracellular vesicles (Appendix Fig S5A). It is worth noting the abundance of these two proteins in filamentous projections that resemble filopodia. Interestingly, SPNS1 is mainly distributed as intracellular puncta—many of them sharing ACE2‐RFP localization—but a closer inspection revealed also a plasma membrane localization, including filopodia. Next, we used these cellular models to study the endocytosis of S‐RBD. To avoid competition, we knocked out endogenous *PLAC8* and *SPNS1* using CRISPR‐Cas9 guides that do not target exogenous GFP‐PLAC8 and GFP‐SPNS1 (Appendix Fig S5B). First, wide‐field microscopy was used to quantify the internalization of S‐RBD (Fig 5B). We took advantage of the polyclonal nature of our cellular models to correlate the mean intensity values of ACE2‐RFP, S‐RBD, and GFP‐PLAC8 or GFP‐SPNS1. Consistently with our previous flow cytometry experiments, S‐RBD internalization was not affected by the levels of GFP‐PLAC8 or GFP‐SPNS1, but it correlated strongly with ACE2‐RFP expression (Fig 5C). Next, we used confocal imaging to study the colocalization of ACE2, PLAC8, and SPNS1 with endocytosed S‐RBD. As can be seen in Fig 5D and E, a median of 42% and 56% of internalized S‐RBD dots colocalized with PLAC8 and SPNS1 dots. Likewise, the ACE2‐RFP signal was present in 42% and 44% of S‐RBD dots in GFP‐PLAC8 and GFP‐SPNS1 samples, respectively, although these measurements might be underestimated due to the overall low expression levels of ACE2‐RFP and/or the fluorophore brightness. Nonetheless, we observed a median of 25% and 20% S‐RBD dots colocalizing both with ACE2‐RFP and GFP‐PLAC8 or GFP‐SPNS1, respectively. Finally, given the predominant localization of PLAC8 in the plasma membrane, we also examined whether there is a physical interaction with ACE2 or SARS‐CoV‐2 Spike. To test this, protein extracts from Calu1 cells overexpressing ACE2‐GFP were incubated with 6xHis‐tagged full‐length SARS‐CoV‐2 Spike protein and subjected to immunoprecipitation with an anti‐GFP antibody (Appendix Fig S5C). This experiment showed that, while Spike protein is effectively co‐immunoprecipitated together with ACE2‐GFP, no endogenous PLAC8 was detected in the immunoprecipitated material. Likewise, immunoprecipitation of GFP‐PLAC8 in similar experiments with *PLAC8‐KO* Calu1^ACE2^ cells overexpressing GFP‐PLAC8 failed to retrieve ACE2 or recombinant Spike (Appendix Fig S5D).

Altogether, these experiments rule out that PLAC8 and SPNS1 affect SARS‐CoV‐2 infection through the modulation of ligand‐mediated endocytosis of ACE2, and instead suggest that these two proteins participate in later stages of viral infection, such as vesicle trafficking or membrane fusion and genome release.

### PLAC8 and SPNS1 deficiency increases autophagy

**Figure 6:**
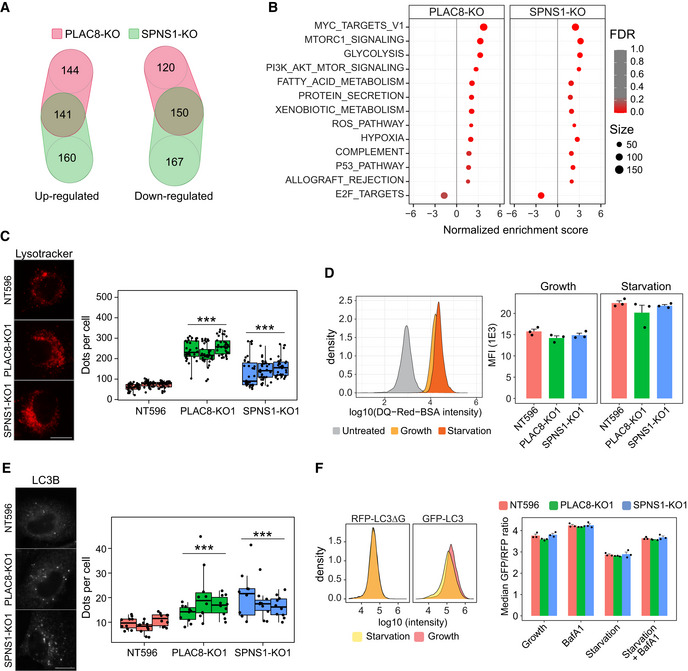
*PLAC8* and *SPNS1* deficiency increase autophagic activity Venn diagrams showing the overlap of significantly up‐ or downregulated proteins in mass spectrometry studies between *PLAC8‐* and *SPNS1‐KO* Calu1^ACE2^ cells, compared with CRISPR nontargeting control cells (NT596).Dot plot summarizing the results of gene set enrichment analyses (GSEA) in *PLAC8‐* and *SPNS1‐KO* Calu1^ACE2^ cells compared with CRISPR nontargeting control cells (NT596). The size of the dots indicates the number of proteins in each category, while the color scale indicates the false discovery rate (FDR) values.Representative images (left) and quantification (right) of the number of acidic particles in *PLAC8‐* and *SPNS1‐KO* Calu1^ACE2^ cells compared with CRISPR nontargeting control cells (NT596) using live‐cell lysotracker staining. The two samples Wilcoxon Test *P*‐value of the pooled observations compared with NT596 cells is indicated above each condition. The central band represents the median, the box represents the interquartile range (IQR) between the 25^th^ and 75^th^ percentile, and the whiskers extend from the box to 1.5*IQR. Each dot represents a cell measurement from different microphotographs of a single replicate. Three biological replicates per condition were used. Scale bar: 20 μm.Lysosomal degradation studies in Calu1^ACE2^ cells using the fluorogenic substrate DQ‐Red‐BSA. Left: density plots representing the shift in fluorescence intensity in NT596 cells untreated or treated with DQ‐Red‐BSA under growth or serum starvation conditions. Right: bar plot representing the mean and SEM of the median fluorescence intensity values (MFI) of three biological replicates of *PLAC8‐* and *SPNS1‐KO* and CRISPR nontargeting cells in growth and serum starvation conditions.Representative images (left) and quantification (right) of the number of LC3B particles in *PLAC8‐* and *SPNS1‐KO* Calu1^ACE2^ cells compared with CRISPR nontargeting control cells (NT596) using LC3B immunofluorescence staining. The two samples Wilcoxon Test *P*‐value of the pooled observations compared with NT596 cells is indicated above each condition. The central band represents the median, the box represents the interquartile range (IQR) between the 25^th^ and 75^th^ percentile, and the whiskers extend from the box to 1.5*IQR. Each dot represents a cell measurement from different microphotographs of a single replicate. Three biological replicates per condition were used. Scale bar: 20 μm.Autophagic flux analyses using the fluorescence reporter GFP‐LC3‐RFP‐LC3ΔG in *PLAC8‐* and *SPNS1‐KO* Calu1^ACE2^ cells compared with CRISPR nontargeting control cells (NT596). Left: density plots showing the shift in GFP intensity under starvation conditions compared with control growth conditions in NT596 Calu1^ACE2^ cells. Right: bar plots showing the mean and SEM of the median GFP/RFP intensity ratio of three biological replicates in the different cell lines and conditions. Source data are available online for this figure.

To get more insights into the molecular mechanisms behind our observations, we performed high‐throughput quantitative proteomics of Calu1^ACE2^ cells with loss‐of‐function of PLAC8 and SPNS1. These experiments revealed profound changes in the levels of numerous proteins. Thus, we identified 285 and 275 significantly upregulated proteins and 301 and 317 significantly downregulated proteins in the proteomes of *PLAC8*‐KO and *SPNS1*‐KO cells, respectively, compared with control cells (adjusted *P*‐value < 0.05 and ¦Zq_difference¦ > 0.5) (Appendix Fig S6A; Dataset EV2). Remarkably, comparative analyses showed a strong correlation between the protein changes in *PLAC8‐*KO and *SPNS1‐*KO cells and a high degree of overlap in the sets of significantly changed proteins (Fig 6A; Appendix Fig S6B), suggesting that both host factors share cellular functions. In line with these observations, gene set enrichment analyses (GSEA) revealed a shared molecular signature that points to an overexpression of mTOR signaling pathway factors (Fig 6B; Appendix Fig S6C). Thus, *mTORC1* and *PI3K/AKT/mTOR* molecular signatures are among the top enriched pathways in both *PLAC8*‐KO and *SPNS1*‐KO samples. We also detected a shared concomitant enrichment in processes regulated by this pathway, such as glycolysis, fatty acid metabolism, and protein secretion.

PLAC8 and SPNS1 have been associated with autophagolysosomal regulation in different cell types (Dermaut *et al*, 2005; Rong *et al*, 2011; Kinsey *et al*, 2014; Sasaki *et al*, 2014; Mingo *et al*, 2015; Segawa *et al*, 2018; Feng *et al*, 2021). To study whether PLAC8 and SPNS1 loss‐of‐function has an impact on this process, we first carried out colocalization studies in Calu1^ACE2^ cells using the lysosomal marker lysotracker. This analysis showed a high degree of overlap between intracellular GFP‐SPNS1 and the lysosomal marker lysotracker (Appendix Fig S7A). Similarly, we observed partial colocalization of intracellular GFP‐PLAC8 and lysotracker in Calu1^ACE2^ cells, although GFP signal was also detected in nonacidic punctuated structures. We then proceeded to study the lysosomal compartment of *PLAC8‐* and *SPNS1‐KO* Calu1^ACE2^ cells. Live‐cell lysotracker staining revealed that these cells present a marked increase in the number of lysosomes, both in growth and serum starvation conditions, compared with CRISPR nontargeting control cells (Fig 6C; Appendix Fig S7B), a result that was independently confirmed by immunofluorescence detection of the lysosomal marker LAMP1 (Appendix Fig S7C). Furthermore, studies using lysosensor revealed that the expansion of the lysosomal compartment is also accompanied by strong acidification of their lumen (Appendix Fig S7D). Strikingly, experiments with DQ‐BSA‐Red yielded no differences in Calu1^ACE2^ *PLAC8‐* and *SPNS1‐KO* cells compared with CRISPR nontargeting cells, meaning that neither lysosomal degradation nor trafficking of endosomes is altered by the loss‐of‐function of these proteins (Fig 6D). Given these results, we next performed a detailed analysis of the autophagic activity in these cells. Immunofluorescence analyses of the autophagosome markers LC3B and SQSTM1/p62 showed a remarkable accumulation in the number of these structures in *PLAC8‐* and *SPNS1‐KO* Calu1^ACE2^ cells compared with CRISPR nontargeting control cells (Fig 6E; Appendix Fig S8A and B). Notably, autophagic flux analyses through the use of the lysosomal inhibitor Bafilomycin A1 (BafA1; Klionsky *et al*, 2021) confirmed that the increase in autophagosome markers is conserved during starvation or when lysosomal degradation is inhibited, suggesting that *PLAC8* and *SPNS1* loss‐of‐function increase both the formation and degradation of autophagosomes, ruling out the possibility of a block in autophagic degradation. To independently confirm these results, we transduced the GFP‐LC3‐RFP‐LC3ΔG reporter (Kaizuka *et al*, 2016) in Calu1^ACE2^ cells with loss‐of‐function of *PLAC8* and *SPNS1* and measured autophagic flux in starvation and BafA1 conditions (Fig 6F). Consistently with the above observations, no block in autophagic flux was observed in *PLAC8‐* and *SPNS1‐KO* cells compared with the CRISPR nontargeting control.

Finally, we reasoned that if the autophagolysosomal expansion underlies the defects in SARS‐CoV‐2 infection, the opposite alteration should be observed in cell lines that experience an increase in SARS‐CoV‐2 infection upon overexpression of PLAC8 and SPNS1. Thus, we next studied this compartment in the two cell lines with the highest increase in SARS‐CoV‐2 infection efficiency upon overexpression of PLAC8: H226 and H358. However, in contrast to *PLAC8* and *SPNS1* loss‐of‐function in Calu1 cells, overexpression of GFP‐PLAC8 or GFP‐SPNS1 in H226^ACE2^ or H358^ACE2^ did not impact the number of lysosomes or autophagosomes in any condition (Appendix Fig S9A and B), suggesting that the role of PLAC8 and SPNS1 on SARS‐CoV‐2 infection occurs through a different mechanism that is molecularly connected to autophagolysosomal regulation.

## Discussion

In this work, we have performed a genome‐wide CRISPR knockout screen to find host factors that participate in SARS‐CoV‐2 infection. First, we used a simplified model consisting of lentiviruses pseudotyped with the Spike protein of this coronavirus, and then, we validated the results of the screening by using full SARS‐CoV‐2 virus in a high‐biosecurity facility. This approach allows us to specifically dissect the viral entry interactions with the host proteins and reduces the biosafety requirements by not using replicative viruses during the first steps of the experimental work. As a cellular model, we selected the human lung carcinoma cell line Calu1, since it showed the highest infection efficiency among a panel of different lung cancer epithelial cell lines. The performance of our screen was validated by the significant degree of overlap between our hits and those reported by other laboratories that utilized full SARS‐CoV‐2 viruses (Daniloski *et al*, 2021; Zhu *et al*, 2021). Thus, *ACE2* and *CTSL* are our top‐ranked hits, and we have confirmed a large enrichment in genes encoding proteins that participate in endosome traffic. Nevertheless, our screen largely extends the findings described in these previous studies by identifying novel host factors that belong to these and other molecular pathways. Moreover, since our model system only addresses viral entry, our results help dissecting the role of host factors identified in those screens that have used full SARS‐CoV‐2 viruses. For example, none of the 13 vacuolar‐ATPase proton pumps reported by Daniloski *et al* (2021) are present among our significant hits, suggesting that they participate in other aspects of the viral life cycle, such as assembly or regression. However, we found other genes related to lysosomal function and its interplay with autophagy. The most prominent ones, besides *CTSL*, were *PLAC8* and *SPNS1*, which ranked 3^rd^ and 4^th^ in our hit list. *SPNS1* encodes a transmembrane protein that localizes to the lysosome, which functions as an efflux transporter that regulates lysosome homeostasis and mTOR activation (Nakano, 2019). *PLAC8*, which has not been reported in any previous screen for SARS‐CoV‐2, was originally identified as a highly expressed gene in the human placenta and encodes a small transmembrane protein linked to phagolysosome fusion and autophagy regulation (Feng *et al*, 2021). It is also remarkable our finding of *TYK2* (tyrosine kinase 2) as a host factor whose loss‐of‐function favors the SARS‐CoV‐2 infection. This gene encodes a protein kinase recently linked to severe COVID‐19 disease course (COVID‐19 Host Genetics Initiative, 2021). However, we cannot rule out that the finding of *TYK2* as a proviral factor in our SARS‐CoV‐2 screen may result from a cellular response against the pseudotyped lentivirus used in the study. It is also very relevant that PLSCR1, a phospholipid scramblase reported to inhibit PLAC8 (Li *et al*, 2006), ranks 5^th^ in the list of enriched knocked‐out genes in our screen (FDR of 0.06).

Our finding of novel proviral and antiviral factors reflects the complexity of the SARS‐CoV‐2 viral life cycle and suggests that additional genes implicated in the different steps of infection remain to be discovered. The absence of *PLAC8*, *SPNS1*, or *TYK2*, which are at the top of our candidate host factors for SARS‐CoV‐2 infection, in previous screens likely derives from differences between cell types, CRISPR libraries, virus isolates, and experimental conditions used by the different groups. In this sense, our selected cellular model (Calu1) turned out to be one of the few cell lines with high levels of PLAC8 among a panel of lung cancer cells. Actually, most previous screens have used human liver or kidney cells, or Vero E6 kidney cells from African green monkeys, which appear to be less suitable than the human pulmonary cells (the primary SARS‐CoV‐2 infection target) used in our work, as well as in those screens showing a substantial overlap with our top‐ranked genes (Daniloski *et al*, 2021; Zhu *et al*, 2021). Nevertheless, beyond these cell line‐specific discrepancies among hits identified in different screens and considering both the dependency of SARS‐CoV‐2 on CTSL‐mediated lysosomal activation of Spike protein for membrane fusion and the very high rank of lysosomal genes *PLAC8* and *SPNS1* in our screen, we decided to perform follow‐up studies of these two genes. In these studies, loss‐of‐function of *SPNS1* and *PLAC8* using different CRISPR vectors significantly reduced the infection susceptibility of Calu1^ACE2^ cells to S‐typed lentiviruses. Rescue experiments on these cells overproducing GFP‐SPNS1 and GFP‐PLAC8 proteins confirmed the specificity of our observations, while similar experiments on H1299^ACE2^ cells reinforced and generalized our results. Importantly, we did not observe changes in infectability when using VSVG‐pseudotyped lentiviruses, which demonstrates that the observed phenotype arises from the interplay between viral Spike and the host factors SPNS1 and PLAC8. Likewise, this observation reinforces the role of lysosomes in SARS‐CoV‐2 entry, since in contrast to Spike, VSVG activation occurs rapidly upon endocytosis at the less acidic early endosome (Johannsdottir *et al*, 2009).

We have also validated our results using full SARS‐CoV‐2 infectious virus both in Calu1^ACE2^ and H1299^ACE2^ cells, where we found that *PLAC8* and *SPNS1* loss‐of‐function fully replicated our observations using pseudotyped lentiviral particles. Interestingly, when we analyzed the levels of these factors in different lung cancer cell lines, we noted that while SPNS1 is uniformly expressed, PLAC8 was barely detectable in most cell lines, but its expression was high in those cell lines showing the greatest infection susceptibility to S‐typed lentiviruses, suggesting that PLAC8 might be a limiting factor. In fact, the infection efficiency correlated much better with PLAC8 than with ACE2 levels, since, for example, H358 cells, which have the highest ACE2 levels but no detectable PLAC8, display much lower infection efficiencies than H1299^ACE2^ cells, which exhibit low ACE2 and high PLAC8 levels. In line with this hypothesis, when we overexpressed *PLAC8*, we observed increased susceptibility to S‐typed lentiviruses in 8 out of 10 cell lines tested. Notably, Calu1^ACE2^, the cell line that expresses the highest *PLAC8* levels, did not experience any infection change, while H1299^ACE2^ cells, which exhibits moderate levels of this protein, showed only a modest increase in infectability upon *PLAC8* overexpression.

Importantly, these results were fully recapitulated using full SARS‐CoV‐2 viruses. Of special interest are the results with H226^ACE2^ cells, where *PLAC8* overexpression caused more than a 10‐fold increase in infectability by S‐typed lentiviruses and SARS‐CoV‐2 viruses. Consistent with these results, our analysis of publicly available scRNA‐Seq datasets showed that *PLAC8* is highly expressed in the most SARS‐CoV‐2 susceptible epithelial cell types –ciliated and secretory– from the upper and lower respiratory tract, where we observed a strong co‐expression of genes encoding the viral receptor *ACE2* and the activating protease *TMPRSS2*. We also found a much higher expression of *PLAC8* in virus‐infected cells derived from COVID‐19 patients than in uninfected cells from the same patients. The reported discovery that *PLAC8* overexpression is a fundamental component of a molecular signature found in community‐acquired pneumonia patients provides an independent validation to our proposal that this factor plays a significant role in pulmonary pathology (Scicluna *et al*, 2015). Strikingly, we have also found that enterocytes from the intestine and colon tissues that are frequently affected by SARS‐CoV‐2 infection‐ show extremely high levels of *PLAC8* expression and display the highest percentage of *ACE2* and *TMPRSS2* co‐expression. In line with these observations, zebrafish PLAC8 is localized at the apical domain of differentiated gut epithelium and the base of cilia in different epithelial structures (Ma, 2013). It is noteworthy that, similarly to enterocytes, human ciliated respiratory cells also present microvilli, and recent work has proposed that ACE2 and TMPRSS2 localize to these structures and that SARS‐CoV‐2 extracellular virions are predominantly in contact with them (Pinto *et al*, 2022).

Mechanistically, we have demonstrated that loss‐of‐function of *PLAC8* and *SPNS1* do not affect the surface availability of the ACE2 viral receptor nor its ability to bind recombinant Spike RBD. Likewise, cells deficient in *PLAC8* or *SPNS1* did not show defects in recombinant Spike protein internalization. However, our confocal microscopy analyses indicate that both PLAC8 and SPNS1 localize to the plasma membrane and acidic vesicles of the late endosome and lysosomal compartments, and we were able to demonstrate that internalized S‐RBD particles colocalize with GFP‐PLAC8 and GFP‐SPNS1‐positive vesicles in the cytoplasm. However, immunoprecipitation studies do not point at a direct physical interaction of PLAC8 and SPNS1 with ACE2 or Spike proteins, but, being all membrane‐associated proteins, we cannot rule out weak or context‐specific interactions that are not captured in these experiments. All these pieces of evidence indicate that the interplay between these novel host factors and SARS‐CoV‐2 occurs intracellularly. On the other hand, our high‐throughput quantitative proteomics of Calu1^ACE2^ cells has revealed an mTOR‐related signaling signature in both *SPNS1*‐ and *PLAC8*‐KO cells, suggesting that these host factors somehow share molecular functions. Related to this observation, PLAC8‐ and SPNS1‐deficient cells seem to have increased autophagic activity, based on the accumulation of autophagosomes and the absence of blockage in our autophagic flux assays. In line with this, live‐cell lysotracker and lysosensor staining and LAMP1 immunofluorescence revealed an expansion and acidification of the late endosome and lysosomal compartments in PLAC8‐ and SPNS1‐deficient cells. Based on these observations, SPNS1 may function as a required factor whose loss‐of‐function blocks viral entry by inhibiting Spike activation and viral membrane fusion in lysosomes. Consistently with this proposal, several works have reported enlargement of late endosome and lysosomal compartments, as well as defects in endosome‐to‐lysosome trafficking and autophagic activity in *SPNS1* loss‐of‐function models (Sweeney & Davis, 2002; Young *et al*, 2002; Dermaut *et al*, 2005; Rong *et al*, 2011; Yuva‐Aydemir *et al*, 2011; Sasaki *et al*, 2014). In line with this model, overexpression of SPNS1 had mild or no effect on SARS‐CoV‐2 infection efficiency in all cell lines tested. By contrast, we did not observe alterations in the uptake and lysosomal degradation of fluorogenic BSA, but we cannot rule out that specific proteolytic activities against Spike proteins are altered by lysosomal acidification. On the other hand, the broad subcellular localization of PLAC8 suggests its putative ability to participate in different aspects of SARS‐CoV‐2 intracellular trafficking and release. However, the proposed role for PLAC8 in autophagic flux regulation by enhancing phagolysosome fusion (Kinsey *et al*, 2014; Huang *et al*, 2020) would agree with a role for this protein in the fusion of endocytic vesicles containing SARS‐CoV‐2 virions with lysosomes. Our finding that infection with VSVG‐typed lentiviruses, which release their genome in the early endosome, is not affected by *PLAC8* loss‐of‐function would support this hypothesis. Strikingly, we do not see alterations in the autophagolysosomal compartment of cells that experience an increased SARS‐CoV‐2 infection efficiency upon overexpression of GFP‐PLAC8, suggesting that, although PLAC8 is molecularly connected to autophagy and lysosomal homeostasis, the autophagolysosomal expansion of PLAC8‐KO cells is not the primary cause of their infection defects. Given its small size and its wide subcellular distribution, PLAC8 may also function as an adaptor protein that regulates vesicle trafficking through interaction with viral Spike protein or ACE2. In fact, our screen and those from others have identified numerous members of vesicle trafficking complexes as important host factors for SARS‐CoV‐2 infection (Fig 1D). Likewise, a recent work has reported that the SNX27‐retromer complex inhibits SARS‐CoV‐2 entry by redirecting the endocytosed particles to recycling endosomes instead of the late endosome/lysosome pathway (Yang *et al*, 2022). Similarly, it has been shown that deletion of the retromer component VPS29 inhibits SARS‐CoV‐2 infection and results in entrapment of VSV/SARS‐CoV‐2 chimeric viruses in the endosomes and the loss of endosomal cathepsin activity (Poston *et al*, 2022). It is worth noting that, during the final round of revision of this work, another genome‐wide CRISPR screen has identified PLAC8 as an essential factor for swine acute diarrhea syndrome coronavirus (SADS‐CoV), an alpha‐CoV‐1 virus closely related to beta‐CoVs (Tse *et al*, 2022). Interestingly, they also concluded that most likely PLAC8 participates in vesicle trafficking in the early stages of the SADS‐CoV life cycle, but it does not affect virion binding or endocytosis. This work reinforces our findings and supports the potential of PLAC8 as a pan‐CoV therapeutic target. Nevertheless, further experimental work will be necessary to clarify all the proposed roles for SPNS1 and PLAC8 in the context of CoV infections.

The autophagy machinery has been linked with viral replication and egression in multiple works, but very little is known about the interplay between this pathway and SARS‐CoV‐2 entry (He *et al*, 2022; Lan *et al*, 2022). However, there are evidences supporting that lysosomotropic agents that disrupt the autophagy‐lysosome pathway, such as azithromycin or chloroquine/hydroxychloroquine, are promising drugs to block SARS‐CoV‐2 entry (Norinder *et al*, 2020; Du *et al*, 2021; Ou *et al*, 2021; Yuan *et al*, 2022). The identification in this work of two novel SARS‐CoV‐2 entry host factors linked to autophagy and lysosome regulation reinforces the importance of this pathway for therapeutic interventions and defines new target opportunities.

In summary, we have demonstrated herein that *SPNS1* and *PLAC8* are host factors necessary for SARS‐CoV‐2 entry and that *PLAC8* is a limiting factor that enables human pulmonary cells for infection. These findings expand the complex network of host factors required for the SARS‐CoV‐2 life cycle and provide novel insights for a better understanding of the mechanisms of viral pathogenesis. Hopefully, this knowledge may facilitate the development of the urgently needed host‐directed therapies against a coronavirus that has already caused the death of more than six million people around the world and has dramatically unveiled the multiple social, economic, political, scientific, and medical vulnerabilities of current human societies.

## Material and Methods

### Cell culture, buffers, and reagents

Cell lines were obtained from ATCC and cultured using either D10F (Calu‐1, Calu‐3, SW‐900, VeroE6, and HEK‐293T) or R10F (NCI‐H226, NCI‐H358, NCI‐H460, NCI‐H661, NCI‐H1155, NCI‐H1299, NCI‐H1568, NCI‐H1792, NCI‐H1993, NCI‐H2009, and A549) medium. R10F medium was composed of Roswell Park Memorial Institute medium (RPMI‐1640, Gibco) supplemented with 10% (v/v) fetal bovine serum (FBS, Gibco). D10F medium was composed of Dulbecco's Modified Eagle Medium (DMEM, Gibco) supplemented with 10% (v/v) FBS. Additionally, all media were supplemented with 1% (v/v) of 100× Antibiotic‐Antimycotic (Gibco) and 1% (v/v) of 100× Penicillin–streptomycin‐glutamine (Gibco). For transduction, lentiviral supernatants were supplemented with 0.8 μg/ml polybrene (Santa‐Cruz, sc‐134220). Transduced cells were then selected with 10 μg/ml blasticidin S (Gibco), 2 μg/ml puromycin (Sigma‐Aldrich), 500 μg/ml hygromycin (Invitrogen), or 1 mg/ml geneticin (G418, Gibco) as indicated. Stock solutions were prepared in dimethyl sulfoxide (DMSO, Sigma‐Aldrich) or 1× PBS and directly added to the culture medium to obtain the final working concentrations. An equal volume of drug solvent (PBS or DMSO) was added to the control cells. All human parental cell lines were tested for mycoplasma using the method described in (Young *et al*, 2010). The identity of the main cell lines used throughout the manuscript (Calu‐1, NCI‐H1299, NCI‐H226, NCI‐H358, NCI‐H1792, NCI‐H2009), as well as A549 and Calu‐3, were confirmed by STR genotyping at the core facilities of the University of Oviedo.

For immunoblotting experiments, the NP‐40 lysis buffer contained 50 mM Tris–HCl pH 7.5, 150 mM NaCl, 10 mM EDTA, and 1% (v/v) NP‐40 (Sigma‐Aldrich). NP‐40 lysis buffer was supplemented with a complete EDTA‐free protease inhibitor cocktail (Roche), as well as 10 mM NaF (Merck) and PhosSTOP phosphatases inhibitor cocktail (Roche) before use. For SDS–PAGE, protein samples were mixed with 4X SDS–PAGE loading buffer containing 200 mM Tris–HCl pH 6.8, 8% (w/v) sodium dodecyl sulfate (SDS), bromophenol blue (1 mg/ml), 40% (v/v) glycerol and 2% (v/v) β‐mercaptoethanol (all from Sigma‐Aldrich). TBS‐T buffer contained 20 mM Tris–HCl pH 7.4, 150 mM NaCl and 0.05% (v/v) Tween‐20 (Sigma‐Aldrich). Information about the oligonucleotides and antibodies used in this work can be found in Appendix Table S1 and S2.

### DNA constructs

Lv‐SFFV‐Ace2‐IRES‐Neo was purchased from Addgene (Plasmid #145840). Lv‐SFFV‐Ø‐Neo was generated by replacing ACE2 with annealed oligonucleotides (MCS_f and MCS_rv, Appendix Table S1) using *Not*I and *Xho*I restriction sites of the Lv‐SFFV‐Ace2‐IRES‐Neo vector. pLC‐ZsGreen‐P2A‐Hygro was purchased from Addgene (Plasmid #124301). pLC‐mCherry‐P2A‐Hygro was generated by replacing *ZsGreen* with *mCherry* gene from Lenti‐EF1a‐mCherry‐P2A‐Hygro (Plasmid #135003) with *Age*I and *Bam*HI. Lv‐SFFV‐G418‐GFP and Lv‐SFFV‐G418‐ACE2‐GFP were generated by inserting PCR‐amplified GFP or ACE2‐GFP from pcDNA3‐ACE2GFP Addgene plasmid #154962 (using oligonucleotides SFFV_ACE2, ACE2GFP_SFFV and SFFV_GFP) into Lv‐SFFV‐Ø‐Neo opened with *Bam*HI, using NEBuilder HiFi DNA Assembly Master Mix (NEB). Lv‐SFFV‐G418‐tagRFP was generated by inserting PCR‐amplified tagRFP from pLEX301‐tagRFP Addgene plasmid #162035 (using oligonucleotides RFP_SFFV and SFFV_RFP) into Lv‐SFFV‐Ø‐Neo opened with *Bam*HI, using NEBuilder HiFi DNA Assembly Master Mix (NEB). Lv‐SFFV‐G418‐ACE2‐tagRFP was generated by simultaneously inserting PCR‐amplified tagRFP from pLEX301‐tagRFP (using oligonucleotides ACE2RFP_5 and RFP_SFFV) and ACE2 from pcDNA3‐ACE2GFP (using oligonucleotides SFFV_ACE2 and ACE2_3) into Lv‐SFFV‐Ø‐Neo opened with *Bam*HI, using NEBuilder HiFi DNA Assembly Master Mix (NEB). For pseudotyping lentiviral particles, pcDNA3.1‐SARS2‐Spike‐Δ19 (Plasmid#155297) and pMD2.G containing VSV gene (Plasmid #12259) were purchased from Addgene. Full‐length *PLAC8* transcript variant 1, and *SPNS1* transcript variant 1, cloned with an in‐frame N‐terminal eGFP tag in the pcDNA3.1(+)‐Neo vector, were purchased from GenScript. In addition, *PLAC8* and *SPNS1* genes were customs modified to prevent gRNA targeting without altering amino acid sequence. Then, GFP‐PLAC8 and GFP‐SPNS1 constructs were subcloned into pCDH‐CMV‐EGFP‐EF1‐Blasticidin lentiviral vector (Bretones *et al*, 2018: 1) with *Nhe*I and *Xho*I. The human CRISPR gRNA pooled library in lentiCRISPRv2 (GeCKO v2) was purchased from Addgene (Pooled Library #1000000048). All the LentiCRISPRv2 vectors for screen validation were generated by inserting the respective forward (fw) and reverse (rv) hybridized oligonucleotides (Appendix Table S1) in between *Bsm*BI restriction sites of the LentiCRISPRv2‐Puro vector (Plasmid #52961). Lentiviral packaging plasmid psPAX2 (Plasmid #12260) was purchased from Addgene. All constructs were sequenced and validated before experimental use.

### Lentiviral methods

Lentivirus production was performed in the same way for genetic manipulation and pseudotyped lentiviral infections. HEK‐293T cells growing in 10 cm dishes were transfected with 5 μg of a lentiviral helper plasmid (psPAX), 5 μg of lentiviral plasmid, and 5 μg of the plasmid expressing the viral membrane protein (either pcDNA3‐Spike‐Δ19 for S‐typed or pMD2.g for VSVG‐typed lentiviruses), using Lipofectamine 3000. After overnight incubation, the transfection medium was replaced with a 10 ml fresh growth medium, and 48 h later, viral supernatants were collected and filtered through a 0.45 μm filter. Lentiviral production for CRISPR‐Cas9 genome‐editing or genetic manipulation (*ACE2*, *PLAC8*, and *SPNS1* overexpression) was performed in the same way using the VSVG‐expressing plasmid pMD2.g and the corresponding lentiviral plasmid.

### CRISPR‐Cas9 genome‐wide screen

First, Calu1^ACE2^‐GeCKO cells were generated by transducing Calu1^ACE2^ with the plentiCRISPR GeCKO v.2.0 library (library A + B, approximately 130,000 sgRNAs). For this purpose, around 230 million Calu1^ACE2^ cells were seeded in 15 cm dishes at 4.5 million cells per dish. Next day, previously produced GeCKO plentiCRISPR lentiviral supernatants were applied at a low MOI (250 μl viral supernatant in a 15 ml growth medium dish supplemented with 0.8 μg/ml polybrene) to achieve an approximately 25% infection rate. After 8 h, the medium was refreshed, and cells were allowed to recover for 48 h before starting selection with puromycin (2 μg/ml). The selection was allowed for 6 days until all cells of a noninfected dish had died. At this point, Calu1^ACE2^‐GeCKO were pooled, and 340 million cells were seeded in 15 cm dishes for screening. Next day, 2/3 of the cells were infected with Spike‐Δ19 pseudotyped lentiviral supernatants carrying a ZsGreen‐IRES‐Hygromycin plasmid (15 ml per dish supplemented with 0.8 μg/ml polybrene), while the remaining 1/3 was left uninfected as the control population. After overnight incubation, viral supernatants were removed, and a fresh growth medium was added to the dishes. Infection efficiency was estimated at 25% using a fluorescence microscope at 48 h postinfection. Selection of S‐typed infected cells was done by culturing in hygromycin‐containing medium (Invitrogen, 500 μg/ml final concentration) for 10 days until all cells in a noninfected dish had died. The uninfected population was cultured in parallel without hygromycin selection. Both the infected and noninfected populations were split when necessary and seeded keeping always a 1,000× representation (130 million cells). At the end of the screen, cells were detached, pooled, washed with PBS, and stored for genomic DNA isolation and NGS analysis.

Genomic DNA extraction was done using classic phenol/chloroform extraction. Briefly, cell pellets (approximately 8 million per pellet) were resuspended in 300 μl of PBS containing 3 mg/ml RNAse A and incubated for 30 min at 37°C. Then, 300 μl of lysis buffer (Tris 200 mM, pH 7.4, EDTA 200 mM, 1% SDS) and 20 μl of proteinase K (19 mg/ml – 2.5 U/mg, Roche) were added to each tube. After 2 h incubation at 55°C, samples were cooled down to 37°C and another RNA digestion was performed for 30 min at 37°C by adding fresh RNAse A. Then, 400 μl of phenol/TE and 400 μl of chloroform/isoamyl alcohol, 24:1 (v:v) were added and samples were vortexed vigorously. Phase separation was performed by centrifugation for 15 min at 13,000 rpm. To increase phenol removal, a second chloroform step was performed on the aqueous phase. DNA precipitation was done by the addition of 0.5 vol of ammonium acetate (7.5 M, pH 5.5) and 1 vol of isopropanol. After mixing, samples were centrifuged for 5 min at 16,000 *g* and DNA pellets were washed with 70% ethanol before air drying and resuspension in TE buffer. Genomic DNA was quantified using Qubit DNA broad‐range assays (Thermo Fisher Scientific).

For NGS library preparation, we used a two‐step PCR protocol using custom primers (Appendix Table S1) and Q5 high fidelity polymerase (NEB). For each condition (S‐typed infected and noninfected cells), 900 μg of genomic DNA—equivalent to a 1,000× coverage assuming 6 pg of genomic DNA per cell—were PCR amplified in two 96‐well plates. Each reaction (50 μl) contained approximately 4 μg of genomic DNA and 0.5 μM of primers NGS_Gecko_rev and NGS_ATA_fwd (S‐typed infected cells) or NGS_TAA_fwd (noninfected cells). Each forward primer contains an in‐read barcode and a 10 nt random sequence that serves as a unique molecular identifier (UMI). Genomic DNA was amplified for 6 cycles using the following program: 98°C – 15 s denaturation, 64°C – 30 s annealing, and 68°C – 30 s extension. Subsequently, PCR reactions for each sample were pooled together and purified with Ampure XP beads using a double‐size selection approach to remove primers and genomic DNA. Briefly, a first size selection was done using 0.5 vol of beads and the supernatant containing the PCR products and primers was collected. Then, another 0.5 vol of beads was added to the samples to induce binding of the PCR products. Beads were then washed with 70% ethanol and DNA was eluted in TE buffer. For the second PCR, 12 × 50 μl reactions were performed using half of the purified first PCR products and 0.5 μM of primers NGS2_fwd and NGS2_rev10 (S‐typed cells) or NGS2_rev20 (noninfected cells). We used 15 amplification cycles of the same PCR program. For final library purification, two rounds of Ampure XP beads selection (1:1 ratio) were applied and the DNA was eluted in TE buffer. The purified libraries were quantified using Qubit broad‐range assays and equimolar amounts of each library were pooled and submitted for NGS on an Illumina HiSeqX lane using 150 cycles of pair‐end sequencing. Approximately 150 million pair‐end reads were obtained for each sample. For NGS analysis, pair‐end reads were first merged using NGMerge (Gaspar, 2018), and then, UMI sequences were extracted using UMItools (Smith *et al*, 2017). Afterward, cutadapt (Martin, 2011) was used to remove adaptor sequences, and reads shorter than 20 nt after trimming were filtered out. To compute sgRNA abundances, reads were first aligned to a custom sgRNA genome index using Bowtie2 (Langmead & Salzberg, 2012), and then, perfect 20 nt matches were deduplicated using UMItools. After all these filtering steps, we ended up with around 60 million deduplicated alignments per sample. Final counting was performed using Bash commands *awk*, *sort*, and *uniq*. sgRNA enrichment statistics and gene‐level scores between S‐typed and noninfected populations were computed using Mageck software with nontargeting sgRNA normalization (Li *et al*, 2014).

### Pseudotyped lentiviral infections assays

The day before infection, cell lines were detached and counted, and 7,500 cells per well were seeded in 96‐well plates. Next day, infections were performed using 50 μl of a 50:1 mixture of S‐typed and VSVG‐typed viral supernatants, supplemented with polybrene (0.8 μg/ml). After overnight incubation, the medium was aspirated and 100 μl of growing medium was added to the plates. After 48 h, cells were detached using trypsin and analyzed in a Beckman Coulter Cytoflex S cytometer. When possible, 20,000 total events were recorded for every sample. Samples with less than 5,000 total events were discarded. Flow cytometer data were analyzed using R language and packages flowCore and ggplot2 (Hahne *et al*, 2009; Wickham, 2009). Briefly, the cell population was defined using F/SSC gating and the percentage of positive cells was calculated using a manually defined cutoff based on log10 fluorescence intensity histograms of the corresponding channel on a noninfected population.

### SARS‐CoV‐2 infection assays

All work with SARS‐CoV‐2 viruses was performed in a BSL3+ laboratory at the Animal Health Research Center (CISA, at INIA‐CSIC, Valdeolmos). The SARS‐CoV‐2 viral strain used in this study (named CISA/H‐Ap20‐1) was obtained by isolation from an RT–PCR‐positive nasopharyngeal swab sample (Ct 19.24 using the E‐gene protocol described in (Corman *et al*, 2020), with slight modifications) from an asymptomatically infected, occupationally‐exposed worker giving essential service in Madrid (Spain), who was also seronegative at the time of sampling (April, 19^th^ 2020). The consensus sequence is available from GISAID with accession number EPI_ISL_770129 (González‐Recio *et al*, 2021). The sample was obtained in April 2020 (during the first epidemic wave taking place in Madrid and under lockdown and other strict control measures) during a SARS‐Cov‐2 screening of essential personnel from Madrid City Hall essential services (Police, Firemen, Emergency, Health Care Workers, etc) (Martínez‐Cortés *et al*, 2021). The sample was inoculated (100 μl/well) into semiconfluent VR E6 cells cultured in 12‐well plates, incubated at 37°C with 5% CO_2_, and observed for cytopathic effect (CPE) daily up to 7 days postinoculation. Wells developing CPE were collected and subjected to one additional passage (#2) in VR E6 cells for virus propagation. Clarified supernatants of passages #1 and #2 were quantified by RT–PCR to confirm the success of viral isolation, as well as to assess viral growth. Aliquots from passage #2 of this SARS‐CoV‐2 isolate were used throughout this study. The day before infection, 15,000 cells per well were seeded in 96‐well plates (Black/clear tissue culture‐treated plates, BD Falcon). Next day, the medium was removed and 50 μl of SARS‐CoV‐2 viral supernatant (1:100 diluted in DMEM or RPMI supplemented with 1% fetal bovine serum and antibiotics) were applied. After 24 h, virus‐containing medium was removed, and cells were washed with PBS and fixed/neutralized with 4% paraformaldehyde in PBS for 15 min at room temperature. For immunofluorescence of SARS‐CoV‐2 nucleocapsid protein, cells were permeabilized for 15 min using 1% Triton X‐100 in PBS and blocked with 10% goat serum in PBST (PBS Triton X100 0.5%) for 30 min. Then, cells were incubated with human anti‐SARS‐CoV‐2 nucleocapsid antibody for 2 h at room temperature. After 3 washes with PBST, cells were incubated with goat‐anti‐human FITC secondary antibody (1/1,000 diluted in PBST) for 1 h at room temperature. For GFP‐expressing cell lines, a goat‐anti‐human Alexa Fluor 568 (1/1,000 diluted in PBST) secondary antibody was used. Finally, cells were washed three times with PBST and counterstained with DAPI (Roche, Ref. 10236276001, 1 μg/ml in PBS). Imaging was performed in a Zeiss Axiovert fluorescence microscopy using 10× magnification. At least 4 fields per well were captured. Quantification of the number of infected cells was performed using ImageJ (Schindelin *et al*, 2012) and a custom macro that uses functions *threshold* and *analyzes particles* on the DAPI channel to define nuclei as regions of interest (ROI) that are then used to measure the fluorescence intensity on the red channel (nucleocapsid staining). The output of ImageJ, containing the average fluorescence intensity for every nucleus in every well, was then further analyzed using R language to calculate the proportion of positive cells per sample. Basically, the number of infected cells was calculated as the number of nuclei with a mean fluorescence intensity greater than a cutoff that was defined using noninfected cells. To reduce false positives, positive nuclei with Euclidean distances between their centroids smaller than 30 μm were clustered as a single positive cell.

### Single‐cell RNA‐Seq analyses

scRNA‐Seq datasets from lung (Vieira Braga *et al*, 2019; Deprez *et al*, 2020), ileum (Martin *et al*, 2019), and colon (Smillie *et al*, 2019) samples from healthy donors were downloaded from the UCSC Cell Browser (https://cells.ucsc.edu). The datasets of lung samples from COVID‐19 patients were downloaded from the UCSC Cell Browser (Chua *et al*, 2020) and from http://covid19.lambrechtslab.org (Wauters *et al*, 2021). In all cases, the gene expression analyses were done in R language using packages *ggplot2* (Wickham, 2009) and *data.table*, and the downloaded gene expression matrices and associated metadata from the different resources.

### Flow cytometry immunofluorescence

For cell surface ACE2 immunofluorescence, cells were detached with versene (Gibco) and washed with ice‐cold PBS by centrifugation at 500 *g* for 5 min at 4°C. After that, approximately 2 million cells were incubated on ice for 45 min in 100 μl FACS buffer (PBS supplemented with 1% FBS and 2 mM EDTA) containing anti‐ACE2 antibody (2 μg/ml; R&D MAB9332). After three washes with ice‐cold PBS, cells were incubated on ice for 30 min with the secondary antibody (goat‐anti‐mouse Alexa Fluor 546) in 100 μl FACS buffer. Cells were then washed three times with ice‐cold PBS and resuspended in 100 μl FACS buffer containing 0.2 μg/ml DAPI. Around 100.000 total events per sample were recorded in a Beckman Coulter Cytoflex S cytometer. Flow cytometry analyses were performed using R language and packages *flowCore*, *data.table*, and *ggplot2* (Hahne *et al*, 2009; Wickham, 2009). Briefly, cells were first gated based on FSC and SSC intensities followed by a DAPI exclusion filter. Density plots were done using the R‐base function *density* and package *ggplot2*. For bar plots, the median fluorescence intensity (MFI) was used.

### Western blotting

Cells were lysed in NP‐40 lysis buffer containing protease and phosphatase inhibitors cocktails (Roche). Protein concentration was determined using Pierce^®^ BCA Protein Assay Kit (Thermo Fisher Scientific). Equal amounts of protein (20–30 μg) were resolved by 4 to 20% SDS–PAGE gels (Bio‐Rad) and transferred to Immobilon‐FL PVDF membranes (GE Healthcare Life Sciences). Membranes were blocked for 1 h at room temperature with TBS‐T (0.1% Tween 20) containing 5% BSA. Immunoblotting was performed with primary antibodies (Appendix Table S2) diluted 1:500 to 1:1,000 in TBS‐T containing 1% BSA and incubated overnight at 4°C. After washing with TBS‐T, the membranes were incubated with secondary antibodies conjugated with IRDye^®^ 680RD or IRDye^®^ 800CW (LI‐COR) for 1 h at room temperature. Protein bands were visualized and recorded with LI‐COR Odyssey Imaging System (LI‐COR). Western blots in Figs 1A and 3C were developed using HRP conjugated secondary antibodies and Immobilon Forte HRP substrate (Millipore) in a Fujifilm LAS‐3000 imaging system.

### Immunoprecipitation

Cells cultured in a growth medium in 10 cm dishes (approximately 5 × 10^6^ cells per immunoprecipitation) were washed with PBS, detached with versene (Gibco), and resuspended in ice‐cold co‐IP lysis buffer (50 mM Tris–HCl pH 7.4, 150 mM NaCl, 10 mM EDTA, 0.1% Triton X‐100), supplemented with protease and phosphatase inhibitor cocktails. After a 30 min incubation on ice, cell lysates were clarified by centrifugation at 16,000 *g* for 20 min in a refrigerated microcentrifuge. Protein concentration was determined using Pierce® BCA Protein Assay Kit (Thermo Scientific) and 1 mg of total protein extract per IP was incubated overnight at 4°C with 3 μg of rabbit polyclonal anti‐GFP antibody (Living Colors®, Takara) and 0.1 μg of recombinant 6xHis‐tagged full‐length SARS‐CoV‐2 Spike protein (10561‐CV, R&D systems). The next day, immunocomplexes were recovered by 2 h incubation with lysis buffer‐equilibrated Dynabeads®‐protein G (Invitrogen, 25 μl per IP). Then, beads were washed five times with co‐IP lysis buffer (1 ml per wash) using a magnet DynaMag™ (Invitrogen) and eluted by boiling in 40 μl of 1X SDS–PAGE loading buffer. Finally, 15 μl of unbound fraction and 15 μl of immunoprecipitated samples were loaded onto 4–20% gradient polyacrylamide gels and electrophoresed for western blot analysis.

### Protein quantification by liquid chromatography coupled to tandem mass spectrometry

Proteins from NT596, *PLAC8*‐KO1, and *SPNS1*‐KO1 CALU1^ACE2^ cells (each with three biological replicates) were on‐filter digested with modified porcine trypsin (Promega) at a final ratio of 1:40 (trypsin‐protein). Digestion proceeded overnight at 37°C in 100 mM ammonium bicarbonate, pH 7.8. The resulting tryptic peptides were labeled with TMT‐10plex (Thermo Scientific), according to the manufacturer's instructions. The resulting peptides were injected onto a C‐18 reversed phase (RP) nano‐column (75 mm I.D. and 50 cm, Acclaim PepMap, Thermo Fisher, San José, CA, USA) and analyzed in a continuous acetonitrile gradient consisting of 8–31% B for 240 min, 50–90% B for 1 min (B = 0.5% formic acid in acetonitrile). Peptides were eluted from the RP nano‐column at a flow rate of ~200 nL/min to an emitter nanospray needle for real‐time ionization and peptide fragmentation in a Q‐Exactive HF mass spectrometer (Thermo Scientific). Mass spectra were acquired in a data‐dependent manner, with an automatic switch between MS and MS/MS using a top 20 method. An enhanced FT‐resolution spectrum (resolution = 70,000) followed by the MS/MS spectra from the most intense 20 parent ions were analyzed along the chromatographic run (272 min). Dynamic exclusion was set at 30 s. For protein identification, tandem mass spectra were extracted and the charge state deconvoluted by Proteome Discoverer 2.1 (Thermo Fisher Scientific). All MS/MS samples were analyzed using SEQUEST (Thermo Scientific), using with a precursor mass tolerance of 800 ppm and a fragment mass tolerance of 0.03 amu. Carbamidomethylation in cysteine, TMT‐label in N terminus, and TMT‐label in lysine were set as fixed modifications, and oxidation in methionine as a variable modification. The false discovery rate (FDR) was calculated based on the search of results against the corresponding decoy database using the refined method (Navarro & Vázquez, 2009) with an additional filter for precursor mass tolerance of 15 ppm (Bonzon‐Kulichenko *et al*, 2015) and estimation of the corrected Xcorr (Martínez‐Bartolomé *et al*, 2008). An FDR of 1% was used as the criterion for peptide identification. Each peptide was assigned only to the best protein proposed by the Proteome Discoverer algorithm. Quantitative information was extracted from MS/MS spectra, from TMT reporter ions, using an in‐house developed program (SanXoT), as described in (Navarro *et al*, 2014), and protein abundance changes were analyzed using the Generic Integration Algorithm, as described in (García‐Marqués *et al*, 2016). Quantitative information from TMT reporter intensities was integrated from the spectrum level to the peptide level and then to the protein level based on the WSPP model (Navarro *et al*, 2014) using the GIA integration algorithm (García‐Marqués *et al*, 2016) with the SanXoT bioinformatics package (Trevisan‐Herraz *et al*, 2019). The validity of the null hypothesis at each one of the levels (spectrum, peptide, protein within an experiment, and protein) was carefully checked by plotting the cumulative distributions, as described in (Navarro *et al*, 2014). Relative changes in protein abundance (log2‐ratios) were expressed in standardized units (*Zq*; Navarro *et al*, 2014). Differential expression analysis was performed on the log2 transformed relative abundances using linear model fitting and empirical Bayes moderated *t*‐statistics from *limma* R‐package combined with Benjamini‐Hochberg multiple test correction (Ritchie *et al*, 2015). Gene Set Enrichment Analysis (GSEA) was performed on the proteomics data using preranked GSEA with the metric ‐log10(*P*‐value)*sign(LFQ differences) using GSEA software (Subramanian *et al*, 2005). Bubble plots and volcano plots were generated using R language with package *ggplot2*. Venn diagrams to compare the overlap between *PLAC8*‐KO and *SPNS1‐*KO were generated using the R‐package *nVennR* (Pérez‐Silva *et al*, 2018).

### Autophagolysosomal studies

For immunofluorescence analyses, cells were grown on 96‐well black clear tissue culture‐treated plates, washed in PBS, and fixed in 4% paraformaldehyde in PBS at room temperature for 10 min. Primary antibody was diluted at 1:100 in PBS and incubated overnight at 4°C. Samples were washed three times in PBS for 15 min each. Secondary antibody was diluted at 1:300 in PBS and incubated at RT for 1 h. Samples were washed three times in PBS for 15 min each and analyzed by fluorescence microscopy. The following primary antibodies were used: anti‐SQSTM1, anti‐LC3B, and anti‐LAMP1 (Appendix Table S2). Fluorescence microscopy images were acquired with a Carl Zeiss Axio Observer Z1 platform equipped with a Plan‐Apochromat 40X/1.3. For lysotracker staining, cells growing on 96‐well plates at an approximately 70% confluence were incubated with 50 nM lysotracker (LysoTracker™ Red DND‐99, Thermo Fisher Scientific) in a growth medium for 30 min at 37°C and 5% CO_2_. Then, the medium was refreshed, and images were captured in a Carl Zeiss Axio Observer Z1 platform with 5% CO_2_ incubation at 37°C. Image analyses and dot number quantification was done using the MorphoLibJ ImageJ plugin with the *white top‐hat* morphological filter (Legland *et al*, 2016). Images for colocalization studies between lysotracker and *GFP‐PLAC8* and *GFP‐SPNS1* were taken in a Leica White Laser Confocal Microscope TCS‐SP8X equipped with CO_2_ and temperature incubation. For lysosensor staining, cells growing on 96‐well plates at an approximately 70% confluence were incubated with 1 μM lysosensor (LysoSensor™ Yellow/Blue DND‐160, Thermo Fisher Scientific) in a growth medium for 5 min at 37°C and 5% CO_2_. Then, the medium was refreshed, and images were captured in a Carl Zeiss Axio Observer Z1 platform with 5% CO_2_ incubation at 37°C. Lysosensor ratio determination (Ex329/Ex384) was performed using the spots colocalization (ComDet) ImageJ plugin.

For autophagy flux measurement, cells were transduced with the reporter plasmid GFP‐LC3‐RFP‐LC3ΔG (Kaizuka *et al*, 2016). After selection with puromycin, cells were allowed to recover and seeded in 24‐well plates. Next day, cells were incubated for 6 h in growth conditions (complete medium) or subjected to starvation (Earle's Balanced Salts, EBSS), BafA1 inhibition (complete medium +50 nM BafA1) or starvation + BafA1 (EBSS +50 nM BafA1). Then, cells were detached using trypsin, washed with PBS, and resuspended in FACS buffer. Cells were kept on ice until flow cytometry analysis in a Beckman Coulter Cytoflex cytometer. For dead cell exclusion, DAPI was added to the FACS buffer at a final concentration of 2 nM. FCS/SCC gating was used to select the main cell population, and then, the DAPI signal was used to filter out dead cells. Afterward, GFP/RFP scatter plots were used to filter out cells that underwent recombination (negative for either GFP or RFP) or cells without reporter expression. The remaining events were used to calculate the ratio between RFP and GFP signal. All data analyses were done using R language and packages *flowCore*, *data.table*, and *ggplot2*.

For lysosomal degradation studies, approximately 10^4^ Calu1^ACE2^ cells per well were seeded in 96‐well plates 24 h before the experiment. Next day, cells were either left untreated or serum starved (with EBSS) for 5 h. Then, cells were washed with PBS and incubated for 30 min at 37°C with 100 μl of EBSS containing 10 μg/ml of DQ‐Red‐BSA (Thermofisher Scientific). Next, cells were detached using phenol‐free trypsin and blocked/resuspended in 100 μl of ice‐cold FACS buffer containing DAPI (200 nM final concentration). Cells were kept on ice until data acquisition in a Beckman Coulter Cytoflex cytometer. Flow cytometry events were first gated based on FSC and SSC intensities followed by a DAPI exclusion filter. Data analysis was done using R language and packages *flowCore*, *data.table*, and *ggplot2*.

### Binding and endocytosis experiments

For flow cytometry analyses, cells growing in 10 cm dishes were detached using versene and resuspended in a complete medium without phenol red (DMEM w/o phenol red supplemented with 10% FBS, nonessential amino acids, and glutamine) at approximately 1 million cells per ml. Cell suspension was cooled down to 4°C on ice, and then, S‐RBD‐ or transferrin‐Alexa Fluor 647 (Appendix Table S2) was added at 1 μg/ml final concentration. After 1 h at 4°C with occasional shaking, an aliquot was removed to measure binding and the remaining cells were incubated at 37°C for 2 h with shaking. For binding, cells were washed twice with ice‐cold PBS, fixed with 4% p‐formaldehyde in PBS for 15 min, washed again twice with PBS, and resuspended in FACS buffer. For endocytosis experiments, cells were washed twice in acid wash buffer (50 mM glycine pH 2.5, 150 mM NaCl, 1 mM CaCl_2_, 4 mM KCl, 0.5 mM MgCl_2_) to remove bound ligand and then processed in the same way. Data acquisition was performed in a Beckman Coulter Cytoflex cytometer. Although every fluorophore uses a different laser line (tagRFP, EGFP, and Alexa Fluor 647), a compensation matrix is created separately from single color controls (parental cells, cells with ACE2‐RFP expression, cells with GFP expression, and parental cells treated with transferrin Alexa Fluor 647) was applied. FS/SSC gating was used to select the main cell population. All data analyses were done using R language and packages *flowCore*, *data.table*, and *ggplot2*.

For microscopy experiments, approximately 8,000 cells per well were seeded in 18‐well ‐slide polymer chambered coverslips (IBIDI) coated with poly‐L‐lysine. After 48 h, cells were treated with S‐RBD Alexa Fluor 647 (1 μg/ml) in a complete medium without phenol red. To measure binding, cells were precooled at 4°C before ligand addition and then incubated for 1 h at 4°C. Afterward, cells were washed twice with ice‐cold PBS or acid wash buffer and then fixed with 4% p‐formaldehyde in PBS for 15 min at RT. For endocytosis, the ligand was added at room temperature and cells were further incubated for 1 h at 37°C. Next, coverslips were cooled down to 4°C and processed in the same way. Wide‐field images were taken in a Zeiss AxioObserver microscope using a Plan‐Apochromat 63x/1.4 oil objective and a Colibri 5 light source. ImageJ software was used to measure the mean intensity of tagRFP, EGFP, and Alexa Fluor 647 within each cell (defined manually using the freehand selection tool). For colocalization studies, samples were microphotographed using a Leica White Laser Confocal Microscope TCS‐SP8X, and images were analyzed using the ImageJ ComDet spot colocalization plugin. Data analysis and representation were done using R language and ggplot2 package.

### Experimental design and statistics

In most experiments, at least 3 independently generated cell lines per experimental group (biological replicates) were analyzed to provide statistical analysis. All figures in this work correspond to single experiments with the number of biological or technical replicates indicated in the figure legends and represented by points. No randomization or blinding techniques were used. In some flow cytometry experiments, samples with < 5,000 events were excluded (pseudotyped lentiviral infections assays section). All statistical tests, data analysis, and plots were generated using R and RStudio (R Core Team, Vienna, Austria, https://www.r‐project.org; RStudio Team, Boston, MA, USA, https://www.rstudio.com). Unless otherwise indicated, bar plots represent the mean and standard error of the mean (SEM), and the statistical significance was determined by the Student's unpaired two‐tailed *t*‐test (treating the group variances as independent) assuming normality. In experiments involving multiple measures per sample (i.e., immunofluorescence‐based autophagolysosomal studies) significance was determined by the Wilcoxon unpaired two‐sample test, and boxplots representing the median and interquartile range were used for representation (unless otherwise indicated).

## Author contributions

**Alejandro P Ugalde:** Conceptualization; data curation; formal analysis; supervision; validation; investigation; visualization; methodology; writing – original draft; writing – review and editing. **Gabriel Bretones:** Conceptualization; data curation; formal analysis; supervision; validation; investigation; visualization; methodology; writing – original draft; writing – review and editing. **David Rodríguez:** Investigation; writing – review and editing. **Victor Quesada:** Investigation; writing – review and editing. **Francisco Llorente:** Investigation. **Raul Fernández‐Delgado:** Investigation. **Miguel Ángel Jiménez‐Clavero:** Investigation; methodology. **Jesús Vázquez:** Investigation. **Enrique Calvo:** Investigation. **Isaac Tamargo‐Gómez:** Investigation. **Guillermo Mariño:** Investigation. **David Roiz‐Valle:** Investigation. **Daniel Maeso:** Investigation. **Miguel Araujo Voces:** Investigation. **Yaiza Español:** Funding acquisition; project administration; writing – review and editing. **Carles Barceló:** Investigation. **Jose MP Freije:** Conceptualization; supervision; funding acquisition; project administration; writing – review and editing. **Alejandro López‐Soto:** Conceptualization; supervision; funding acquisition; project administration; writing – review and editing. **Carlos Lopez‐Otin:** Conceptualization; supervision; funding acquisition; writing – original draft; project administration; writing – review and editing.

## Disclosure and competing interests statement

The authors declare that they have no conflict of interest.

## Supporting information



Source Data for Appendix

Appendix S1

Dataset EV1

Dataset EV2

Source Data for Expanded View

Source Data for Figure 1

Source Data for Figure 2

Source Data for Figure 3

Source Data for Figure 5

## Data Availability

High‐throughput quantitative proteomics data are available via ProteomeXchange with identifier PXD036334 (https://www.ebi.ac.uk/pride/archive/projects/PXD036334).
