# Supplementary material for: Autophagy‐linked plasma and lysosomal membrane protein PLAC8 is a key host factor for SARS‐CoV‐2 entry into human cells
Source: EMBO J. 2022 Oct 4;41(21):e110727. doi: 10.15252/embj.2022110727 (PMC9627672; doi:10.15252/embj.2022110727)
Supplement: Supplementary file 2 — Appendix S1 [file EMBJ-41-e110727-s004.pdf]

Appendix

Autophagy-linked plasma and lysosomal membrane protein PLAC8 is a key host factor for SARS-CoV-2 entry into human cells

Ugalde et al.

Content

Appendix Figure 1.. 2
Appendix Figure 2.. 3
Appendix Figure 3.. 4
Appendix Figure 4.. 5
Appendix Figure 5.. 6
Appendix Figure 6.. 7
Appendix Figure 7.. 8
Appendix Figure 8.. 9
Appendix Figure 9.. 10
Appendix Table 1 11
Appendix Table 2 12

Appendix Figure S1

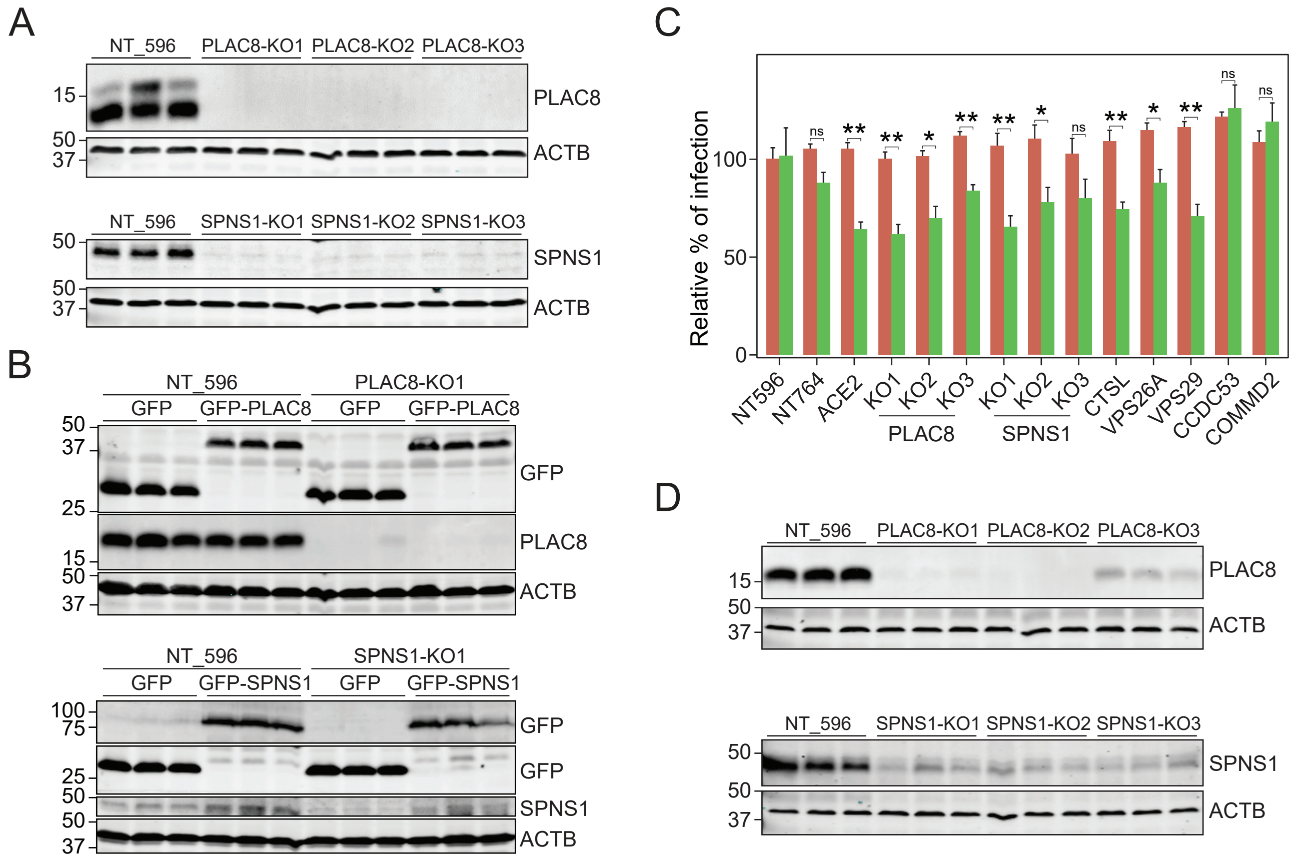

Appendix Figure S1: A) Western-blot analysis of PLAC8 and SPNS1 protein levels in CRISPR-KO Calu1^{ACE2} cells. **B)** Western-blot analysis of endogenous PLAC8 and SPNS1 levels and PLAC8- and GFP-SPNS1 overexpression in rescue experiments in Calu1^{ACE2} cells. **C)** Susceptibility to infection with Spike- and VSVG-typed lentiviruses in H1299^{ACE2} cells with loss-of-function in the genes analyzed in Figure 2B. Bars represent the average and standard error of the mean of three biological replicates. Significance: ns, p-value >= 0.05, * p-value < 0.05, ** p-value < 0.01. **D)** Western-blot analysis of PLAC8 and SPNS1 protein levels in CRISPR-KO H1299^{ACE2} cells. Actin was used as loading control.

Appendix Figure S2

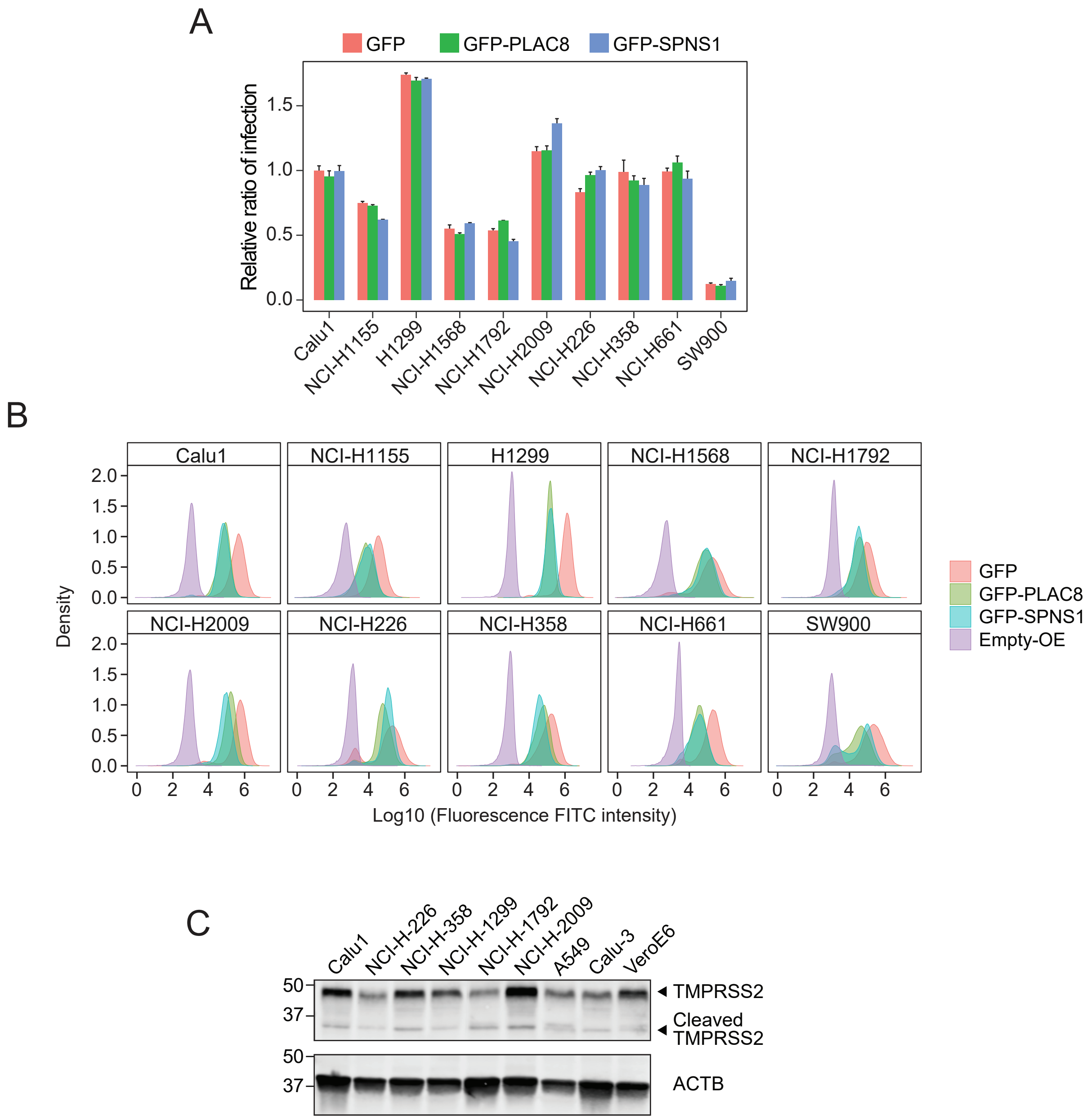

Appendix Figure S2: A) Susceptibility to infection with VSVG-typed lentiviruses in the same cell lines analyzed in Figure 3D. Bar and whiskers represent the mean and SEM of three biological replicates. **B)** Density plots of GFP intensity showing the overexpression levels of GFP, GFP-PLAC8 and GFP-SPNS1 in the cell lines analyzed in Figure 3D. Empty-OE: parental cells without GFP expression. **C)** Western-blot analysis of TMPRSS2 in several parental cell lines. Actin was used as loading control.

Appendix Figure S3

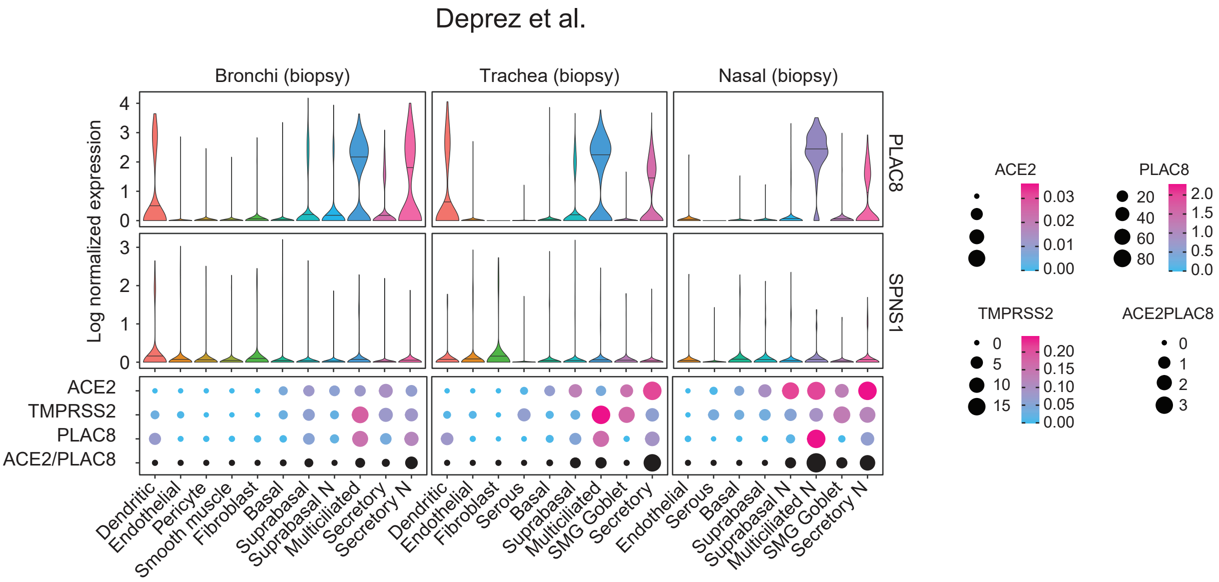

Appendix Figure S3: Expression analyses of *PLAC8*, *ACE2* and *TMPRSS2* as in Figure 4A using the scRNA-Seq data from healthy lung tissues provided by Deprez *et al*.

Appendix Figure S4

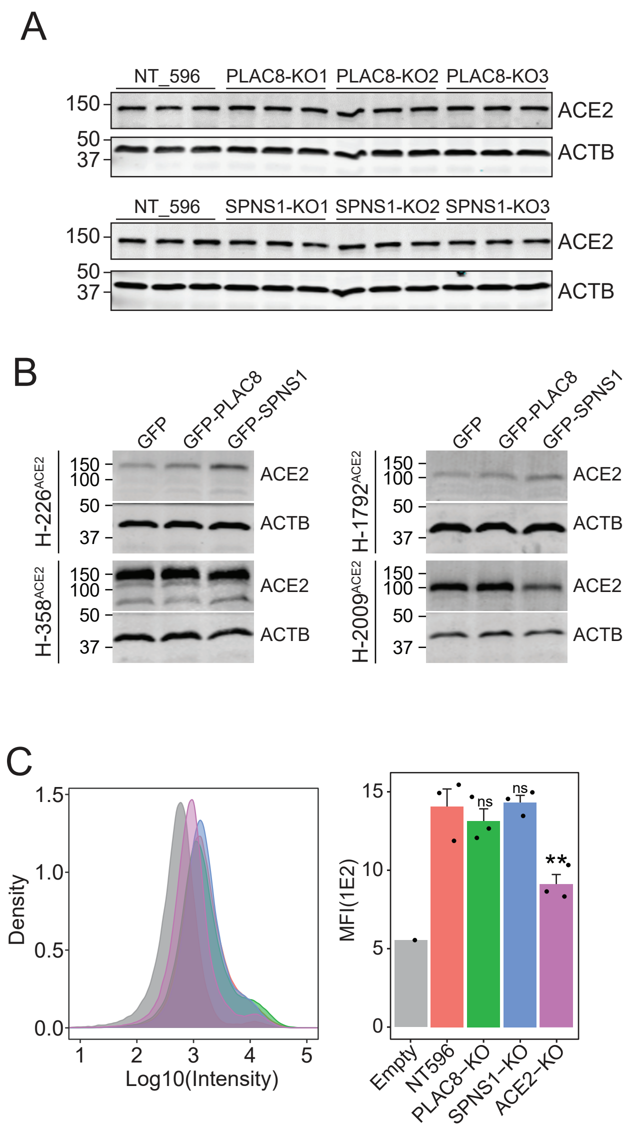

Appendix Figure S4: A) Western-blot analysis of ACE2 protein levels in *PLAC8* and *SPNS1* CRISPR-KO Calu1^{ACE2} cells. NT_596: non-targeting CRISPR vector control cells. Note that ACE2 immunodetection was done on the same blots than in Appendix Figure S1A and therefore the **actin loading control was reused for this figure. B)** Western-blot analysis of ACE2 protein levels in different cancer cell lines that ectopically overexpress PLAC8- or GFP-SPNS1. Actin immunodetection was used as loading control. **C)** Flow cytometry immunofluorescence analysis of surface ACE2 levels in the same cell lines than **(A)**. Calu1^{Empty} (empty vector, without ACE2 overexpression) and *ACE2-KO* Calu1^{ACE2} cells were included as controls. The bar plot indicates the mean and SEM of the median fluorescence intensity values (MFI) in three biological replicates. The significance above each bar represents the t-test p-value between each condition and the CRISPR non-targeting cell line NT596. The histogram plot represents the density of log10 fluorescence intensity values in each condition (replicates were merged to facilitate visualization). Significance: ns, p-value >= 0.05, ** p-value < 0.01.

Appendix Figure S5

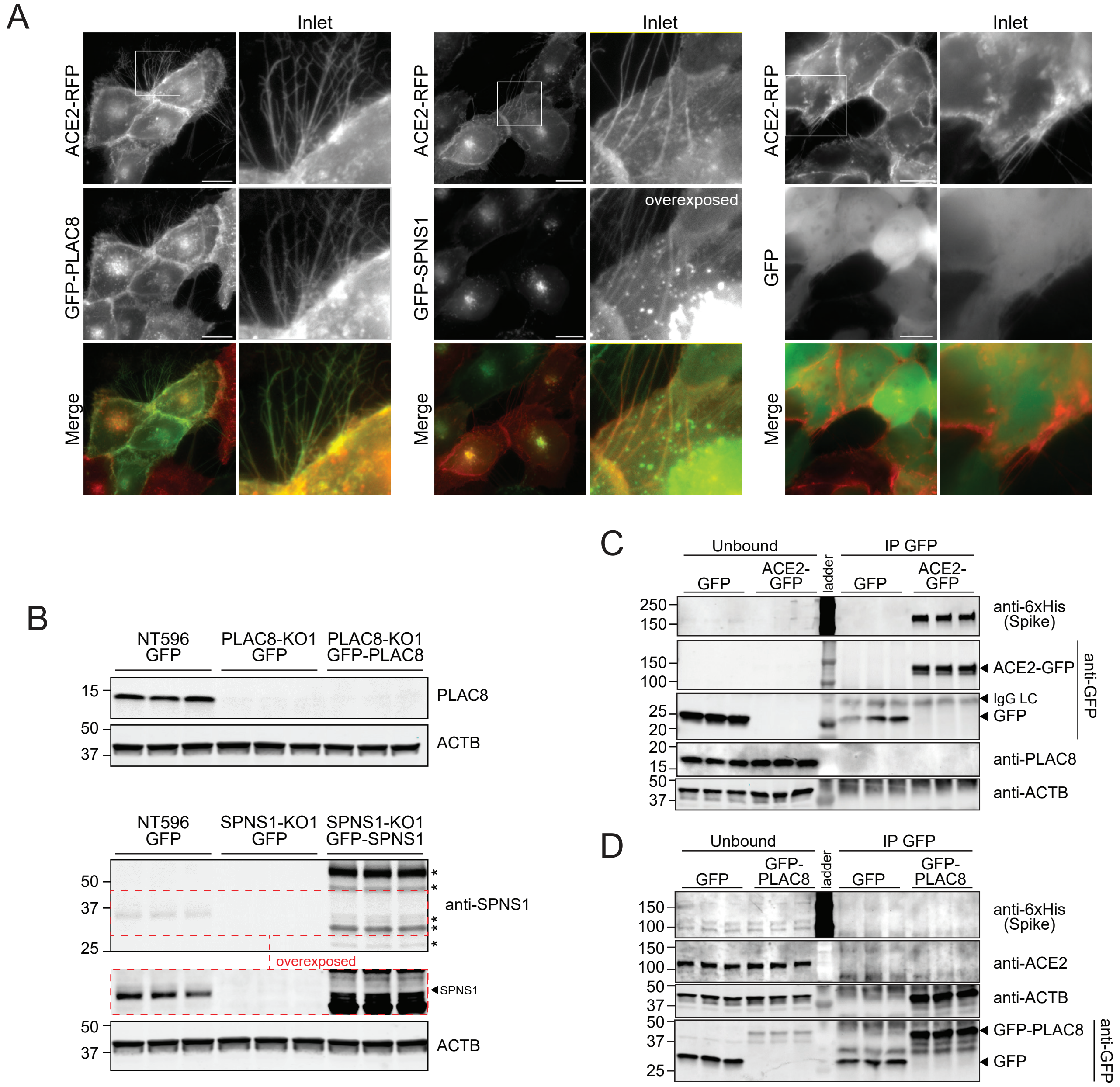

Appendix Figure S5: A) Wide-field live-cell fluorescence imaging of Calu1-ACE2-RFP cells that overexpress GFP-PLAC8 or GFP-SPNS1. Scale bar: 20 µm. Inlets show a magnification of the region defined by the white squares. The inlet for GFP-SPNS1 was overexposed to show the cell surface signal. **B)** Western-blot analysis of endogenous PLAC8 and SPNS1 in *PLAC8* and *SPNS1* CRISPR-KO Calu1-ACE2-RFP cells that overexpress GFP-PLAC8 and GFP-SPNS1, respectively. NT596: non-targeting CRISPR control cells. Actin detection was used as loading control. An overexposed version of SPNS1 blot is shown. Asterisks indicate GFP-SPNS1 bands. **C)** Western-blot detection of SARS-CoV-2 Spike protein (anti-6xHis tag antibody), ACE2-GFP and GFP (anti-GFP antibody), PLAC8 (anti-PLAC8 antibody) and actin (anti-ACTB antibody) in protein extracts from Calu1 cells overexpressing GFP or ACE2-GFP that have been incubated with full-length SARS-CoV-2 Spike recombinant protein and immunoprecipitated using anti-GFP antibodies (IP GFP). The unbound fraction (1/60th of input) was run in parallel to assess the depletion of immunoprecipitated proteins. **D)** Western-blot detection of SARS-CoV-2 Spike protein (anti-6xHis tag antibody), ACE2 (anti-ACE2 antibody), GFP and GFP-PLAC8 (anti-GFP antibody), and actin (anti-ACTB antibody) in protein extracts from *PLAC8-KO* Calu1^{ACE2} cells overexpressing GFP or GFP-PLAC8 that have been incubated with full-length SARS-CoV-2 Spike recombinant protein and immunoprecipitated using anti-GFP antibodies (IP GFP). The unbound fraction (1/60th of input) was run in parallel to assess the depletion of immunoprecipitated proteins. Note that actin labelling was done on the same blot that had been previously incubated with anti-GFP antibody and therefore the GFP-PLAC8 signal is still detected.

Appendix Figure S6

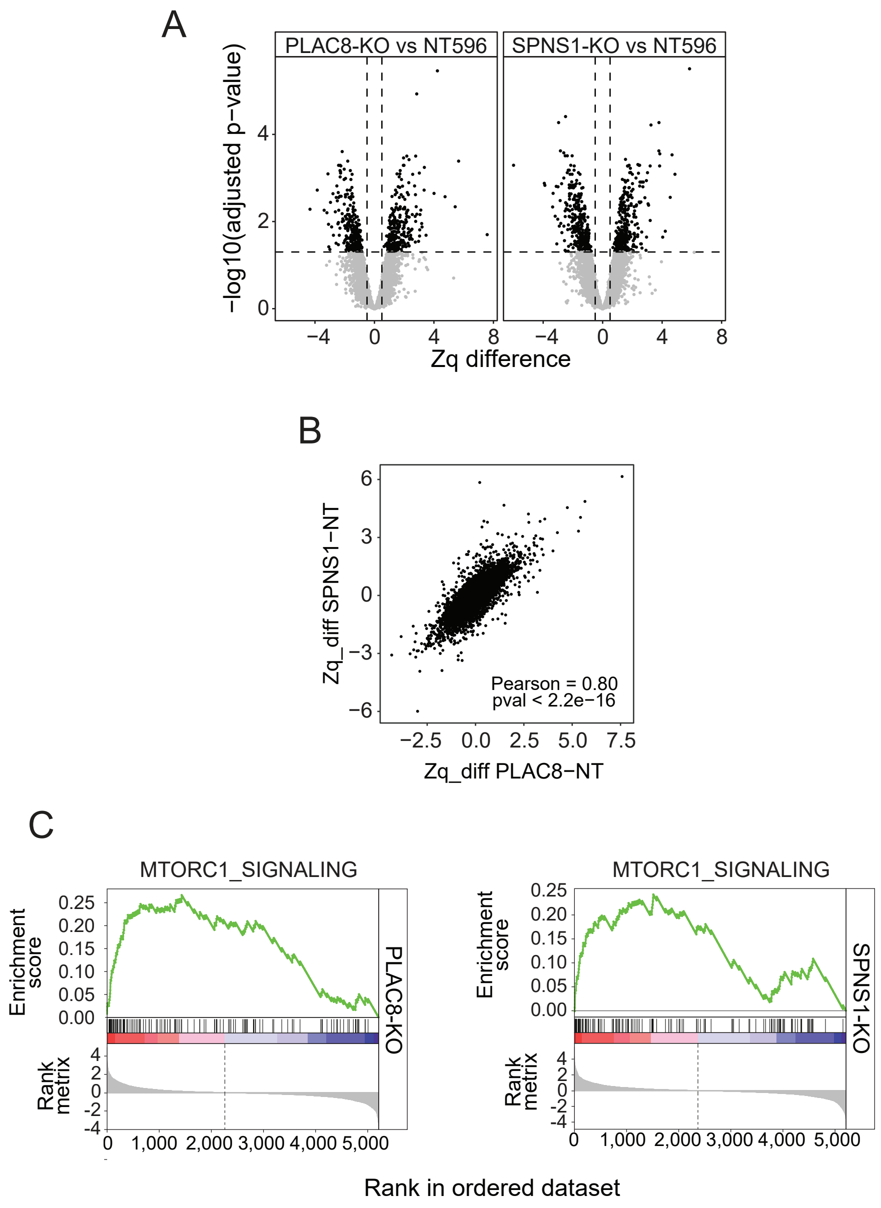

Appendix Figure S6: A) Volcano plot summarizing the differential proteomics expression analyses of *SPNS1-KO* and *PLAC8-KO* Calu1^{ACE2} compared to CRISPR non-targeting Calu1^{ACE2} (NT596). Three biological replicates were used per condition. **B)** Scatter plot showing the correlation between the quantitative protein changes (expressed in Zq values) in *PLAC8-* and *SPNS1-KO* Calu1^{ACE2} cells compared to CRISPR non-targeting control cells (NT596). The Pearson correlation coefficient and test p-value (pval) is indicated within the plot. **C)** GSEA results for the mTORC1_signaling pathway in *PLAC8-* and *SPNS1-KO* Calu1^{ACE2} cells compared to CRISPR non-targeting control cells (NT596).

Appendix Figure S7

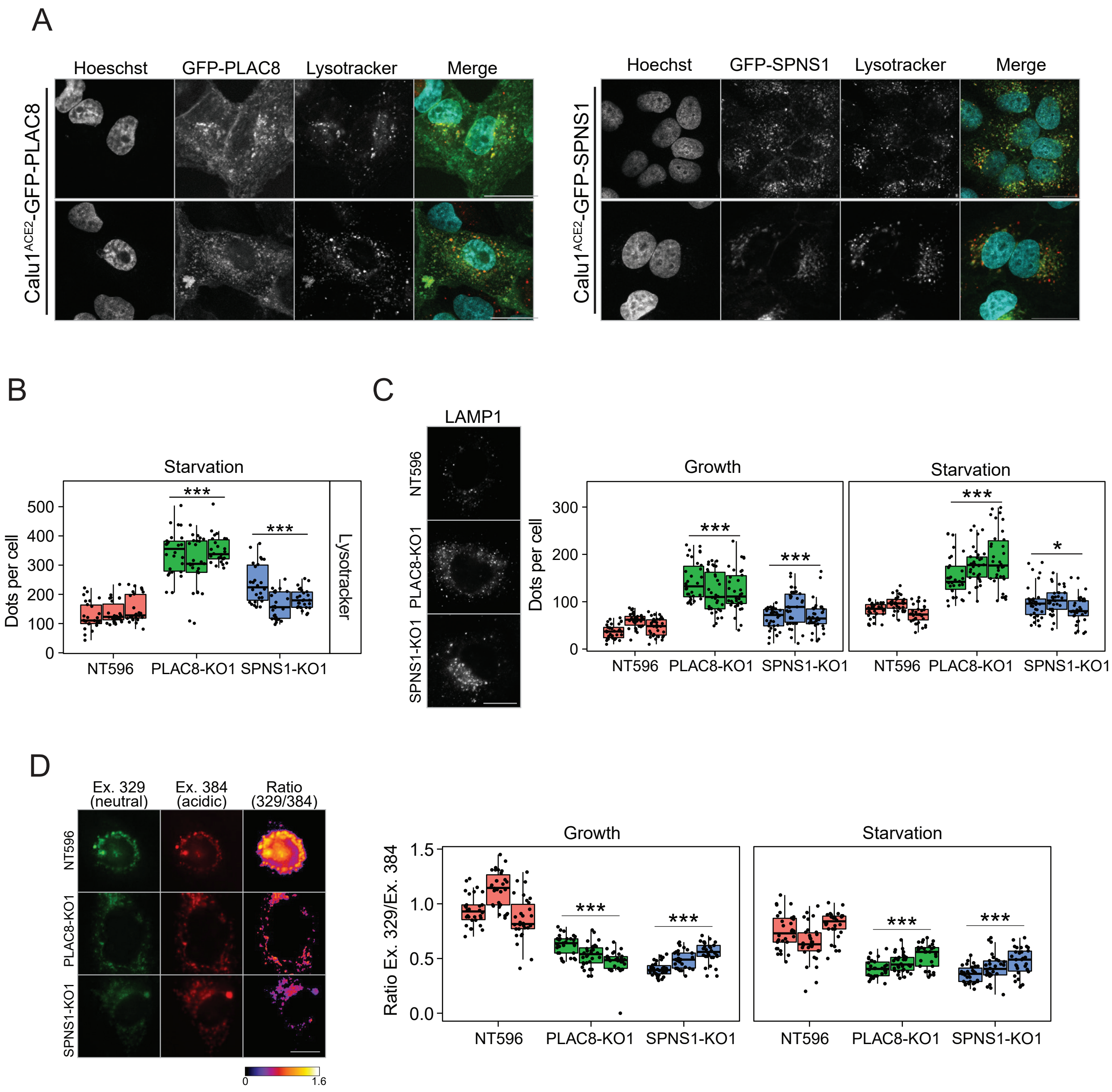

Appendix Figure S7: A) Representative confocal microscopy live-cell images of *GFP-SPNS1* and *GFP-PLAC8* (green) Calu1^{ACE2} cells stained with the lysosomal marker *lysotracker (red)* and the nucleus marker Hoechst 33342 (magenta). Scale bar: 20 µm. **B)** Boxplots showing the number of acidic particles under serum starvation conditions in *PLAC8-* and *SPNS1-KO* Calu1^{ACE2} cells compared to CRISPR non-targeting control cells (NT596) using the live-cell lysotracker staining. **C)** Representative images (top) and quantification (bottom) of the number of lysosomes in *PLAC8-* and *SPNS1-KO* Calu1^{ACE2} cells compared to CRISPR non-targeting control cells (NT596) using immunofluorescence staining of the lysosomal marker LAMP1. **D)** Representative images (left) and quantification (right) of the lysosomal pH in *PLAC8-* and *SPNS1-KO* Calu1^{ACE2} cells compared to CRISPR non-targeting control cells (NT596) using live-cell lysosensor staining. In (B-C), the two samples Wilcoxon Test p-value of the pooled observations relative to NT596 cells is indicated above each condition. Significance: ns, p-value >= 0.05, * p-value < 0.05, ** p-value < 0.01, *** p-value < 0.001. The central band of the boxplots in **B-D** represents the median, the box represents the interquartile range (IQR) between the 25th and 75th percentile, and the whiskers extend from the box to 1.5*IQR. Each dot represents a cell measurement from different microphotographs of a single replicate. Three biological replicates per condition were used. Scale bar: 20 µm

Appendix Figure S8

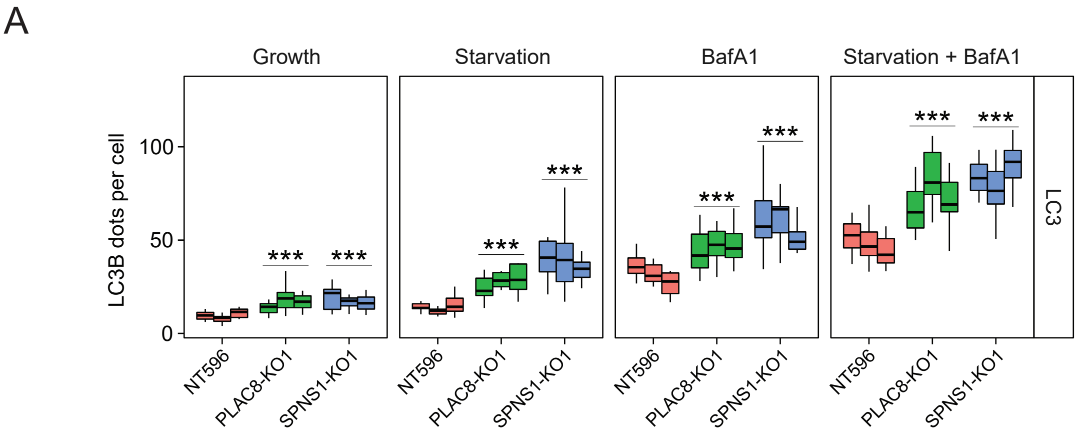

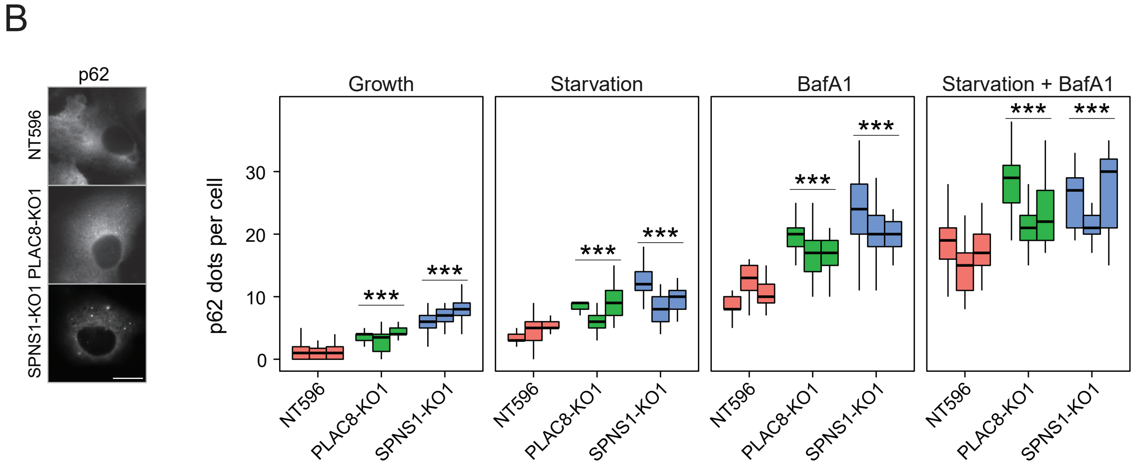

Appendix Figure S8: **A)** Boxplots of the number of autophagosomes in *PLAC8*- and *SPNS1-KO* Calu1^{ACE2} cells compared to CRISPR non-targeting control cells (NT596) using immunofluorescence staining of LC3B. Note that **growth condition corresponds to Figure 6E** and was included here for comparison. **B)** Representative images (left, scale bar: 20 µm) and quantification (right) of the number of p62 puncta in *PLAC8*- and *SPNS1-KO* Calu1^{ACE2} cells compared to CRISPR non-targeting control cells (NT596) using immunofluorescence staining of p62. In all panels, the two samples Wilcoxon Test p-value of the pooled observations relative to NT596 cells is indicated above each condition. Three biological replicates per condition were used and at least 100 cells were analyzed in each sample. The central band of the boxplots represents the median, the box represents the interquartile range (IQR) between the 25th and 75th percentile, and the whiskers extend from the box to 1.5*IQR. Significance: ns, p-value >= 0.05, * p-value < 0.05, ** p-value < 0.01, *** p-value < 0.001.

Appendix Figure S9

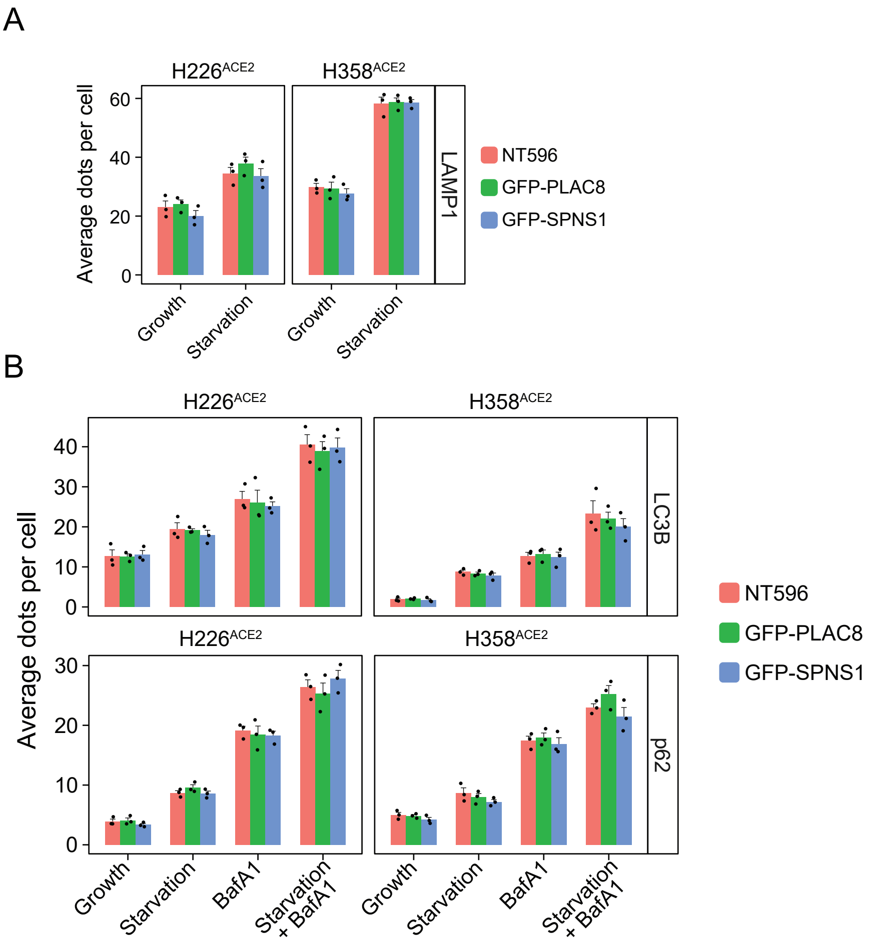

Appendix Figure S9: A) Bar plots showing the average and SEM of the number of lysosomes in three biological replicates under growth and serum starvation conditions in H226^{ACE2} and H358^{ACE2} cells overexpressing GFP-PLAC8 and GFP-SPNS1 using immunofluorescence staining of the lysosomal marker LAMP1. **B)** Bar plots showing the average and SEM of the number of LC3B and p62 particles in three biological replicates under the indicated conditions in H226^{ACE2} and H358^{ACE2} cells overexpressing GFP-PLAC8 and GFP-SPNS1 using immunofluorescence staining of the autophagosome markers LC3B and p62. A total of at least 100 cells were analyzed per sample.

Appendix Table S1

**NGS library preparation**	
NGS1_ATA_fwd	CACGACGCTCTTCCGATCTNNNNNNNNNNATAGCTTTATATATCTTGTGGAAAGGACGAAACACC
NGS1_TAA_fwd	CACGACGCTCTTCCGATCTNNNNNNNNNNTAAGCTTTATATATCTTGTGGAAAGGACGAAACACC
NGS1_GECKO_rev	GTGACTGGAGTTCAGACGTGTGCTCTTCCGATCTCCGACTCGGTGCCACTTTTTCAAG
NGS2_fwd	AATGATACGGCGACCACCGAGATCTACACTCTTTCCCTACACGACGCTCTTCCGATCT
NGS2_rev10	CAAGCAGAAGACGGCATACGAGATAAGCTAGTGACTGGAGTTCAGACGTG
**CRISPR guide cloning**	
ACE2_fw	CACCGATGAGCACCATCTACAGTAC
ACE2_rv	AAACGTACTGTAGATGGTGCTCATC
CCDC53_fw	CACCGCCTCACAAACTGTAGAAAAG
CCDC53_rv	AAACCTTTTCTACAGTTTGTGAGGC
COMMD2_fw	CACCGCACAGCAATCCGCCCAAACT
COMMD2_rv	AAACAGTTTGGGCGGATTGCTGTGC
CTSL1_fw	CACCGGCTTTCAAAACCGTAAGCCC
CTSL1_rv	AAACGGGCTTACGGTTTTGAAAGCC
PLAC8_KO1_fw	CACCGGCCGGACCGGGACCGACTCC
PLAC8_KO1_rv	AAACGGAGTCGGTCCCGGTCCGGCC
PLAC8_KO2_fw	caccgATGAATGAATGCTGTCTGTG
PLAC8_KO2_rv	aaacCACAGACAGCATTCATTCATc
PLAC8_KO3_fw	caccgGACTCACAGGGATGCCATAT
PLAC8_KO3_rv	aaacATATGGCATCCCTGTGAGTCc
SPNS1_KO1_fw	CACCGGGCAATGAGAGTGGGCGCGA
SPNS1_KO1_rv	AAACTCGCGCCCACTCTCATTGCCC
SPNS1_KO2_fw	caccgTCCGCTGGTCGGCCACAAAG
SPNS1_KO2_rv	aaacCTTTGTGGCCGACCAGCGGAc
SPNS1_KO3_fw	caccgAATCGGAAGTATCTCATGTG
SPNS1_KO3_rv	aaacCACATGAGATACTTCCGATTc
VPS26A_fw	CACCGTTCATACCTCAAGCGGACAT
VPS26A_rv	AAACATGTCCGCTTGAGGTATGAAC
VPS29_fw	CACCGCACTGGGGCATATAATGCCT
VPS29_rv	AAACAGGCATTATATGCCCCAGTGC
NT_0596_fw	CACCGAATCGCAGGTATCCCAGAGC
NT_0596_rv	AAACGCTCTGGGATACCTGCGATTC
NT_0764_fw	CACCGCCACGATGCCACCTCATCCC
NT_0764_rv	AAACGGGATGAGGTGGCATCGTGGC
**DNA constructs cloning**	
MCS_f	GGCCGCGAGACGACCGGTGGATCCTCTAGACGTCTCC
MCS_rv	TCGAGGAGACGTCTAGAGGATCCACCGGTCGTCTCGC
SFFV_ACE2	gccgcgagacgaccggtgATGTCAAGCTCTTCCTGGCT
ACE2GFP_SFFV	cgaggagacgtctagaggatcTTACTTGTACAGCTCGTCCA
SFFV_GFP	gccgcgagacgaccggtgATGGTGAGCAAGGGCGAG
RFP_SFFV	cgaggagacgtctagaggatcCTAGTACAGCTCGTCCATGC
SFFV_RFP	gccgcgagacgaccggtgATGGTGTCTAAGGGCGAAGA
SFFV_ACE2	gccgcgagacgaccggtgATGTCAAGCTCTTCCTGGCT
ACE2_3	GGTACCAAAGGAGGTCTGAA
ACE2RFP_5	TTCAGACCTCCTTTGGTACCATGGTGTCTAAGGGCGAAGA

Appendix Table S1: oligonucleotides used for screen NGS library preparation, DNA construct generation and CRISPR guides used in this work.

Appendix Table S2

Antibodies and recombinant proteins	Type	Source	Reference
Anti-LAMP1 antibody - Lysosome Marker	Primary antibody	Abcam	ab24170
Anti-TMPRSS2 antibody [EPR3861]	Primary antibody	Abcam	ab92323
SQSTM1 monoclonal antibody (M01), clone 2C11	Primary antibody	Abnova	H00008878-M01
anti-SARS-CoV / SARS-CoV-2 nucleocapsid protein antibody [SQab20177]	Primary antibody	Arigo Biolaboratories	ARG66735
6x-His Tag Monoclonal Antibody (HIS.H8)	Primary antibody	Invitrogen™	MA1-21315
ACE2 Recombinant Rabbit Monoclonal Antibody (SN0754)	Primary antibody	Invitrogen™	MA5-32307
PLAC8 Rabbit anti-Human, Polyclonal	Primary antibody	Invitrogen™	15737051
SPNS1 Rabbit anti-Human, Polyclonal	Primary antibody	Invitrogen™	15757661
LC3B Antibody - BSA Free	Primary antibody	Novus Biologicals	NB600-1384
Human/Rat/Hamster ACE-2 Antibody (Clone 535919)	Primary antibody	R&D Systems	MAB9332
Monoclonal Anti-β-Actin antibody produced in mouse, clone AC-15, ascites fluid	Primary antibody	Sigma-Aldrich	A5441
Living Colors® Full-Length GFP Polyclonal Antibody	Primary antibody	Takara	632592
Transferrin From Human Serum, Alexa Fluor™ 647 Conjugate	Recombinant protein	Invitrogen™	T23366
Recombinant SARS-CoV-2 Spike (GCN4-IZ) His Protein, CF	Recombinant protein	R&D systems	10561-CV
Recombinant SARS-CoV-2 Spike RBD Alexa Fluor® 647 Protein	Recombinant protein	R&D systems	AFR10500
Goat anti-Human IgG (H+L) Cross-Adsorbed Secondary Antibody, Alexa Fluor™ 568	Secondary antibody	Invitrogen™	A-21090
IRDye® 680RD Goat anti-Mouse IgG Secondary Antibody	Secondary antibody	LI-COR Biosciences	926-68070
IRDye® 680RD Goat anti-Rabbit IgG Secondary Antibody	Secondary antibody	LI-COR Biosciences	926-68071
IRDye® 800CW Goat anti-Mouse IgG Secondary Antibody	Secondary antibody	LI-COR Biosciences	926-32210
IRDye® 800CW Goat anti-Rabbit IgG Secondary Antibody	Secondary antibody	LI-COR Biosciences	926-32211
Anti-Human IgG (Fc specific)−FITC antibody produced in goat	Secondary antibody	Sigma-Aldrich	F9512
Peroxidase AffiniPure Goat Anti-Mouse IgG (H+L)	Secondary antibody	Jackson Immunoresearch	115-035-062
Anti-rabbit IgG, HRP-linked Antibody	Secondary antibody	Cell Signaling Technol	7074

Appendix Table S2: antibodies and recombinant proteins used in this work.
