# Supplementary figures and images for: Autophagy‐linked plasma and lysosomal membrane protein PLAC8 is a key host factor for SARS‐CoV‐2 entry into human cells
Source: EMBO J. 2022 Oct 4;41(21):e110727. doi: 10.15252/embj.2022110727 (PMC9627672; doi:10.15252/embj.2022110727)

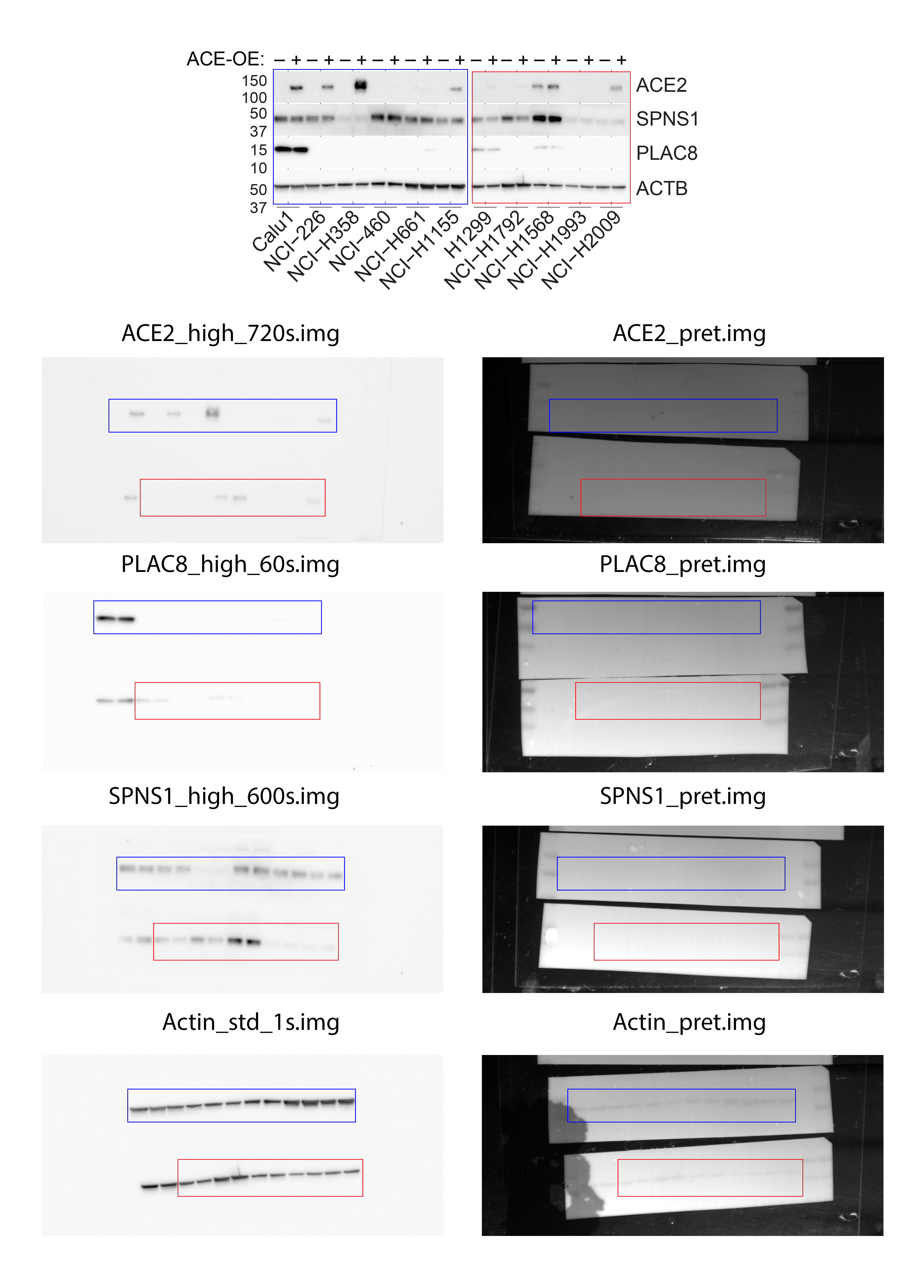

Supplement: Supplementary file 6 — Source Data for Figure 1 [file EMBJ-41-e110727-s001.zip › Figure1_source_data.JPG]
