# Supplementary material for: Autophagy‐linked plasma and lysosomal membrane protein PLAC8 is a key host factor for SARS‐CoV‐2 entry into human cells
Source: EMBO J. 2022 Oct 4;41(21):e110727. doi: 10.15252/embj.2022110727 (PMC9627672; doi:10.15252/embj.2022110727)
Supplement: Supplementary file 10 [file EMBJ-41-e110727-s007.pdf]

THE EMBO JOURNAL

Autophagy-linked plasma and lysosomal membrane protein PLAC8 is a key host factor for SARS-CoV-2 entry into human cells

Alejandro Ugalde, Gabriel Bretones, David Rodríguez, Victor Quesada, Francisco Llorente, Raul Fernández-Delgado, Miguel Ángel Jiménez-Clavero, Jesús Vázquez, Enrique Calvo, Isaac Tamargo-Gómez, GUILLERMO MARIÑO GARCÍA, David Roiz-Valle, Daniel Maeso, Miguel Araujo Voces, Yaiza Español, Carles Barceló, Jose Freije, Alejandro López-Soto, and Carlos Lopez-Otin
DOI: 10.15252/embj.2022110727
Corresponding author(s): Alejandro Ugalde (pineiroalejandro@uniovi.es) , Carlos Lopez-Otin (clo@uniovi.es), Gabriel Bretones (bretonesgabriel@uniovi.es)

Review Timeline:

Submission Date:	20th Jan 22
Editorial Decision:	3rd Mar 22
Revision Received:	23rd Jun 22
Editorial Decision:	5th Aug 22
Revision Received:	30th Aug 22
Accepted:	5th Sep 22

Editor: Karin Dumstrei

Transaction Report:

(Note: With the exception of the correction of typographical or spelling errors that could be a source of ambiguity, letters and reports are not edited. Depending on transfer agreements, referee reports obtained elsewhere may or may not be included in this compilation. Referee reports are anonymous unless the Referee chooses to sign their reports.)

1st Editorial Decision 3rd Mar 2022

Dear Dr. Ugalde,

Thank you for submitting your manuscript to The EMBO Journal. Your study has now been seen by two referees and their comments are provided below. I am still waiting for the input from a third referee who had agreed to review the paper, but at this stage I don't think that I will receive the comments. I will therefore go ahead with the two reports on the paper.

As you can see below, the referees appreciate the analysis but also find that further analysis would be needed to consider publication here. Should you be able to address the raised concerns then I would be interested I considering a revised version. The points raised are clearly indicated below. We would also need some insight into the how PLAC8/SPSN1 promotes SARS-CoV-2 entry. We don't need the full mechanism, but some more understanding. You don't need to do any analysis with SARS and MERS (Point #6 ref #2).

I think it would be helpful to discuss the raised points further and I am available to do so via email or video.

When preparing your letter of response to the referees' comments, please bear in mind that this will form part of the Review Process File, and will therefore be available online to the community. For more details on our Transparent Editorial Process, please visit our website: https://www.embopress.org/page/journal/14602075/authorguide#transparentprocess

I thank you for the opportunity to consider your work for publication. I look forward to discussing your revisions further

with best wishes

Karin

Karin Dumstrei, PhD
Senior Editor
The EMBO Journal

Instructions for preparing your revised manuscript:

Please find attached a PDF with helpful tips on how to prepare the revised version

Guide For Authors: https://www.embopress.org/page/journal/14602075/authorguide

I realise that it is difficult to revise to a specific deadline. In the interest of protecting the conceptual advance provided by the work, we recommend a revision within 3 months (1st Jun 2022). If you need further time I can easily extend the revision time.

Use the link below to submit your revision:

https://emboj.msubmit.net/cgi-bin/main.plex

Referee #1:

In this manuscript, the authors performed a CRISPR screen in a lung cancer cell line to identify factors that affect cell infection with SARS-CoV-2 Spike-pseudotyped lentivirus. They newly identified SPNS1 and PLAC8 as cellular proteins that increase Spike-dependent infection of pseudotyped virus and confirmed this with authentic SARS-CoV-2 infection. These conclusions are overall well supported with both gain and loss of function experiments in multiple lung cell lines. They went on to analyze single cell RNA sequencing results to show that SPNS1 is widely expressed while PLAC8 is more selectively expressed in cell types that often co-express the virus receptor ACE2. They then performed quantitative proteomics on WT cells compared to PLAC8 or SPNS1 KO cells. Pathway analysis of differentially expressed proteins implicated a role for these proteins in autophagolysosome pathways and metabolic processes. Involvement of the proteins in controlling lysosome numbers and acidity in cells was confirmed. Overall, the experiments are well done, the provided data are convincing, and the discovery of these new factors in SARS-CoV-2 infection is an important addition to our fundamental knowledge of SARS-CoV-2 cellular entry.

Minor concerns:

1. The authors ignore the significant body of literature that suggests that SARS-CoV-2 enters cells via fusion at the plasma

membrane (rather than via endocytosis), particularly in lung cells. It would be interesting to know if PLAC8 affects infection of Calu3 cells or primary human lung cells that have been shown to primarily support the plasma membrane pathway of SARS-CoV-2 entry. Discussion of the basal levels of PLAC8 and SPNS1 in A549 and Calu3 cells would also be useful since these cell lines have been so extensively used in other SARS-CoV-2 publications.
2. The authors may also consider discussing whether their screen uncovered known restriction factors for SARS-CoV-2, such as Ly6E or CH25H, or also whether their data give insights into the controversial role of IFITM proteins in SARS-CoV-2 cell entry.
3. While the authors show that PLAC8/SPSN1 promote SARS-CoV-2 entry and that these proteins also affect autolysosomes, a cause-and-effect mechanism linking these two observed phenomena was not provided. Thus, it remains unclear how these factors enhance virus entry.
4. The supplementary tables need legends to make them interpretable.
5. Some of the asterisks indicating significance on various graphs throughout the manuscript are hard to distinguish from data points.

Referee #2:

Ugalde et al. reported a novel entry factor for SARS-CoV-2. This factor, PLAC8, was identified through a genome-wide CRISPR screen in a human lung cancer cell line. The authors provided evidence to support the function of PLAC8 mediating SARS-CoV-2 entry. The RNA-seq analysis also confirmed the expression of PLAC8 in SARS-CoV-2 primary target cells in the human respiratory tract. Further, results suggested autophagy and lysosomal function may be regulated by PLCA8 in SARS-CoV-2 entry. The overall finding is potentially interesting. However, several important questions were not addressed which compromise the soundness and significance of this manuscript. Since PLAC8 is involved in multiple types of human tumors including lung cancer, PLAC8 role for viral infections in the lung cancer line may not be relevant unless confirmed in primary cells.

Major concerns:
1. Which step(s) of SARS-CoV-2 entry is affected in PLCA8/SPNS1 KO? Binding or internalization? The answer to this question will help understand the mechanisms of these two factors.
2. For SARS-CoV-2 entry, a very important factor, TMPRSS2, should be considered. This factor determines the entry pattern of SARS-CoV-2 in humans. The primary target cells always express TMPRSS2 and SARS-CoV-2 prefers to directly fuse with cell membrane but not through endocytosis. However, in the whole study, all the cell lines were lacking TMPRSS2 which might cause artificial effects. Therefore, observed effects should be confirmed in TMPRSS2+ cells.
3. Considering PLAC8 is also expressed in the cell membrane, it may provide a therapeutic target. Therefore, blocking antibody or purified PLAC8 protein could be used to block SARS-CoV-2 entry. This experiment will further validate the function of PLAC8.
4. Does PLAC8 directly interact with Spike? Many viral receptors/co-receptors interact with viral spike/glycoprotein to promote entry.
5. Although the authors put a lot of effort into RNA-seq data mining and found the expression pattern of PLCA8 matched the tropism of SARS-CoV-2, however, it is still essential to validate the function of PLCA8 in primary cells such as bronchial epithelial cells or nasal cells from humans.
6. How about other human coronaviruses like SARS-CoV-1, MERS-CoV? These viruses may also utilize these factors.
7. Fig 5F is not convincing because the ratio panel (merged images) is highly saturated as compared to their respective individual images.
8. levels of LC3 and p62 are different in Calu1ACE2 and H1299ACE2 cell lines and not much significant after knocking down of PLAC8?

Minor points:
1. Figure 3A. The y axis title should be "ratio", not "percentage"
2. Autophagy and lysosomal function are involved in SARS-CoV-2 replication and release but not entry. Not sure this figure could link the function of the two factors and viral entry.

Referee #3:

To identify targetable host factors for SARS CoV2 infections Ugalde et al performed a Crispr screen in human Calu1 lung cancer cells (overexpressing ACE2) using pseudotyped lentivirus. The authors pulled out two new factors, namely SPNS1 and PLAC8, that in loss of function experiments were confirmed to be involved in viral entry, including infections with the fully infectious SARS-CoV-2 virus. PLAC8 is highly expressed in ciliated and secretory cells from the respiratory tract and in gut enterocytes and its overexpression facilitates viral entry. Mechanistically the authors propose that SPNS1 and PLAC8 affect viral entry through regulation of autophagy and lysosomal function.

Though the proposed pathway is not novel and many questions remain on detailed mechanisms, this is certainly an interesting set of well conducted experiments that identify novel regulators of SARS-CoV-2.

I am not expert on Crispr screening (genome wide p values, proper controls, library used etc.) but the screen was done in any case to identify novel players in viral entry. The validation of the SPNS1 and PLAC8 knockout clones and their rescues, in particular, are very convincing. One question: in S. Fig. 1C, was the ACE2 clone (I assume the red bar is an ace2 wild type parent clone and the green bar an ace2 KO clone, would be good to describe this better in the figure legend) an incomplete knockout clone considering that viral entry is only reduced by about 40%.

For reporting the data in Fig. 2B etc, "Bars represent the average and standard error of the mean (SEM) of the percentage of infected cells in each condition (three replicates), normalized to the non-targeting control cell line NT596." Was the intensity of the zsGREEN signal also reduced in the remaining cells that were infected or is this an all or none phenomenon? If the latter is true, how do you explain this - incomplete knockouts? Please discuss this, unless I missed it somehow.

As for the SARS-CoV2 infections in Figure 3, it would be important to also assess viral RNA in addition to "immunofluorescence of nucleocapsid (N) protein 24 h post infection (p.i.)." After all, if the hypothesis of the authors is correct then there should be also a marked decrease in viral replication.

We would like to thank the referees for providing constructive and valuable suggestions to improve and strengthen our manuscript. We have used these comments as the basis for revising the manuscript. We have comprehensively addressed all concerns and provide below point-by-point list of responses to the reviewers' specific comments. Modifications of the original main text are marked with Microsoft Word change tracking in the revised manuscript.

Referee #1

In this manuscript, the authors performed a CRISPR screen in a lung cancer cell line to identify factors that affect cell infection with SARS-CoV-2 Spike-pseudotyped lentivirus. They newly identified SPNS1 and PLAC8 as cellular proteins that increase Spike-dependent infection of pseudotyped virus and confirmed this with authentic SARS-CoV-2 infection. These conclusions are overall well supported with both gain and loss of function experiments in multiple lung cell lines. They went on to analyze single cell RNA sequencing results to show that SPNS1 is widely expressed while PLAC8 is more selectively expressed in cell types that often co-express the virus receptor ACE2. They then performed quantitative proteomics on WT cells compared to PLAC8 or SPNS1 KO cells. Pathway analysis of differentially expressed proteins implicated a role for these proteins in autophagolysosome pathways and metabolic processes. Involvement of the proteins in controlling lysosome numbers and acidity in cells was confirmed. Overall, the experiments are well done, the provided data are convincing, and the discovery of these new factors in SARS-CoV-2 infection is an important addition to our fundamental knowledge of SARS-CoV-2 cellular entry.

Minor concerns:

1. The authors ignore the significant body of literature that suggests that SARS-CoV-2 enters cells via fusion at the plasma membrane (rather than via endocytosis), particularly in lung cells. It would be interesting to know if PLAC8 affects infection of Calu3 cells or primary human lung cells that have been shown to primarily support the plasma membrane pathway of SARS-CoV-2 entry. Discussion of the basal levels of PLAC8 and SPNS1 in A549 and Calu3 cells would also be useful since these cell lines have been so extensively used in other SARS-CoV-2 publications.

We tested these cell lines in an initial round for suitable cellular models to study SARS-CoV-2. However, we discarded them because of the low infection efficiency using Spike-pseudotyped virus, even upon ACE2 overexpression. Nonetheless, as per your request, we have generated ACE2-RFP, GFP-PLAC8 and GFP-SPNS1 overexpression cell lines from Calu3, A549, and VeroE6 cells (another popular cell line in SARS-CoV-2 research from African Green Monkey) and tested them for infection efficiency using our flow-cytometry-based assays (see attached figure 1). These experiments produced again low ratios of infection in the parental cell lines, and ACE2-RFP overexpression failed to increase it. In contrast, the same batch of viruses yielded a 20% infection rate in $Calu1^{ACE2}$ cells. Notably, the infection efficiency with VSVG-typed virus is also very low in these cell lines. However, despite the overall low infection rate, GFP-PLAC8 overexpression resulted in a highly reproducible 3- to 6-fold increase in infection efficiency with S-typed viruses in A549 and VeroE6 cells. Interestingly, GFP-PLAC8 overexpressing VeroE6 cells without ACE2 overexpression also experienced that increase, probably because these cells have high basal expression of ACE2.

We do not know the reasons why Calu3 and A549 cells produce overall low infection rate in our infection assays with pseudotyped viruses. The main cell lines used in our work, as well as Calu3 and A549 were tested by STR genotyping and their identities were confirmed. There is a vast abundance of SARS-CoV-2 research literature; a detailed analysis of the experimental procedures used in other works that employed these cells might identify the origin of these discrepancies. We would like to point out that we used flow-cytometry as readout of infection, which allows to calculate the percentage of

infection cells, in contrast to luciferase reporters that yield a relative mean measurement of the whole population. A possible explanation could also be a different infection susceptibility to pseudotyped lentiviruses and full SARS-CoV-2 viruses. For example, plain VeroE6 cells were included as a control in our experiments with full SARS-CoV-2 viruses and showed the highest ratio of infection (around 10% more than Calu1^{ACE2} cells), while their infection susceptibility to Spike- and VSVG-typed lentiviruses was very low (attached figure 1).

Nonetheless, we would like to point out that all the cellular models (up to 8 different cell lines) are lung cancer lines, like Calu3 or A549, and they have similar levels of TMPRSS2 expression (Figure S2B in the revised manuscript, see point 2 from referee 2). The fact that GFP-PLAC8 overexpression in the attached experiment increases the infection rate of A549 and VeroE6 cells using Spike-typed lentiviruses support our conclusions and extend them to another primate species.

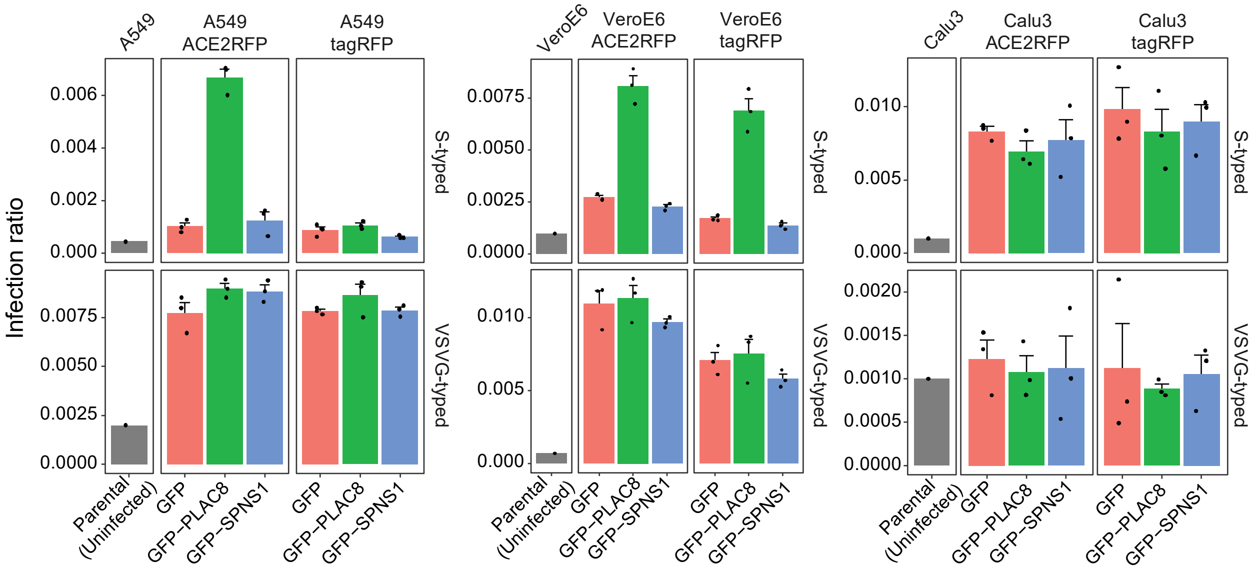

Figure 1: infection susceptibility of Calu3, A549 and VeroE6 cell with tagRFP or ACE2-RFP overexpression and GFP, GFP-PLAC8 or GFP-SPNS1 overexpression using Spike-typed (S-typed) or VSVG-typed lentiviruses. Bars represent the mean and SEM of three biological replicates.

2. The authors may also consider discussing whether their screen uncovered known restriction factors for SARS-CoV-2, such as Ly6E or CH25H, or also whether their data give insights into the controversial role of IFITM proteins in SARS-CoV-2 cell entry.

We are sorry if we missed some relevant SARS-CoV-2 host factors in our discussion. The SARS-CoV-2 literature grows very fast, and it is difficult to be up to date of all relevant discoveries. Ly6E ranks as the 62nd most enriched knock-out gene in our screen, although it does not reach significance after multiple test correction. This observation would agree with the reported role of Ly6E in preventing SARS-CoV-2 membrane fusion. However, we do not see any change in the abundance of CRISPR guides against CH25H or IFITM proteins in our screen results. Nonetheless, Table S1 shows the screen statistics for every gene in our CRISPR-Cas9 screen to allow inspection of any gene of interest. Related to your point, we have included a short sentence in the discussion to point out that PLSCR1, a putative PLAC8 inhibitor, ranks 5th in the list of most enriched knock-out genes in our screen.

3. While the authors show that PLAC8/SPSN1 promote SARS-CoV-2 entry and that these proteins also affect autolysosomes, a cause-and-effect mechanism linking these two observed phenomena was not provided. Thus, it remains unclear how these factors enhance virus entry.

In the revised manuscript, we have performed an extensive characterization of autophagy using more suitable techniques and experiments. We have replaced the Western-blot analyses of the original manuscript with a more complete immunofluorescence study of LC3B and p62, and autophagic flux analyses with the reporter GFP-LC3-RFP-LC3ΔG (Figure 6C-F, S7 and S8 in the revised manuscript). In addition, we have extended these studies to cell lines with GFP-PLAC8 and GFP-SPNS1 overexpression. This set of experiments have helped us to reach a more detailed picture of the role of the autophagolysosomal alterations upon PLAC8 and SPNS1 gain- and loss-of-function in the context of SARS-CoV-2 infection. All these new developments have been discussed in the revised manuscript.

4. The supplementary tables need legends to make them interpretable.

This has been fixed in our revised manuscript.

5. Some of the asterisks indicating significance on various graphs throughout the manuscript are hard to distinguish from data points.

Thank you for your suggestion. We have increased the size of the asterisks in our revised manuscript to make them more clear.

Referee #2

Ugalde et al. reported a novel entry factor for SARS-CoV-2. This factor, PLAC8, was identified through a genome-wide CRISPR screen in a human lung cancer cell line. The authors provided evidence to support the function of PLAC8 mediating SARS-CoV-2 entry. The RNA-seq analysis also confirmed the expression of PLAC8 in SARS-CoV-2 primary target cells in the human respiratory tract. Further, results suggested autophagy and lysosomal function may be regulated by PLCA8 in SARS-CoV-2 entry. The overall finding is potentially interesting. However, several important questions were not addressed which compromise the soundness and significance of this manuscript. Since PLAC8 is involved in multiple types of human tumors including lung cancer, PLAC8 role for viral infections in the lung cancer line may not be relevant unless confirmed in primary cells.

Major concerns:

1. Which step(s) of SARS-CoV-2 entry is affected in PLCA8/SPNS1 KO? Binding or internalization? The answer to this question will help understand the mechanisms of these two factors.

Most of our efforts in this round of revision were focused on trying to elucidate the molecular mechanisms underlying PLAC8 and SPNS1 function as SARS-CoV-2 host factors. We carried out binding and endocytosis experiments using recombinant SARS-CoV-2 RBD Spike protein (Figure 5A-C in the revised manuscript). These experiments suggest that these two processes are not affected by loss-of-function of PLAC8 and SPNS1. Likewise, experiments with transferrin, which uses a different receptor, did not yield differences in the endocytosis rate upon loss-of-function of these two novel host factors. However, confocal microscopy analyses demonstrate that endosomes containing endocytosed Spike-RBD colocalize with vesicles labelled with ACE2-RFP, GFP-PLAC8 and GFP-SPNS1 (Figure 5D-E), which provides molecular evidence of their functional relationship. In this revised manuscript we have also performed a detailed study of the autophagolysosomal compartment (Figure 6C-F and S7-9 in the revised manuscript). Loss-of-function of PLAC8 and SPNS1 increases autophagic activity and produce an expansion and acidification of lysosomes. Despite their alterations, lysosomes seem to be functional, since autophagic flux experiments show no block in autophagy and the uptake and degradation of fluorogenic BSA is not affected. These experiments clearly indicate that PLAC8 and SPNS1 are molecularly connected to autophagolysosomal homeostasis. However, the observation that GFP-PLAC8 and GFP-SPNS1 overexpression does not affect this compartment in cell lines that experience increased infection susceptibility to SARS-CoV-2 does not support a causal relationship.

Altogether, we think that our revised manuscript provides more clues about the molecular function of PLAC8 and SPNS1 in the context of SARS-CoV-2 infection, although we could not reach a fully understanding of the exact molecular mechanisms. All these new observations have been discussed in the revised manuscript more extensively.

2. For SARS-CoV-2 entry, a very important factor, TMPRSS2, should be considered. This factor determines the entry pattern of SARS-CoV-2 in humans. The primary target cells always express TMPRSS2 and SARS-CoV-2 prefers to directly fuse with cell membrane but not through endocytosis. However, in the whole study, all the cell lines were lacking TMPRSS2 which might cause artificial effects. Therefore, observed effects should be confirmed in TMPRSS2+ cells.

In our revised manuscript, we have included a Western-blot of TMPRSS2 in our main cellular models as well as in Calu3, A549 and VeroE6 cells for comparison (see minor point 1 from referee 1). As you can see in Figure S2B in our revised manuscript, TMPRSS2 shows an overall ubiquitous pattern of expression

in all tested cell lines. These results differ from those of Koch et al (EMBOJ, 40: e107821, 2021), where they see very high expression of TMPRSS2 in Calu3 compared to VeroE6 and A549. We would like to point out that we employed the same antibody (Abcam ab92323) and that the main cell lines used in our work have been confirmed by STR genotyping. It might be possible that culture conditions or differences in experimental procedures explain this discrepancy. Nonetheless, in response to point 1 from reviewer 1 we have tested the infection efficiency of these 3 cell lines and found that GFP-PLAC8 overexpression boosts infection with Spike-typed lentiviruses both in A549 and VeroE6 ($TMPRSS^{-}$ in Koch et al.).

3. Considering PLAC8 is also expressed in the cell membrane, it may provide a therapeutic target. Therefore, blocking antibody or purified PLAC8 protein could be used to block SARS-CoV-2 entry. This experiment will further validate the function of PLAC8.

We had also considered to explore this point as a potential therapeutic treatment. However, a previous study clearly demonstrates that PLAC8 localizes to the inner plasma membrane (Kaistha et al, Cancer Res (2016) 76 (1): 96–107), which dissuaded us from exploring this intervention. In addition, our revised manuscript now shows that binding and internalization of recombinant Spike is not affected by PLAC8 loss-of-function (Figure 5 in the revised manuscript). Likewise, immunoprecipitation experiments of ACE2-GFP and GFP-PLAC8 in protein extracts that have been incubated with recombinant Spike protein did not support a direct interaction of PLAC8 with ACE2 or Spike protein (Figure S5C-D in the revised manuscript). All these observations rule out that anti-PLAC8 antibody treatment would influence SARS-CoV-2 infection.

4. Does PLAC8 directly interact with Spike? Many viral receptors/co-receptors interact with viral spike/glycoprotein to promote entry.

As mentioned in the answer to your previous question, we have included immunoprecipitations experiments in the revised manuscript (Figure S5C-D in the revised manuscript). Specifically, protein extracts from Calu1 cells that overexpress either ACE2-GFP or GFP-PLAC8 were incubated with recombinant Spike and subjected to immunoprecipitation with anti-GFP antibodies. While ACE2-GFP immunoprecipitation clearly brought down Spike protein, GFP-PLAC8 immunoprecipitation failed to retrieve recombinant Spike or ACE2, suggesting that PLAC8 does not function as an alternative receptor (also discarded by its inner membrane localization, see answer to previous point) and does not interact directly with ACE2. However, as discussed in the manuscript, it is possible that our immunoprecipitation experiments fail to detect weak or context-specific interactions.

5. Although the authors put a lot of effort into RNA-seq data mining and found the expression pattern of PLCA8 matched the tropism of SARS-CoV-2, however, it is still essential to validate the function of PLCA8 in primary cells such as bronchial epithelial cells or nasal cells from humans.

During this round of revision, we have tried to extend our observations to primary cells. Thus, we purchased HBEC cells and tried to perform functional studies. Unfortunately, these experiments take longer and are more complex than we anticipated. In addition to a delivery delay, these cells grow very slow and were very sensitive to antibiotics selection when trying to generate PLAC8 and SPNS1 gain- and loss-of-function models in a pilot experiment. Likewise, we attempted in parallel to use human colon

organoids, but our lack of experience with this model precluded us to get results during this revision. We agree with you that a validation in human primary lung cells would further support our conclusions. However, we do not think this is an essential experiment for this manuscript. We have employed up to 8 different lung cancer cell lines and, in response to reviewer 1 point 1, we have also tested A549 and VeroE6 cells and confirmed our results about PLAC8. Moreover, during this round a revision another genome-wide CRISPR screen (in human hepatoma HuH7.5 cells) has reported that PLAC8 is a key host factor for the related coronavirus SADS-CoV (Tse et al., PNAS 2022 May 3;119(18):e2118126119).

6. How about other human coronaviruses like SARS-CoV-1, MERS-CoV? These viruses may also utilize these factors.

Although we agree with you that this would be an interesting point to test, when balancing the difficulty of the experiment and the relevance of the results, we decided to discard it. SARS-CoV-1 and MERS-CoV have very low population incidence and finding viral samples, obtaining permissions and access to an authorized laboratory would be very time and cost consuming. Moreover, new cellular models overexpressing the MERS-CoV receptor (DDP4) would be required. However, it is very likely that PLAC8 would also be relevant in SARS-CoV-1 and MERS-CoV infection, since a recent report has also identified PLAC8 as a main host factor for the related coronavirus SADS-CoV (Tse et al., PNAS 2022 May 3;119(18):e2118126119). Related to your question, a pilot experiment of some SARS-CoV-2 variants in H226-ACE2-RFP cells shows that GFP-PLAC8 overexpression boosts infection efficiency in all variants tested (see attached Figure 2), using our flow-cytometry based assays.

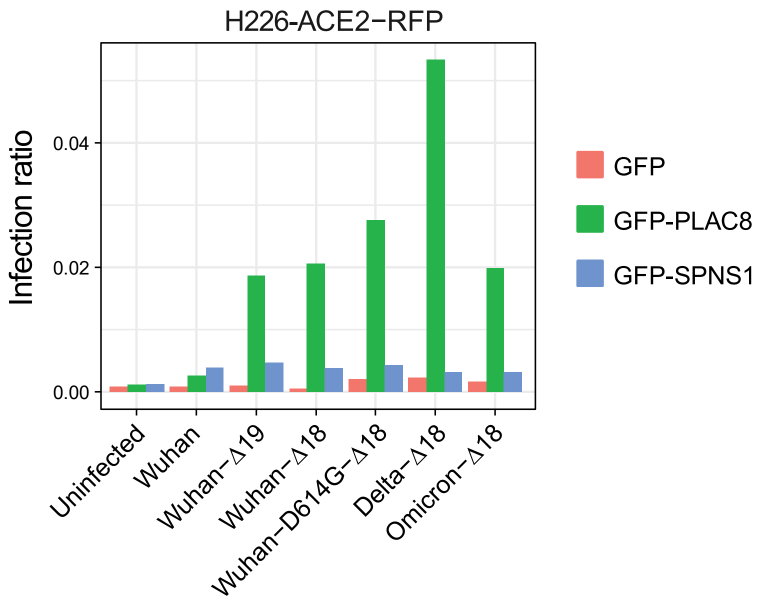

Figure 2: infection efficiency of lentiviruses pseudotyped with different variants of SARS-CoV-2 Spike protein in H226-ACE2-RFP cells that overexpress GFP, GFP-PLAC8 or GFP-SPNS1. Δ18 and Δ19: 18 and 19 c-terminal amino acid deletions relative to SARS-CoV-2 full length Spike protein. Wuhan-Δ19 is the variant used throughout our work.

7. Fig 5F is not convincing because the ratio panel (merged images) is highly saturated as compared to their respective individual images.

Thank you for pointing that out. We always take care of avoiding over-exposition. We double checked the images with the ImageJ HiLow look-up table and only few spots were overexposed. Nonetheless, we have included less exposed versions in the revised manuscript.

8. levels of LC3 and p62 are different in Calu1ACE2 and H1299ACE2 cell lines and not much significant after knocking down of PLAC8?

In the revised manuscript, we have performed an extensive characterization of autophagy using more suitable techniques and experiments. We have replaced the Western-blot analyses of the original manuscript with a more complete immunofluorescence study of LC3B and p62 and autophagic flux analysis with the reporter GFP-LC3-RFP-LC3ΔG (Figure 6C-F, S7 and S8 in the revised manuscript). In addition, we have extended these studies to cell lines with GFP-PLAC8 and GFP-SPNS1 overexpression. This set of experiments have helped us to reach a more detailed picture of the role of the autophagolysosomal alterations upon PLAC8 and SPNS1 gain- and loss-of-function in the context of SARS-CoV-2 infection.

Minor points:

1. Figure 3A. The y axis title should be "ratio", not "percentage"

We have fixed this issue in the revised manuscript.

2. Autophagy and lysosomal function are involved in SARS-CoV-2 replication and release but not entry. Not sure this figure could link the function of the two factors and viral entry.

As explained in response to point 8, our revised manuscript explores in much more detail the role of autophagolysosomal alterations upon PLAC8 and SPNS1 gain- and loss-of-function in the context of SARS-CoV-2 infection. All these new developments have been discussed in the revised manuscript.

Referee #3:

To identify targetable host factors for SARS CoV2 infections Ugalde et al performed a Crispr screen in human Calu1 lung cancer cells (overexpressing ACE2) using pseudotyped lentivirus. The authors pulled out two new factors, namely SPNS1 and PLAC8, that in loss of function experiments were confirmed to be involved in viral entry, including infections with the fully infectious SARS-CoV-2 virus. PLAC8 is highly expressed in ciliated and secretory cells from the respiratory tract and in gut enterocytes and its overexpression facilitates viral entry. Mechanistically the authors propose that SPNS1 and PLAC8 affect viral entry through regulation of autophagy and lysosomal function.

Though the proposed pathway is not novel and many questions remain on detailed mechanisms, this is certainly an interesting set of well conducted experiments that identify novel regulators of SARS-CoV-2.

I am not expert on Crispr screening (genome wide p values, proper controls, library used etc.) but the screen was done in any case to identify novel players in viral entry. The validation of the SPNS1 and PLAC8 knockout clones and their rescues, in particular, are very convincing. One question: in S. Fig. 1C, was the ACE2 clone (I assume the red bar is an ace2 wild type parent clone and the green bar an ace2 KO clone, would be good to describe this better in the figure legend) an incomplete knockout clone considering that viral entry is only reduced by about 40%.

For reporting the data in Fig. 2B etc, "Bars represent the average and standard error of the mean (SEM) of the percentage of infected cells in each condition (three replicates), normalized to the non-targeting control cell line NT596." Was the intensity of the zsGREEN signal also reduced in the remaining cells that were infected or is this an all or none phenomenon? If the latter is true, how do you explain this - incomplete knockouts? Please discuss this, unless I missed it somehow.

ZsGreen expression occurs in cells that have integrated the lentiviral plasmid delivered by the Spike-typed lentivirus. We used an intensity cutoff based on a non-infected sample to distinguish between infected and non-infected cells. While the histogram for non-infected samples has the classic bell shape, ZsGreen intensity histograms in samples from infected cells have a binormal distribution with two clear peaks (one that matches the single peak of a non-infected sample and a clearly shifted one that correspond to infected cells). Therefore, the readout is closer to an all or none phenomenon with a positional effect variability (differences in ZsGreen expression arising from the site of genomic insertion). The attached Figure 3 bellow is an example of a typical intensity histogram from cells co-infected with mCherry VSVG-typed and ZsGreen Spike-typed viruses.

Regarding the “incomplete knockouts”, we do not see any discrepancy with the observed data. All the cellular models used in this work are polyclonal, not single clones. Although the knock-out efficiency is very high according to our Western-blot analyses, this technique only gives the average protein levels in the whole population, but there will be for sure wild-type or haploinsufficient cells that can be infected.

We hope we understood and addressed your question correctly.

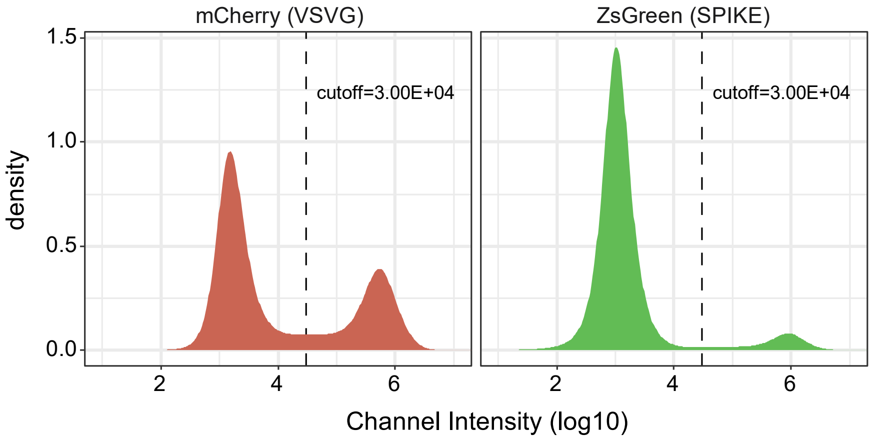

Figure 3: representative flow-cytometry intensity histograms of mCherry and ZsGreen fluorescence in cells infected with mCherry-VSVG-typed and ZsGreen-Spike-typed lentiviruses.

As for the SARS-CoV2 infections in Figure 3, it would be important to also assess viral RNA in addition to "immunofluorescence of nucleocapsid (N) protein 24 h post infection (p.i.)." After all, if the hypothesis of the authors is correct then there should be also a marked decrease in viral replication.

We chose nucleocapsid immunofluorescence because it is a highly abundant protein in SARS-CoV-2 infected cells and there are polyclonal antibodies that provide an excellent signal. Nucleocapsid immunofluorescence has been used extensively throughout SARS-CoV-2 literature. In contrast, we reasoned that immunofluorescence of double strand RNA would yield a lower signal to noise ratio than nucleocapsid detection. In addition, since our screen only addresses viral entry, we did not find relevant to look at viral replication.

1st Revision - Editorial Decision **5th Aug 2022**

Dear Alejandro,

Thank you for submitting your revised manuscript to The EMBO Journal. I am sorry about the delay in getting back to you, but I have now heard back from referees #1 and 2.

While referee #1 is happy with the revised version, referee #2 has some remaining concerns with the revision. In light of these comments, I asked referee #1 to take a look at the points and have now heard back from the referee. Referee #1 appreciates the points raised by referee #2, but also find that you have responded to the raised concerns in a satisfactory manner. I have also looked at the concerns raised and I am in agreement with referee #1.

Given this I am therefore very pleased to let you know that we will accept this MS for publication here. No further experimental changes are needed. Before sending you the formal accept letter, there are just a few editorial points that we need to sort out:

- Please include a point-by-point response to the remaining points of referee #2.

- Please add 3-5 keywords

- COI needs to be re-labelled as Disclosure statement and competing interests

- Please double check the grant number give for SAF2017-87655-R. Has different numbers in MS text vs online submission system.

- The ToC for the appendix needs page numbers adding. The nomenclature needs to be corrected to "Appendix Figure S#" and "Appendix Table S#" throughout. Appendix Table S2 is labelled "Appendix Table S4".

- In the data availability section please make the data sets available and remove referee tokes. Also please update the sentence" scRNA-Seq datasets of from lung , ileum and colon samples from healthy donors were downloaded from the UCSC Cell Browser (Karin: please add). The datasets of lung samples from COVID-19 patients were downloaded from the UCSC Cell Browser and from (Karin please add).

- We encourage the publication of source data, particularly for electrophoretic gels and blots but also for quantitative data, with the aim of making primary data more accessible and transparent to the reader. The source data should be uploaded as one file per figure. For blots please provide the original, uncropped and unprocessed scans and molecular weight markers; further annotation could be useful but is not essential. If multiple different types of files per figure zip them into one file per figure. The files should be labelled with the appropriate figure/panel number. The files will be published online with the article as supplementary "Source Data" files.

- Our publisher has also done their pre-publication check on your manuscript. When you log into the manuscript submission system you will see the file "Data Edited Manuscript file". Please take a look at the word file and the comments regarding the figure legends and respond to the issues.

- We include a synopsis of the paper (see http://emboj.embopress.org/). Please provide me with a general summary statement and 3-5 bullet points that capture the key findings of the paper.

- We also need a summary figure for the synopsis. The size should be 550 wide by [200-400] high (pixels). You can also use something from the figures if that is easier.

That should be all if you have any further questions contact me

With best wishes

Karin

Karin Dumstrei, PhD
Senior Editor
The EMBO Journal

Guide For Authors: https://www.embopress.org/page/journal/14602075/authorguide

Use the link below to submit your revision:

https://emboj.msubmit.net/cgi-bin/main.plex

Referee #1:

The authors have adequately addressed my concerns through experimental efforts. This study convincingly identifies new cellular factors involved in SARS-CoV-2 infections and links this effect to regulation of autophagy. This is a novel and important contribution to the field.

Referee #2:

We appreciate the efforts authors made to address previously raised questions. However, major concerns were not resolved in this revision as outlined below.

1. The answers are disappointing. Purified RBD of Spike can't mimic the entry of SARS-CoV-2 because viral entry is mediated by the full length S1 and S2 with correct conformation of spike trimer. RBD alone (partial of S1) is not capable to mimic the functional full-length spike on virions. Therefore, the new data can't help making a solid conclusion about whether viral binding or internalization is affected when PLCA8/SPNS1 is knocked out.

2. These results are very confusing. This is a well-known fact in the field that TMPRSS2 is not expressed in A549 and Vero cells but highly expressed in Calu3 and differentiated primary NHBE cells. This phenomenon is confirmed by several labs and well documented in published literature. If the authors want to overturn this well-established conclusion, they should provide robust evidence like TMPRSS2 protein/mRNA evaluation in TMRPSS2 KO/KD conditions through multiple strategies. A simple WB is not sufficient and convincing.

Question 3 & 4: Because question 1 was not addressed in an appropriate way, there is not solid conclusion on it. Therefore, other associated questions are still there.

5: This question was not addressed.

6. This is the easiest and functionally relevant question to answer in the entire revision. Simply package MERS and SARS-1 pseudovirus and test them in the KO cells and we will know whether these two factors are involved in viral entry on the coronaviruses. Live virus is not required at all.

Referee #1:

The authors have adequately addressed my concerns through experimental efforts. This study convincingly identifies new cellular factors involved in SARS-CoV-2 infections and links this effect to regulation of autophagy. This is a novel and important contribution to the field.

We are pleased to hear that. We would like to thank the reviewer for his/her constructive criticisms and his/her positive comments on our work.

Referee #2:

We appreciate the efforts authors made to address previously raised questions. However, major concerns were not resolved in this revision as outlined below.

1. The answers are disappointing. Purified RBD of Spike can't mimic the entry of SARS-CoV-2 because viral entry is mediated by the full length S1 and S2 with correct conformation of spike trimer. RBD alone (partial of S1) is not capable to mimic the functional full-length spike on virions. Therefore, the new data can't help making a solid conclusion about whether viral binding or internalization is affected when PLCA8/SPNS1 is knocked out.

We are sorry to hear that. We are well aware that reductionist approaches have limitations. One could also argue that soluble recombinant full-length trimeric Spike cannot recapitulate the biology of a virion. However, we do think that our experiments with Spike RBD provide helpful information to narrow down the underlying molecular mechanisms of these novel host factors. Recombinant Spike RBD has been widely used in multiple works to study different aspects of Spike/ACE2 interaction (e.g Tai *et al*, 2020; Cao *et al*, 2022, 2). It is well-known that ACE2/SPIKE interaction is dictated by residues within the RBD, and as such, we think is fair to conclude from our binding experiments with S-RBD and the measurements of cell surface ACE2, that PLAC8 and SPNS1 do not play a role in this process. Regarding endocytosis, our experiments show that S-RBD is internalized in an ACE2-dependent manner and that PLAC8 and SPNS1 loss-of-function do not affect this process. This result probes that these novel host factors are not involved in ligand-triggered ACE2 internalization, the first step of SARS-CoV-2 infection. Further evidence of an intracellular role of these novel host factors is the fact that we see colocalization of internalized ACE2/S-RBD and PLAC8 and SPNS1. Other works have shown that binding of recombinant SARS-CoV or SARS-CoV-2 Spike RBD alone is sufficient to

induce the internalization of ACE2 (Bruno *et al*, 2018; Wang *et al*, 2008; Inoue *et al*, 2007) in a similar manner that full-length Spike (Bayati *et al*, 2021).

The main message of this paper is that we have unveiled two unknown novel host factors that were validated orthogonally with multiple loss- and gain-of-function experiments using full SARS-CoV-2 viruses in multiple cell lines. The functional relevance of these two genes, and specially PLAC8, is very solid. Science is built on cumulative knowledge and cooperation. We think that withholding our findings until we reach a full understanding of the underlying molecular mechanisms would impact negatively on science by hampering other labs to build from our discoveries.

2. These results are very confusing. This is a well-known fact in the field that TMPRSS2 is not expressed in A549 and Vero cells but highly expressed in Calu3 and differentiated primary NHBE cells. This phenomenon is confirmed by several labs and well documented in published literature. If the authors want to overturn this well-established conclusion, they should provide robust evidence like TMPRSS2 protein/mRNA evaluation in TMRPSS2 KO/KD conditions through multiple strategies. A simple WB is not sufficient and convincing.

In this work we have identified PLAC8 and SPNS1 as novel host factors for SARS-CoV-2 infection. TMPRSS2 did not pop up in our screen. Although we could agree with the reviewer that studying the TMPRSS2 dependency of PLAC8 and SPNS1 might be interesting, we do not think that these would change the message of our work: PLAC8 is a key host factor for SARS-CoV-2 infection. It would probably be equally interesting to study the dependency on CTSL, which is a top hit in our screen and proposed to be highly important for Spike activation. Nonetheless, in this question the reviewer stated that none of our cell lines express TMPRSS2 (a reference supporting this statement would have been helpful) and therefore we included a Western-blot that shows otherwise, using a literature-validated antibody (Koch *et al*, 2021; Klouda *et al*, 2022; David *et al*, 2022). The bands fit with the expected molecular weight, and we see also cleaved TMPRSS2. We don't make any statement that try to overturn any well-stablished conclusion about TMPRSS2. In our manuscript we just state that TMPRSS2 was measured and was found expressed in most of our cellular models. A549, VeroE6 and Calu-3 were not vehicle in our manuscript and were only included for comparison in the revised manuscript.

We took this criticism very seriously and carried out an intensive search for evidence in the literature regarding TMPRSS2 expression in our cellular models. The figure below summarizes

our findings. We apologize if we have overlooked any publication with relevant information on this matter. In general, it was hard to find works where the protein levels of TMPRSS2 were compared by western-blot in some of our cellular models. Surprisingly, we also found many works where statements about the expression levels of TMPRSS2 in their cellular models were not supported by experiments or citations (e.g. Hoffmann *et al*, 2020; Meng *et al*, 2022). As we mentioned in our previous response, Koch et al (Koch *et al*, 2021) show a black and white difference between A549 and Calu-3. A difference with this study is that they used Vero cells and ACE2-expressing A549, while we used Vero E6 and parental A549 (without ACE2 overexpression). However, we also found works that describe a different scenario. For example, Saccon et al. and Sagar et al. show similar levels of TMPRSS2 in Calu-3, A549 and VeroE6 cell lines (Saccon *et al*, 2021; Sagar *et al*, 2021). On the other hand, Kim et al. show higher levels of TMPRSS2 in Calu-3 than in A549, but they show strong signal in Vero cells (Kim *et al*, 2021). Barret et al. detect higher levels of TMPRSS2 in A549 than in Vero cells (Barrett *et al*, 2021). We also found pieces of evidence that contradict the idea that none of our cells express TMPRSS2. For example, Liu et al. use H1299 to study G-quadruplex regulation of TMPRSS2 expression and they show high levels of TMPRSS2 in untreated cells (Liu *et al*, 2022). Ou et al. also show signal for TMPRSS2 in H1299 cells, with levels similar to Calu-3 (Ou *et al*, 2021). Finally, Pozzi et al. used Calu-1 cells to study TMPRSS2 inhibition by hydrogen sulfide and show multiple western-blots with strong signal for TMPRSS2 (Pozzi *et al*, 2021). In summary, the data we have found show important discrepancies within the literature, suggesting that TMPRSS2 expression and/or detection is influenced by the experimental procedures.

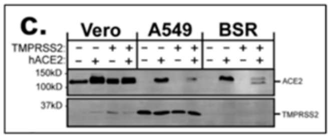

Fig S2C (Barret et al, JBC, 2021, 297(1):100902)

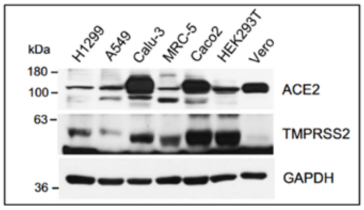

Fig 6A (Kim et al., Experimental & Molecular Medicine (2021) 53:956–972

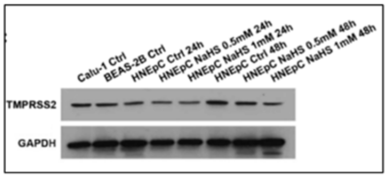

Fig 3C (Pozzi et al, Biomedicines, 2021, 9:1273)

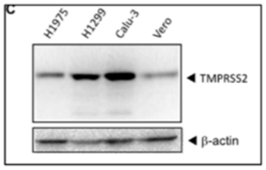

Fig 3C (Ou et al, Plos Pathogenes, 2021, 17(1):e1009212)

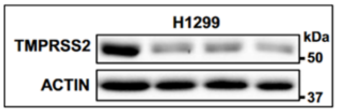

Fig 2A (Liu et al. Nat. Comm. (2022)13:1444

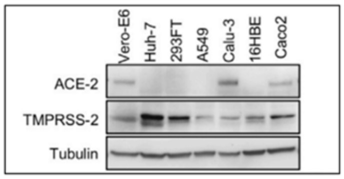

Fig 2B (Saccon et al, iScience 24, 102420, May 21, 2021) (SC-515727)

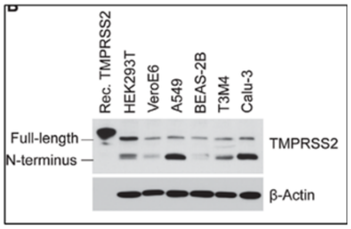

Fig 2B (Sagar et al, Clin. Transl. Med., 2021 Feb;11(2):e281)

Nonetheless, to be on the safe side, we have repeated the Western-blot in Appendix Figure S2C using new cell pellets and including additional controls: A549 and H358 cells expressing human full-length TMPRSS2 variant 1 (addgene plasmid #145843). These cells were generated at the beginning of the project when we were setting up a cellular model for the screen. As it can be seen in the figure below, the results are very similar than those presented in the revised manuscript: most of our cell lines have detectable TMPRSS2 and no large differences in TMPRSS2 protein levels are observed among the cell lines. However, increased levels of TMPRSS2 are very clear in A549 and H358 TMPRSS2-expressing cell lines, which demonstrates the specificity of the antibody. To have a better perspective, we also included LNcAP (Afar *et al*, 2001; Chen *et al*, 2010; Chu *et al*, 2014; Deng *et al*, 2021) and Caco-2 cells (Zhang *et al*, 2020; Cocozza *et al*, 2020; Chen *et al*, 2021; Zeng *et al*, 2022), which are supposed to express TMPRSS2. Please note that lysates from lanes 9-10 and 12-13 (parental and TMPRSS2-expressing A549 and H-358) were done separately with 1-day difference.

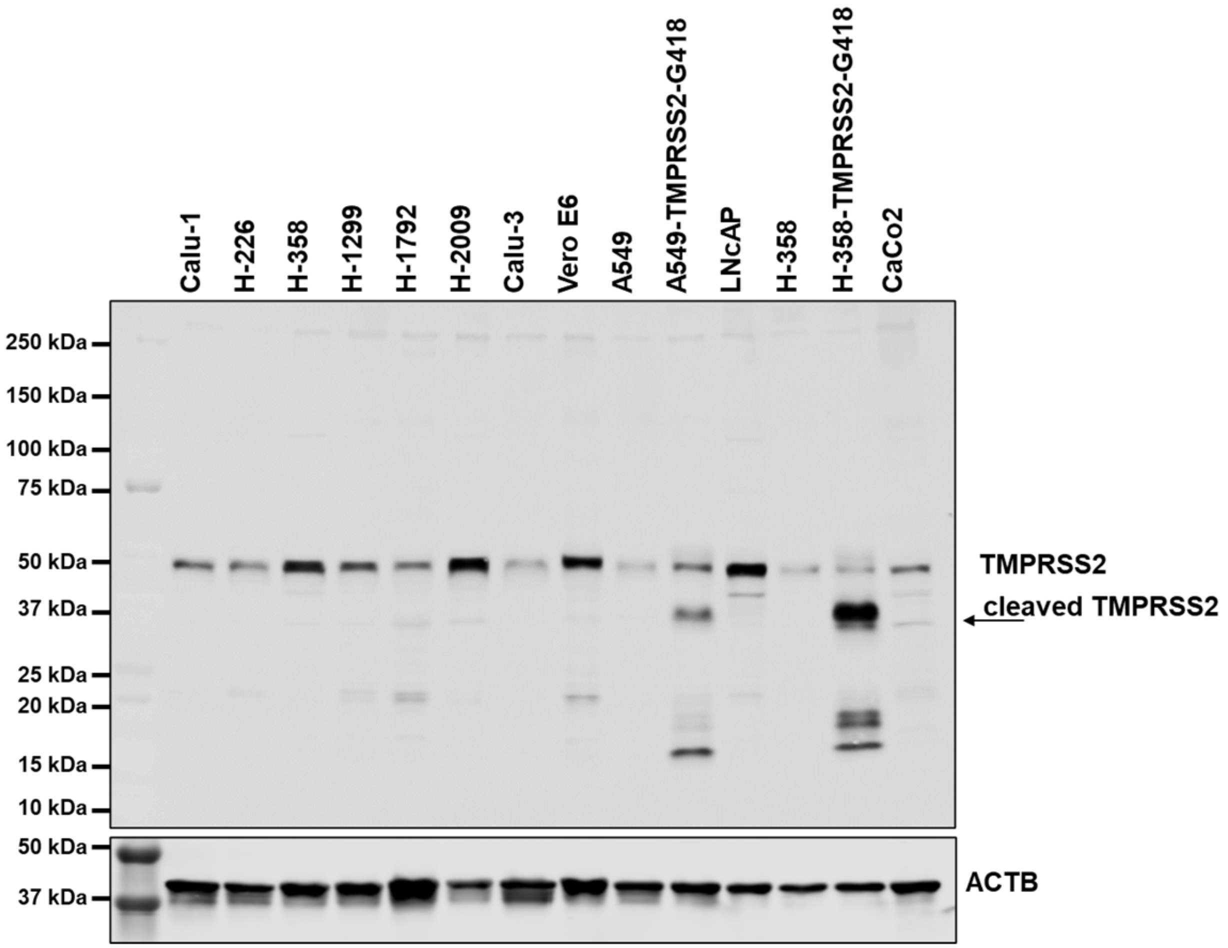

Question 3 & 4: Because question 1 was not addressed in an appropriate way, there is not solid conclusion on it. Therefore, other associated questions are still there.

Independently of question 1, questions 3 and 4 were addressed as follows.

In response to question 3, we provided detailed reasons of why it is very unlikely that PLAC8 antibodies would prevent viral entry. PLAC8 is located at the inner plasma membrane, which renders it inaccessible for an extracellular intervention with antibodies.

Regarding question 4, we performed immunoprecipitation experiments of ACE2 and GFP-PLAC8 in extracts that were incubated with **full-length trimeric Spike protein** (His-tagged). While we captured the interaction between Spike and ACE2, we did not detect a direct interaction between PLAC8 and Spike.

5: This question was not addressed.

Indeed, this question was not addressed experimentally. While we agree with the reviewer that these experiments would provide with additional evidence about these genes, we think our work has multiple solid functional assays that probe that PLAC8, and SPNS1, are important SARS-CoV-2 host factors. We think that performing the requested experiments would unnecessarily delay several months the publication of our findings.

6. This is the easiest and functionally relevant question to answer in the entire revision. Simply package MERS and SARS-1 pseudovirus and test them in the KO cells and we will know whether these two factors are involved in viral entry on the coronaviruses. Live virus is not required at all.

MERS-CoV uses a different receptor and therefore our ACE2-expressing cellular models would be meaningless. We would have to use DPP4 expressing cell lines or overexpress it in our models. In addition, we would have to order ORFs of MERS-CoV and SARS-CoV, and most likely generate C-terminal deletions to improve infection and to make it comparable to SARS-CoV-2 SpikeΔ19. Most importantly, although we agree that these experiments would yield useful information, they will not change the fact that PLAC8 and SPNS1 are host factors for SARS-CoV-2 infection, which is the message of our work. We think it is more important to readily share our findings with the research community so future follow-up studies can address all these associated questions.

REFERENCES

Afar DE, Vivanco I, Hubert RS, Kuo J, Chen E, Saffran DC, Raitano AB & Jakobovits A (2001) Catalytic cleavage of the androgen-regulated TMPRSS2 protease results in its secretion by prostate and prostate cancer epithelia. *Cancer Res* 61: 1686–1692

Barrett CT, Neal HE, Edmonds K, Moncman CL, Thompson R, Branttie JM, Boggs KB, Wu C-Y, Leung DW & Dutch RE (2021) Effect of clinical isolate or cleavage site mutations in the SARS-CoV-2 spike protein on protein stability, cleavage, and cell–cell fusion. *Journal of Biological Chemistry* 297: 100902

Bayati A, Kumar R, Francis V & McPherson PS (2021) SARS-CoV-2 infects cells after viral entry via clathrin-mediated endocytosis. *J Biol Chem* 296: 100306

Bruno G, Cencetti F, Bernacchioni C, Donati C, Blankenbach KV, Thomas D, Meyer Zu Heringdorf D & Bruni P (2018) Bradykinin mediates myogenic differentiation in murine myoblasts through the involvement of SK1/Spns2/S1P(2) axis. *Cell Signal* 45: 110–121

Cao Y, Yisimayi A, Jian F, Song W, Xiao T, Wang L, Du S, Wang J, Li Q, Chen X, *et al* (2022) BA.2.12.1, BA.4 and BA.5 escape antibodies elicited by Omicron infection. *Nature* 608: 593–602

Chen Y, Lear TB, Evankovich JW, Larsen MB, Lin B, Alfaras I, Kennerdell JR, Salminen L, Camarco DP, Lockwood KC, *et al* (2021) A high-throughput screen for TMPRSS2 expression identifies FDA-approved compounds that can limit SARS-CoV-2 entry. *Nat Commun* 12: 3907

Chen Y-W, Lee M-S, Lucht A, Chou F-P, Huang W, Havighurst TC, Kim K, Wang J-K, Antalis TM, Johnson MD, *et al* (2010) TMPRSS2, a serine protease expressed in the prostate on the apical surface of luminal epithelial cells and released into semen in prostasomes, is misregulated in prostate cancer cells. *Am J Pathol* 176: 2986–2996

Chu M, Chang Y, Wang N, Li W, Li P & Gao W-Q (2014) Hypermethylation-mediated transcriptional repression of TMPRSS2 in androgen receptor-negative prostate cancer cells. *Exp Biol Med (Maywood)* 239: 823–828

Cocozza F, Névo N, Piovesana E, Lahaye X, Buchrieser J, Schwartz O, Manel N, Tkach M, Théry C & Martin-Jaular L (2020) Extracellular vesicles containing ACE2 efficiently prevent infection by SARS-CoV-2 Spike protein-containing virus. *J Extracell Vesicles* 10: e12050

David A, Parkinson N, Peacock TP, Pairo-Castineira E, Khanna T, Cobat A, Tenesa A, Sancho-Shimizu V, GenOMICC Consortium, ISARIC4C Investigators, *et al* (2022) A common TMPRSS2 variant has a protective effect against severe COVID-19. *Curr Res Transl Med* 70: 103333

Deng Q, Rasool RU, Russell RM, Natesan R & Asangani IA (2021) Targeting androgen regulation of TMPRSS2 and ACE2 as a therapeutic strategy to combat COVID-19. *iScience* 24: 102254

Hoffmann M, Kleine-Weber H, Schroeder S, Krüger N, Herrler T, Erichsen S, Schiergens TS, Herrler G, Wu N-H, Nitsche A, *et al* (2020) SARS-CoV-2 Cell Entry Depends on ACE2 and TMPRSS2 and Is Blocked by a Clinically Proven Protease Inhibitor. *Cell* 181: 271-280.e8

Inoue Y, Tanaka N, Tanaka Y, Inoue S, Morita K, Zhuang M, Hattori T & Sugamura K (2007) Clathrin-dependent entry of severe acute respiratory syndrome coronavirus into target cells expressing ACE2 with the cytoplasmic tail deleted. *J Virol* 81: 8722–8729

Kim TY, Jeon S, Jang Y, Gotina L, Won J, Ju YH, Kim S, Jang MW, Won W, Park MG, *et al* (2021) Platycodin D, a natural component of Platycodon grandiflorum, prevents both lysosome- and TMPRSS2-driven SARS-CoV-2 infection by hindering membrane fusion. *Exp Mol Med* 53: 956–972

Klouda T, Hao Y, Kim H, Kim J, Olejnik J, Hume AJ, Ayyappan S, Hong X, Melero-Martin J, Fang Y, *et al* (2022) Interferon-alpha or -beta facilitates SARS-CoV-2 pulmonary vascular infection by inducing ACE2. *Angiogenesis* 25: 225–240

Koch J, Uckeley ZM, Doldan P, Stanifer M, Boulant S & Lozach P-Y (2021) TMPRSS2 expression dictates the entry route used by SARS-CoV-2 to infect host cells. *EMBO J* 40: e107821

Liu G, Du W, Sang X, Tong Q, Wang Y, Chen G, Yuan Y, Jiang L, Cheng W, Liu D, *et al* (2022) RNA G-quadruplex in TMPRSS2 reduces SARS-CoV-2 infection. *Nat Commun* 13: 1444

Meng B, Datir R, Choi J, Bradley JR, Smith KGC, Lee JH, Gupta RK, Baker S, Dougan G, Hess C, *et al* (2022) SARS-CoV-2 spike N-terminal domain modulates TMPRSS2-dependent viral entry and fusogenicity. *Cell Reports* 40: 111220

Ou T, Mou H, Zhang L, Ojha A, Choe H & Farzan M (2021) Hydroxychloroquine-mediated inhibition of SARS-CoV-2 entry is attenuated by TMPRSS2. *PLoS Pathog* 17: e1009212

Pozzi G, Masselli E, Gobbi G, Mirandola P, Taborda-Barata L, Ampollini L, Carbognani P, Micheloni C, Corazza F, Galli D, *et al* (2021) Hydrogen Sulfide Inhibits TMPRSS2 in Human Airway Epithelial Cells: Implications for SARS-CoV-2 Infection. *Biomedicines* 9: 1273

Saccon E, Chen X, Mikaeloff F, Rodriguez JE, Szekely L, Vinhas BS, Krishnan S, Byrareddy SN, Frisan T, Végvári Á, *et al* (2021) Cell-type-resolved quantitative proteomics map of interferon response against SARS-CoV-2. *iScience* 24: 102420

Sagar S, Rathinavel AK, Lutz WE, Struble LR, Khurana S, Schnaubelt AT, Mishra NK, Guda C, Palermo NY, Broadhurst MJ, *et al* (2021) Bromelain inhibits SARS-CoV-2 infection via targeting ACE-2, TMPRSS2, and spike protein. *Clin Transl Med* 11: e281

Tai W, He L, Zhang X, Pu J, Voronin D, Jiang S, Zhou Y & Du L (2020) Characterization of the receptor-binding domain (RBD) of 2019 novel coronavirus: implication for development of RBD protein as a viral attachment inhibitor and vaccine. *Cell Mol Immunol* 17: 613–620

Wang S, Guo F, Liu K, Wang H, Rao S, Yang P & Jiang C (2008) Endocytosis of the receptor-binding domain of SARS-CoV spike protein together with virus receptor ACE2. *Virus Res* 136: 8–15

Zeng W, Xing F, Ji Y, Yang S, Xu T, Huang S, Li C, Wu J, Cao L & Guo D (2022) Evidence of Infection of Human Embryonic Stem Cells by SARS-CoV-2. *Front Cell Infect Microbiol* 12: 911313

Zhang S, Liu Y, Wang X, Yang L, Li H, Wang Y, Liu M, Zhao X, Xie Y, Yang Y, *et al* (2020) SARS-CoV-2 binds platelet ACE2 to enhance thrombosis in COVID-19. *J Hematol Oncol* 13: 120

2nd Revision - Editorial Decision 5th Sep 2022

Dear Alejandro,

Thank you for submitting your revised manuscript to The EMBO Journal. I have now had a chance to take a look at it and all looks good.

I am therefore very pleased to accept the manuscript for publication here.

Congratulations on a nice study

Best Karin

Karin Dumstrei, PhD
Senior Editor
The EMBO Journal

Please note that it is EMBO Journal policy for the transcript of the editorial process (containing referee reports and your response letter) to be published as an online supplement to each paper. If you do NOT want this, you will need to inform the Editorial Office via email immediately. More information is available here: https://www.embopress.org/page/journal/14602075/authorguide#transparentprocess

Your manuscript will be processed for publication in the journal by EMBO Press. Manuscripts in the PDF and electronic editions of The EMBO Journal will be copy edited, and you will be provided with page proofs prior to publication. Please note that supplementary information is not included in the proofs.

You will be contacted by Wiley Author Services to complete licensing and payment information. The required 'Page Charges Authorization Form' is available here: https://www.embopress.org/pb-assets/embo-site/tej_apc.pdf - please download and complete the form and return to embopressproduction@wiley.com

Should you be planning a Press Release on your article, please get in contact with embojournal@wiley.com as early as possible, in order to coordinate publication and release dates.

If you have any questions, please do not hesitate to call or email the Editorial Office. Thank you for your contribution to The EMBO Journal.

** Click here to be directed to your login page: https://emboj.msubmit.net

EMBO Press Author Checklist

Corresponding Author Name: Alejandro P. Ugalde
Journal Submitted to: The EMBO Journal
Manuscript Number: EMBOJ-2022-110727

USEFUL LINKS FOR COMPLETING THIS FORM
The EMBO Journal - Author Guidelines
EMBO Reports - Author Guidelines
Molecular Systems Biology - Author Guidelines
EMBO Molecular Medicine - Author Guidelines

Reporting Checklist for Life Science Articles (updated January
This checklist is adapted from Materials Design Analysis Reporting (MDAR) Checklist for Authors. MDAR establishes a minimum set of requirements in transparent reporting in the life sciences (see Statement of Task: 10.31222/osf.io/9sm4x). Please follow the journal's guidelines in preparing your
Please note that a copy of this checklist will be published alongside your article.

Abridged guidelines for figures
1. Data
The data shown in figures should satisfy the following conditions:
- the data were obtained and processed according to the field's best practice and are presented to reflect the results of the experiments in an accurate and unbiased manner.
- ideally, figure panels should include only measurements that are directly comparable to each other and obtained with the same assay.
- plots include clearly labeled error bars for independent experiments and sample sizes. Unless justified, error bars should not be shown for technical
- if n<5, the individual data points from each experiment should be plotted. Any statistical test employed should be justified.
- Source Data should be included to report the data underlying figures according to the guidelines set out in the authorship guidelines on Data

2. Captions
Each figure caption should contain the following information, for each panel where they are relevant:
- a specification of the experimental system investigated (eg cell line, species name).
- the assay(s) and method(s) used to carry out the reported observations and measurements.
- an explicit mention of the biological and chemical entity(ies) that are being measured.
- an explicit mention of the biological and chemical entity(ies) that are altered/varied/perturbed in a controlled manner.
- the exact sample size (n) for each experimental group/condition, given as a number, not a range;
- a description of the sample collection allowing the reader to understand whether the samples represent technical or biological replicates (including how many animals, litters, cultures, etc.).
- a statement of how many times the experiment shown was independently replicated in the laboratory.
- definitions of statistical methods and measures:
  - common tests, such as t-test (please specify whether paired vs. unpaired), simple χ2 tests, Wilcoxon and Mann-Whitney tests, can be unambiguously identified by name only, but more complex techniques should be described in the methods section;
  - are tests one-sided or two-sided?
  - are there adjustments for multiple comparisons?
  - exact statistical test results, e.g., P values = x but not P values < x;
  - definition of 'center values' as median or average;
  - definition of error bars as s.d. or s.e.m.

Please complete ALL of the questions below.
Select "Not Applicable" only when the requested information is not relevant for your study.

Materials

Newly Created Materials	Information included in the manuscript?	In which section is the information available? (Reagents and Tools Table, Materials and Methods, Figures, Data Availability Section)
New materials and reagents need to be available; do any restrictions apply?	Not Applicable	Plasmids will be available upon request

Antibodies	Information included in the manuscript?	In which section is the information available? (Reagents and Tools Table, Materials and Methods, Figures, Data Availability Section)
For **antibodies** provide the following information:<br>- Commercial antibodies: RRID (if possible) or supplier name, catalogue number and or/clone number<br>- Non-commercial: RRID or citation	Yes	Appendix Table S2

DNA and RNA sequences	Information included in the manuscript?	In which section is the information available? (Reagents and Tools Table, Materials and Methods, Figures, Data Availability Section)
**Short novel DNA or RNA including primers, probes**: provide the sequences.	Yes	Appendix Table S1

Cell materials	Information included in the manuscript?	In which section is the information available? (Reagents and Tools Table, Materials and Methods, Figures, Data Availability Section)
**Cell lines:** Provide species information, strain. Provide accession number in repository **OR** supplier name, catalog number, clone number, and/**OR** RRID.	Yes	Material and Methods
**Primary cultures:** Provide species, strain, sex of origin, genetic modification status.	Not Applicable	
Report if the cell lines were recently **authenticated** (e.g., by STR profiling) and tested for mycoplasma contamination.	Yes	Material and Methods

Experimental animals	Information included in the manuscript?	In which section is the information available? (Reagents and Tools Table, Materials and Methods, Figures, Data Availability Section)
**Laboratory animals or Model organisms:** Provide species, strain, sex, age, genetic modification status. Provide accession number in repository **OR** supplier name, catalog number, clone number, **OR** RRID.	Not Applicable	
**Animal observed in or captured from the field:** Provide species, sex, and age where possible.	Not Applicable	
Please detail **housing and husbandry conditions**.	Not Applicable	

Plants and microbes	Information included in the manuscript?	In which section is the information available? (Reagents and Tools Table, Materials and Methods, Figures, Data Availability Section)
**Plants:** provide species and strain, ecotype and cultivar where relevant, unique accession number if available, and source (including location for collected wild specimens).	Not Applicable	
**Microbes:** provide species and strain, unique accession number if available, and source.	Yes	Material and Methods

Human research participants	Information included in the manuscript?	In which section is the information available? (Reagents and Tools Table, Materials and Methods, Figures, Data Availability Section)
If collected and within the bounds of privacy constraints report on age, sex and gender or ethnicity for all study participants.	Not Applicable	

Core facilities	Information included in the manuscript?	In which section is the information available? (Reagents and Tools Table, Materials and Methods, Figures, Data Availability Section)
If your work benefited from core facilities, was their service mentioned in the acknowledgments section?	Yes	Acknowledgments

Design

Study protocol	Information included in the manuscript?	In which section is the information available? (Reagents and Tools Table, Materials and Methods, Figures, Data Availability Section)
If study protocol has been **pre-registered, provide DOI in the manuscript**. For clinical trials, provide the trial registration number **OR** cite DOI.	Not Applicable	
Report the **clinical trial registration number** (at ClinicalTrials.gov or equivalent), where applicable.	Not Applicable	

Laboratory protocol	Information included in the manuscript?	In which section is the information available? (Reagents and Tools Table, Materials and Methods, Figures, Data Availability Section)
Provide DOI OR other citation details if **external detailed step-by-step protocols** are available.	Not Applicable	

Experimental study design and statistics	Information included in the manuscript?	In which section is the information available? (Reagents and Tools Table, Materials and Methods, Figures, Data Availability Section)
Include a statement about **sample size** estimate even if no statistical methods were used.	Yes	Material and Methods
Were any steps taken to minimize the effects of subjective bias when allocating animals/samples to treatment (e.g. **randomization procedure**)? If yes, have they been described?	Yes	Material and Methods
Include a statement about **blinding** even if no blinding was done.	Yes	Material and Methods
Describe **inclusion/exclusion criteria** if samples or animals were excluded from the analysis. Were the criteria pre-established?<br><br>If sample or data points were omitted from analysis, report if this was due to attrition or intentional exclusion and provide justification.	Yes	Material and Methods
For every figure, are **statistical tests** justified as appropriate? Do the data meet the assumptions of the tests (e.g., normal distribution)? Describe any methods used to assess it. Is there an estimate of variation within each group of data? Is the variance similar between the groups that are being statistically compared?	Yes	Statistical test are not justified in every figure legend. Statistical methods and any assumptions made are described in "Experimental study design and statistics" secction in Material and Methods

Sample definition and in-laboratory replication	Information included in the manuscript?	In which section is the information available? (Reagents and Tools Table, Materials and Methods, Figures, Data Availability Section)
In the figure legends: state number of times the experiment was **replicated** in laboratory.	Yes	Material and Methods
In the figure legends: define whether data describe **technical or biological replicates**.	Yes	Figure legends

Ethics

Ethics	Information included in the manuscript?	In which section is the information available? (Reagents and Tools Table, Materials and Methods, Figures, Data Availability Section)
Studies involving **human participants**: State details of **authority granting ethics approval** (IRB or equivalent committee(s), provide reference number for approval.	Not Applicable	
Studies involving **human participants**: Include a statement confirming that **informed consent** was obtained from all subjects and that the experiments conformed to the principles set out in the WMA Declaration of Helsinki and the Department of Health and Human Services Belmont Report.	Not Applicable	
Studies involving **human participants:** For publication of **patient photos**, include a statement confirming that consent to publish was obtained.	Not Applicable	
Studies involving experimental **animals**: State details of **authority granting ethics approva**l (IRB or equivalent committee(s), provide reference number for approval. Include a statement of compliance with ethical regulations.	Not Applicable	
Studies involving **specimen and field samples:** State if relevant **permits** obtained, provide details of authority approving study; if none were required, explain why.	Not Applicable	

Dual Use Research of Concern (DURC)	Information included in the manuscript?	In which section is the information available? (Reagents and Tools Table, Materials and Methods, Figures, Data Availability Section)
Could your study fall under dual use research restrictions? Please check biosecurity documents and list of **select agents and toxins** (CDC): https://www.selectagents.gov/sat/list.htm	Not Applicable	
If you used a select agent, is the security level of the lab appropriate and reported in the manuscript?	Not Applicable	
If a study is subject to dual use research of concern regulations, is the name of the **authority granting approval and reference number** for the regulatory approval provided in the manuscript?	Not Applicable	

Reporting

The MDAR framework recommends adoption of discipline-specific guidelines, established and endorsed through community initiatives. Journals have their own policy about requiring specific guidelines and recommendations to complement MDAR.

Adherence to community standards	Information included in the manuscript?	In which section is the information available? (Reagents and Tools Table, Materials and Methods, Figures, Data Availability Section)
State if relevant guidelines or checklists (e.g., **ICMJE, MIBBI, ARRIVE, PRISMA**) have been followed or provided.	Not Applicable	None of those guidelines or checklist have been used
For **tumor marker prognostic studies,** we recommend that you follow the **REMARK** reporting guidelines (see link list at top right). See author guidelines, under 'Reporting Guidelines'. Please confirm you have followed these guidelines.	Not Applicable	
For **phase II and III randomized controlled trials**, please refer to the **CONSORT** flow diagram (see link list at top right) and submit the CONSORT checklist (see link list at top right) with your submission. See author guidelines, under 'Reporting Guidelines'. Please confirm you have submitted this list.	Not Applicable	

Data Availability

Data availability	Information included in the manuscript?	In which section is the information available? (Reagents and Tools Table, Materials and Methods, Figures, Data Availability Section)
Have **primary datasets** been deposited according to the journal's guidelines (see 'Data Deposition' section) and the respective accession numbers provided in the Data Availability Section?	Yes	Data Availability
Were **human clinical and genomic datasets** deposited in a public access-controlled repository in accordance to ethical obligations to the patients and to the applicable consent agreement?	Not Applicable	
Are **computational models** that are central and integral to a study available without restrictions in a machine-readable form? Were the relevant accession numbers or links provided?	Not Applicable	
If publicly available data were reused, provide the respective **data citations in the reference list**.	Yes	References
